# Total Synthesis of Feglymycin Using Umpolung Amide Synthesis

**DOI:** 10.1002/anie.202508819

**Published:** 2025-07-26

**Authors:** Preston C. Gourville, Jade A. Bing, Rashanique D. Quarels, Sergey V. Tsukanov, Kenneth E. Schwieter, Kazuyuki Tokumaru, Amanda B. Stephens, Dawn M. Makley, Bo Shen, Abigail N. Smith, Jeffrey N. Johnston

**Affiliations:** ^1^ Department of Chemistry, Institute of Chemical Biology Vanderbilt University Nashville TN USA

**Keywords:** Antiviral, Enantioselective amide synthesis, Feglymycin, Natural product synthesis, Umpolung amide synthesis

## Abstract

The preparation of peptidic molecules is a mainstay of synthesis, creating new tools that advance chemical biology, catalysis, and drug discovery. Despite the wide adoption of methods for amide synthesis based on electrophilic acyl transfer reactions, significant limitations remain that restrict access to chemical space and plague accessible peptides with imperfect conservation of stereochemical information. These problems persist in key applications (i.e., solid phase peptide synthesis) where reagent excess can be used to drive maximal yield. As a general tactic, however, use of excess coupling agents that are often hazardous is unsustainable. Here we report the synthesis of the antiviral tridecapeptide feglymycin where half of the amides are formed using umpolung amide synthesis (UmAS) to replace conventional amide synthesis. Reliance on UmAS further allowed the enantioselective synthesis of each noncanonical residue from an inexpensive aldehyde. As a result, the most process‐intensive components were simplified to a chiral Brønsted acid organocatalyst and potassium iodide/urea·hydrogen peroxide (KI/UHP). This solution‐phase total synthesis illustrates the harmonious, strategic application of complementary amide synthesis methods, and it serves as a touchstone for the green synthesis of peptides composed of noncanonical amino amides.

## Graphical abstract

A total synthesis of the antiviral feglymycin is described, leveraging enantioselective umpolung amide synthesis (UmAS). The strategic use of UmAS to prepare the aryl glycine‐rich tridecapeptide reduced hazardous amide coupling agents by 50%, and replaced asymmetric synthesis reliant on toxic transition metals by organocatalysis.

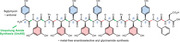

The aryl glycinamide substructure features prominently in natural products with antibacterial and antiviral activity, as well as drugs (e.g., amoxicillin) critical to the fight against infectious disease.^[^ ^1^ ^]^ The heptapeptide vancomycin is a particularly prominent member among aryl glycinamide‐rich peptides which range in size and degree of side‐chain cross‐linking.^[^ ^2^, ^3^, ^4^ ^]^ Vancomycin‐class analogs have been further optimized and endowed with orthogonal mechanisms of action (i.e., maximycins), made possible only by remarkable feats of chemical synthesis.^[^ ^5^ ^]^ A shortcoming of existing amide synthesis technology is the epimerization liability, a risk that is enhanced when using aryl glycine donors. Despite decades of development aimed at coupling agent optimization to enhance the rate of N─C bond formation relative to epimerization, the use of reagent excess remains endemic to the electrophilic acyl transfer paradigm.^[^ ^6^, ^7^, ^8^, ^9^ ^]^ These perils were evident in two landmark syntheses of feglymycin (Figure 1a), an aryl glycinamide‐rich tridecapeptide.^[^ ^10^, ^11^ ^]^ Considering this context,^[^ ^12^ ^]^ we hypothesized that umpolung amide synthesis (UmAS)^[^ ^13^ ^]^ could be integrated alongside traditional couplings to address these needs, especially with aryl glycinamide residues (Figure 1b). Here we report a total synthesis of feglymycin and the first adaptation of the UmAS paradigm to a complex natural product. Six traditional amide formations are replaced with UmAS, resulting in a 50% decrease in DEPBT^[^ ^14^ ^]^ per mmol feglymycin prepared. Furthermore, the aryl glycine equivalents were prepared using enantioselective organocatalysis, replacing transition metals (i.e., osmium) and chiral auxiliaries used in previous approaches.

**Figure 1:**
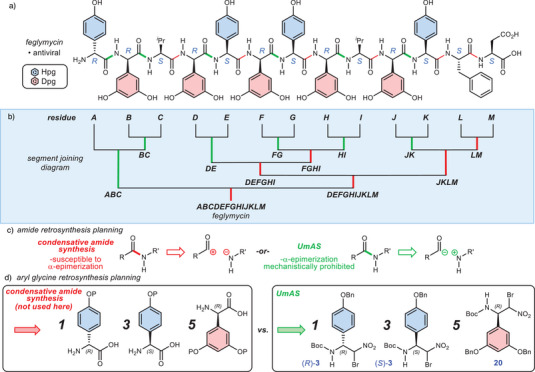
a) The antiviral natural product feglymycin (**1**) and b) segment joining approach using a combination of traditional condensative amide synthesis (red) and umpolung amide synthesis (UmAS, green). c) Comparison of conventional amide synthesis and UmAS based on atomic polarization during C─N bond formation. d) Comparison of Hpg and Dpg residue precursors from retrosynthetic planning using traditional amide synthesis (red) and umpolung amide synthesis (green).

Our approach is outlined in Figure 1b, segmenting feglymycin at the valine residues to provide three peptides (**A**‐**C**, **D**‐**I**, and **J**‐**M**) and an initial level of convergency (3 + 6 + 4) that could be further enhanced by the division of the central hexapeptide into three dipeptides (2 + 2 + 2), two of which are identical. Residues **BC** and **HI** are also identical, offering further efficiency via their common preparation. In order to leverage UmAS (Figure 1c) to mechanistically eliminate epimerization during aryl glycinamide formation, these disconnections require the three aryl glycine donors listed in Figure 1d (right). Residues (*R*)‐Hpg, (*S*)‐Hpg, and (*R*)‐Dpg are found in feglymycin in a 1:3:5 ratio, and prior approaches rely entirely on protected forms of the parent α‐amino acids (Figure 1d, left). The actual ratio required for feglymycin synthesis would be dependent on their point of incorporation into the longest linear sequence (LLS), and of course, the yield of incorporation and all subsequent yields leading to feglymycin. UmAS uses nitroalkane donors (Figure 1d, UmAS) that would be subject to the same effects of incorporation point and yield, but remove the possibility of epimerization during aryl glycinamide formation.^[^ ^15^, ^16^ ^]^ Overall, this approach maximized the advantage of traditional amide bond formation when larger polar peptides are prepared, coupling at canonical amino acid residues, while leveraging enantioselective catalysis to concisely prepare noncanonical residues.

Figure 2 details the longest linear sequence (LLS) for this synthesis of feglymycin, engaging the aryl glycine residue from the beginning. Among noncanonical amino acids, aryl glycine residues have drawn significant synthetic attention,^[^ ^17^ ^]^ remain nontrivial to prepare,^[^ ^18^ ^]^ and are tedious to incorporate into complex peptide synthesis.^[^ ^19^ ^]^ Short sequences to the carboxylic acid donor often rely on expensive chemical feedstock, while inexpensive feedstock must be processed through sequences of five steps or more.^[^ ^20^ ^]^ α‐Amido sulfone **1** was prepared from commercial *para*‐benzyloxy benzaldehyde, and then treated under basic conditions to form the electrophilic aldimine **2** for the subsequent catalyzed addition of bromo nitromethane by (*S*,*S*)‐PBAM. The α‐bromo nitroalkane was isolated in 68% yield and high ee for each diastereomer (1:1.2 dr, 88/90% ee).^[^ ^21^ ^]^ This material could be recrystallized to provide **3** as a mixture of diastereomers (>98% ee each) at the nitro‐substituted carbon. These diastereomers converged in the next step where UmAS conditions provided enantiomerically pure *N*‐methyl amide **4** in 67% yield. Following HCl/dioxane mediated deprotection, this Hpg unit was homologated using UmAS conditions and donor **20** to provide **6** in 66% yield. Donor **20** was prepared from 3,5‐dibenzyloxy benzaldehyde using the identical sequence of reactions for preparation of **3**, except that the catalyst antipode was used ((*R*,*R*)‐PBAM) (Figure 3a). The nitroalkane donor (**20**) was formed in 92% ee (each diastereomer) and 76% yield from **18**, and it was also readily recrystallized. The *N*‐methyl amide was conveniently saponified after chemoselective nitrosation^[^ ^22^ ^]^ in this synthesis to form carboxylic acid **8** in 98% yield (2 steps), prior to removal of the benzyl ethers by hydrogenolysis to furnish **9**. Donor **20** was also coupled to the benzyl ester of valine using UmAS conditions, delivering the **HI** dipeptide in 74% yield (Figure 3a). Deprotection of the Boc group in **21** to reveal the amine (**22**) preceded homologation with acid **9** to give tetrapeptide **10** (Figure 2). *N*‐Deprotection to **11** allowed a second homologation using **9**, followed by hydrogenolysis to furnish hexapeptide **13**.

**Figure 2:**
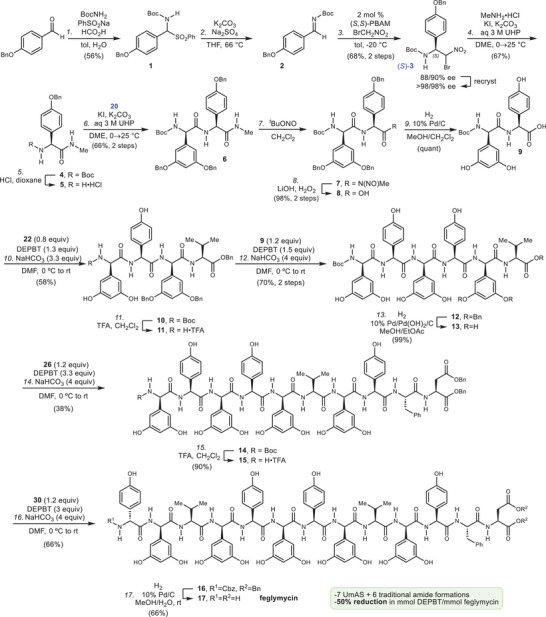
A total chemical synthesis of feglymycin: longest linear sequence (LLS).

**Figure 3:**
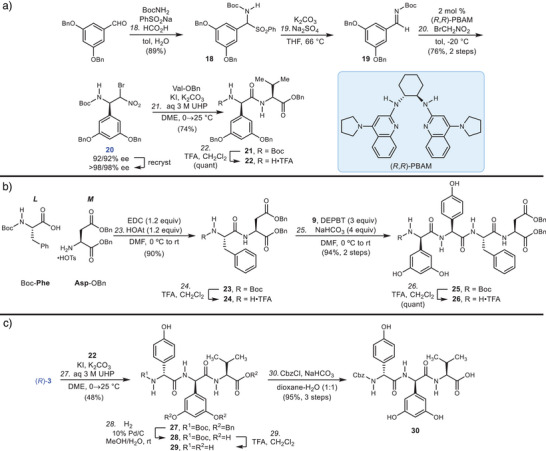
a) Preparation of Dpg‐Val dipeptide (**BC** & **HI**), b) the **JKLM** tetrapeptide, and c) the **ABC** tripeptide.

Separately, Boc‐Phe and Asp‐OBn were coupled using EDC/HOAt to give **23** in 90% yield (Figure 3b). Deprotection of **23** with trifluoroacetic acid (TFA) gave dipeptide **24** which was homologated with dipeptide **9** to give **26** after Boc‐deprotection. Carboxylic acid **13** was activated with DEPBT in its coupling with **26** to produce decapeptide **14** in protected form. TFA was effective for *N*‐deprotection, and **15** so‐produced was homologated with **30**. Tripeptide **30** was synthesized from **22** that had already served in the formation of **10** within the hexapeptide synthesis (Figure 3c). Donor (*R*)‐**3** was prepared from (*R*,*R*)‐PBAM in a sequence otherwise identical to the preparation of (*S*)‐**3**, and then subjected to UmAS conditions for homologation of **22**. The resulting tripeptide **27** required three steps to prepare *N*‐Cbz protected **30**. The final step involved deprotection of *N*‐Cbz and benzyl esters to form synthetic feglymycin, which was identical spectroscopically to natural feglymycin.

The significance of replacing traditional couplings with an umpolung method extends beyond the removal of epimerization risk.^[^ ^23^ ^]^ The tremendous waste,^[^ ^6^, ^24^ ^]^ and hazards^[^ ^25^ ^]^ associated with condensative amide synthesis are widely recognized and lamented.^[^ ^26^, ^27^, ^28^ ^]^ Unfortunately, these drivers of innovation have led mostly to incremental improvements within the classic condensative amide synthesis paradigm. Chiral α‐substituted, electrophilic acyl donors remain problematic with less reactive amines, with epimerization often attenuated but not fully prevented. This was illustrated in total syntheses of feglymycin by Süssmuth^[^ ^9^ ^]^ and Fuse.^[^ ^10^ ^]^ The former synthesis successfully maximized convergency and noted that the coupling agent DEPBT^[^ ^29^ ^]^ was uniquely effective at suppressing (but not eliminating) epimerization during aryl glycinamide formation. Cumulatively, the Süssmuth synthesis required 169 mmol DEPBT per mmol feglymycin produced, while the Fuse synthesis required 751 mmol triphosgene per mmol feglymycin. By comparison, this synthesis of feglymycin was accomplished in 14 steps along the longest linear sequence (LLS) from a material (**3**) equivalent to that purchased commercially (D/L‐Hpg) for the shortest prior synthesis. The metal‐free preparation from inexpensive aldehydes is also appealing.^[^ ^30^ ^]^ Moreover, more than half of the steps replaced hazardous coupling reagents servicing conventional amide synthesis with KI/UHP reagents for UmAS, resulting in a 50% reduction in mmol DEPBT/EDC per mmol feglymycin. The reduction is even more dramatic (88% decrease) by comparison to the feglymycin synthesis using microflow, where over 781 mmol DEPBT and trisphosgene were required for each mmol feglymycin. These measurements and comparison of length are intended to highlight the way in which the two amide synthesis paradigms dovetail superbly well.

In summary, we have completed a total synthesis of the antiviral feglymycin using a holistic approach to noncanonical peptide features that can be united through a blend of UmAS and conventional amide synthesis favoring the former.^[^ ^31^, ^32^, ^33^ ^]^ We further establish the utility of nitroalkanes as protected α‐amino acid equivalents made available through more sustainable synthesis, and readily applied to a complex target presenting diverse stereochemical arrays and epimerization‐prone residues. The success described here bodes well for its adaptation to other aryl glycinamide natural products (e.g., vancomycin, arylomycin, ramoplanin), and noncanonical residues known to be problematic using conventional amide synthesis.^[^ ^34^ ^]^

## Conflict of Interests

The authors declare no conflict of interest.

## Supporting information



Supporting Information

## Data Availability

The data that support the findings of this study are available in the Supporting Information of this article.
