# Supplementary material for: Total Synthesis of Feglymycin Using Umpolung Amide Synthesis
Source: Angew Chem Int Ed Engl. 2025 Jul 26;64(37):e202508819. doi: 10.1002/anie.202508819 (PMC12416467; doi:10.1002/anie.202508819)
Supplement: Supplementary file 1 — Supporting Information [file ANIE-64-e202508819-s001.pdf]

Total Synthesis of Feglymycin Using Umpolung Amide Synthesis

Preston C. Gourville, Jade A. Bing, Rashanique D. Quarels, Sergey V. Tsukanov, Kenneth E. Schwieter, Kazuyuki Tokumaru, Amanda B. Stephens, Dawn M. Makley, Bo Shen, Abigail N. Smith, Jeffrey N. Johnston*

Department of Chemistry and Vanderbilt Institute of Chemical Biology, Vanderbilt University, Nashville, Tennessee 37235

Table of Contents

Context for Aryl Glycine Epimerization During Condensative Amide Synthesis 3

General Experimental Procedures 4

tert-Butyl ((4-(benzyloxy)phenyl)(phenylsulfonyl)methyl)carbamate (1) 4

tert-Butyl (*E*)-(4-(benzyloxy)benzylidene)carbamate (2) 4

tert-Butyl ((1*S*)-1-(4-(benzyloxy)phenyl)-2-bromo-2-nitroethyl)carbamate (3) 5

tert-Butyl (*S*)-(1-(4-(benzyloxy)phenyl)-2-(methylamino)-2-oxoethyl)carbamate (4) 5

(*S*)-1-(4-(Benzyloxy)phenyl)-2-(methylamino)-2-oxoethan-1-aminium chloride (5) 6

tert-Butyl ((*R*)-2-(((*S*)-1-(4-(benzyloxy)phenyl)-2-(methylamino)-2-oxoethyl)amino)-1-(3,5-bis(benzyloxy)phenyl)-2-oxoethyl)carbamate (6) 6

tert-Butyl ((*R*)-2-(((*S*)-1-(4-(benzyloxy)phenyl)-2-(methyl(nitroso)amino)-2-oxoethyl)amino)-1-(3,5-bis(benzyloxy)phenyl)-2-oxoethyl)carbamate (7) 7

(*S*)-2-(4-(Benzyloxy)phenyl)-2-((*R*)-2-(3,5-bis(benzyloxy)phenyl)-2-((*tert*-butoxycarbonyl)amino)acetamido)acetic acid (8) 7

(*S*)-2-((*R*)-2-((*tert*-Butoxycarbonyl)amino)-2-(3,5-dihydroxyphenyl)acetamido)-2-(4-hydroxyphenyl)acetic acid (9) 7

Benzyl ((*R*)-2-(3,5-bis(benzyloxy)phenyl)-2-((*S*)-2-((*R*)-2-((*tert*-butoxycarbonyl)amino)-2-(3,5-dihydroxyphenyl)acetamido)-2-(4-hydroxyphenyl)acetamido)acetyl)-L-valinate (10) 8

Benzyl ((*R*)-2-((*S*)-2-((*R*)-2-amino-2-(3,5-dihydroxyphenyl)acetamido)-2-(4-hydroxyphenyl)acetamido)-2-(3,5-bis(benzyloxy)phenyl)acetyl)-L-valinate 2,2,2-trifluoroacetic acid (11) 9

Benzyl ((*R*)-2-(3,5-bis(benzyloxy)phenyl)-2-((*S*)-2-((*R*)-2-((*S*)-2-((*R*)-2-((*tert*-butoxycarbonyl)amino)-2-(3,5-dihydroxyphenyl)acetamido)-2-(4-hydroxyphenyl)acetamido)-2-(3,5-dihydroxyphenyl)acetamido)-2-(4-hydroxyphenyl)acetamido)acetyl)-L-valinate (12) 9

((*R*)-2-((*S*)-2-((*R*)-2-((*S*)-2-((*R*)-2-((*tert*-Butoxycarbonyl)amino)-2-(3,5-dihydroxyphenyl)acetamido)-2-(4-hydroxyphenyl)acetamido)-2-(3,5-dihydroxyphenyl)acetamido)-2-(4-hydroxyphenyl)acetamido)-2-(3,5-dihydroxyphenyl)acetyl)-L-valine (13) 10

Dibenzyl ((*S*)-2-((*R*)-2-((*S*)-2-((*R*)-2-((*S*)-2-((*R*)-2-((*S*)-2-((*R*)-2-((*tert*-butoxycarbonyl)amino)-2-(3,5-dihydroxyphenyl)acetamido)-2-(4-hydroxyphenyl)acetamido)-2-(3,5-dihydroxyphenyl)acetamido)-2-(4-hydroxyphenyl)acetamido)-2-(3,5-dihydroxyphenyl)acetamido)-3-methylbutanamido)-2-(3,5-dihydroxyphenyl)acetamido)-2-(4-hydroxyphenyl)acetyl)-L-phenylalanyl-L-aspartate (14) 11

Dibenzyl ((*S*)-2-((*R*)-2-((*S*)-2-((*R*)-2-((*S*)-2-((*R*)-2-((*S*)-2-((*R*)-2-amino-2-(3,5-dihydroxyphenyl)acetamido)-2-(4-hydroxyphenyl)acetamido)-2-(3,5-dihydroxyphenyl)acetamido)-2-(4-hydroxyphenyl)acetamido)-2-(3,5-dihydroxyphenyl)acetamido)-3-methylbutanamido)-2-(3,5-dihydroxyphenyl)acetamido)-2-(4-hydroxyphenyl)acetyl)-L-phenylalanyl-L-aspartate, 2,2,2-trifluoroacetic acid (15) 12

Dibenzyl ((*S*)-2-((*R*)-2-((*S*)-2-((*R*)-2-((*S*)-2-((*R*)-2-((*S*)-2-((*R*)-2-((*S*)-2-((*R*)-2-((*R*)-2-(((benzyloxy)carbonyl)amino)-2-(4-hydroxyphenyl)acetamido)-2-(3,5-dihydroxyphenyl)acetamido)-3-methylbutanamido)-2-(3,5-dihydroxyphenyl)acetamido)-2-(4-hydroxyphenyl)acetamido)-2-(3,5-dihydroxyphenyl)acetamido)-2-(4-hydroxyphenyl)acetamido)-2-(3,5-dihydroxyphenyl)acetamido)-3-methylbutanamido)-2-(3,5-dihydroxyphenyl)acetamido)-2-(4-hydroxyphenyl)acetyl)-L-phenylalanyl-L-aspartate (16) 12

Feglymycin (17) 13

tert-Butyl ((3,5-bis(benzyloxy)phenyl)(phenylsulfonyl)methyl)carbamate (18) 14

tert-Butyl (*E*)-(3,5-bis(benzyloxy)benzylidene)carbamate (19) 14

tert-Butyl ((1*R*)-1-(3,5-bis(benzyloxy)phenyl)-2-bromo-2-nitroethyl)carbamate (20) 14

Benzyl ((*R*)-2-(3,5-bis(benzyloxy)phenyl)-2-((tert-butoxycarbonyl)amino)acetyl)-L-valinate (21) 15

Benzyl ((*R*)-2-amino-2-(3,5-bis(benzyloxy)phenyl)acetyl)-L-valinate 2,2,2-trifluoroacetic acid (22) 15

Dibenzyl (*tert*-butoxycarbonyl)-L-phenylalanyl-L-aspartate (23) 16

Dibenzyl L-phenylalanyl-L-aspartate, 2,2,2-trifluoroacetic acid (24) 16

Dibenzyl ((*S*)-2-((*R*)-2-((*tert*-butoxycarbonyl)amino)-2-(3,5-dihydroxyphenyl)acetamido)-2-(4-hydroxyphenyl)acetyl)-L-phenylalanyl-L-aspartate (25) 16

Dibenzyl ((*S*)-2-((*R*)-2-amino-2-(3,5-dihydroxyphenyl)acetamido)-2-(4-hydroxyphenyl)acetyl)-L-phenylalanyl-L-aspartate, 2,2,2-trifluoroacetic acid (26) 17

((*R*)-2-((*R*)-2-((*tert*-Butoxycarbonyl)amino)-2-(4-hydroxyphenyl)acetamido)-2-(3,5-dihydroxyphenyl)acetyl)-L-valine (27) 17

((*R*)-2-((*R*)-2-((*tert*-Butoxycarbonyl)amino)-2-(4-hydroxyphenyl)acetamido)-2-(3,5-dihydroxyphenyl)acetyl)-L-valine (28) 18

((*R*)-2-((*R*)-2-(((Benzyloxy)carbonyl)amino)-2-(4-hydroxyphenyl)acetamido)-2-(3,5-dihydroxyphenyl)acetyl)-L-valine (30) 18

Context for Aryl Glycine Epimerization During Condensative Amide Synthesis

Listed below are selected literature examples of aryl glycine couplings using condensative amide synthesis, illustrating the varying degrees that epimerization occurs during the coupling step. Note that the degree to which epimerization occurs is not always quantified, and the inspection of these references for additional details is recommended.[1,2,3]

Commentary and details from prior syntheses related to aryl glycine epimerization:

Prior work: Süssmuth et al. (2009)

a) Dpg-Hpg Fragment Synthesis

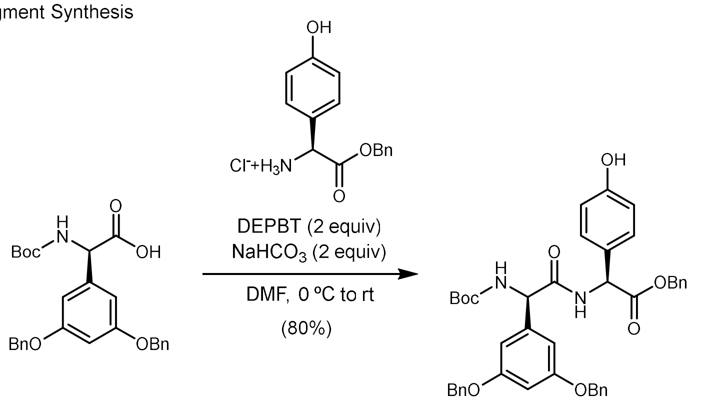

-undesired diastereomer detected (TLC) but not quantified

b) General comments:

"In particular, the coupling of the highly racemization prone amino acid Dpg in most cases led to the formation of large amounts of the diastereomeric product as a result of epimerization at the C$^{\alpha}$ position. Especially in more advanced stages of feglymycin synthesis, diastereomeric mixtures were not separable by standard chromatographic procedures (data not shown). Therefore, an iterative coupling of single amino acid building blocks was not possible."

Prior work: Fuse et al. (2016)

Optimization of aryl glycine couplings in flow chemistry

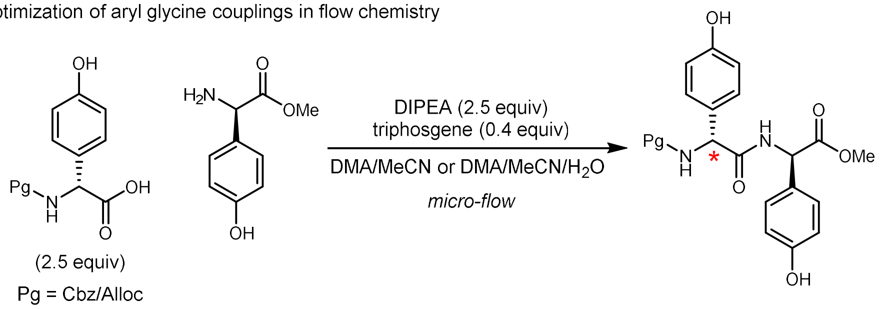

76-84% yield of desired dipeptide
1-4% yield of epimeric dipeptide

Prior work: Cryle and Tailhades et al. (2024)

SPPS preparation of aryl glycinamide-containing peptides

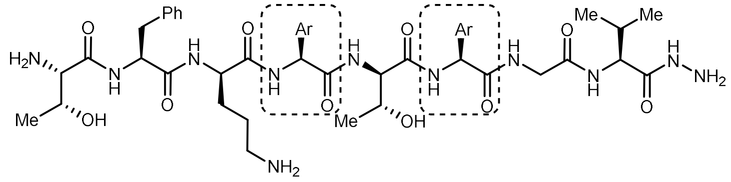

-across 12 examples, final peptides noted to be 83-98% diastereomer purity

General Experimental Procedures

All reagents and solvents were commercial grade and purified prior to use when necessary. Tetrahydrofuran (THF), dichloromethane (CH_2Cl_2), and toluene (PhMe) were dried by passage through a column of activated alumina as described by Grubbs.[4] Thin layer chromatography (TLC) was performed using glass-backed silica gel (250 µm) plates, and flash chromatography utilized 230–400 mesh silica gel from Sorbent Technologies. UV light, and/or the use of para-anisaldehyde, potassium permanganate, or phosphomolybdic acid with cerium(IV) sulfate solutions were used to visualize products. Melting points were measured on an SRS Meltemp melting point apparatus and were not corrected. IR spectra were recorded on a Nicolet IR 200 spectrophotometer and are reported in wavenumbers (cm^{-1}) analyzed as neat films on NaCl plates (transmission). Nuclear magnetic resonance spectra (NMR) were obtained on a Bruker DRX-400 (400 MHz), a Bruker AVIII-600 (600 MHz), or a Bruker AV-III 900 (900 MHz) spectrometer. Chemical shifts are measured to residual non-deuterated solvent as an internal standard. Preparative HPLC was performed on an Agilent 1260 system (column: Zorbax Eclipse XDB-C18; 21.2 mm x 150 mm, 5 µm for TFA and HCO_2H buffers or Agilent PrepHT XDB-C18; 21.1 X 100 mm, 5 µm for NH_4OAc buffer) with 210 nm monitoring wavelength and acetonitrile/water gradient and buffer as indicated. Mass spectra were recorded on a high resolution Thermo Electron Corporation MAT 95XP-Trap or Thermo Fisher LTQ Orbitrap XL by use of electro-spray ionization (ESI) or atmospheric pressure chemical ionization (APCI) by the Vanderbilt Mass Spectrometry Research Center (MSRC) Cores or the Indiana University Mass Spectrometry Facility. Optical rotations were measured on a Perkin Elmer-341 polarimeter or a Rudolph Research Analytical AUTOPOL III polarimeter. Chiral HPLC analysis was conducted on an Agilent 1100 series or an Agilent 1260 Infinity instrument using the designated ChiralPak column.

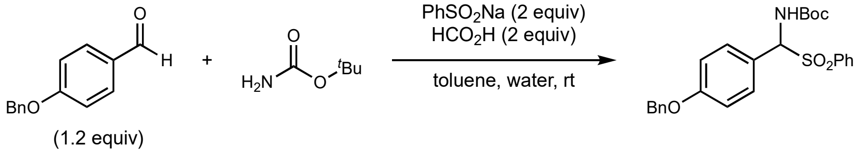

***tert*-Butyl ((4-(benzyloxy)phenyl)(phenylsulfonyl)methyl)carbamate (1).** To a solution of aldehyde (25.0 g, 117.8 mmol) in toluene (393 mL), was added *tert*-butyl carbamate (16.56 g, 141.3 mmol) and sodium benzene sulfinate (38.7 g, 235.6 mmol). Formic acid (8.3 mL, 240 mmol) was added dropwise followed by H_2O (116 mL) and the reaction was stirred for 7 days. Toluene was added, and the white precipitate was collected by vacuum filtration, triturating with toluene, hexanes, and water, collecting the filtrate. The solid was dissolved in dichloromethane, dried, and concentrated to provide the desired α-amidosulfone as a white amorphous solid (23.0 g), used without further purification.[5] The filtrate was concentrated to remove volatiles, then resuspended in toluene (393 mL) and stirred at rt for 7 days. Following the same purification, the desired α-amidosulfone was isolated (additional 7.0 g, 30.0 g total, 56%). Mp = 98-100 °C; R_f = 0.46 (30% EtOAc/hexanes); IR (film) 3341, 3057, 1695, 1601, 1508, 1309, 1225, 1198, 1161, 913, 742 cm^{-1}; ^1H NMR (600 MHz, $CDCl_3$) δ 7.91 (d, *J* = 7.3 Hz, 2H), 7.63 (ddd, *J* = 7.4, 7.4, 1.1 Hz, 1H), 7.52 (dd, *J* = 7.6, 7.4 Hz, 2H), 7.43-7.31 (m, 7H), 7.00 (d, *J* = 8.6 Hz, 2H), 5.93 (br s, 2H), 5.08 (s, 2H), 1.26 (s, 9H); ^{13}C NMR (150 MHz, $CDCl_3$) ppm 160.0, 153.6, 137.1, 136.6, 133.9, 130.4, 129.5, 129.1, 128.7, 128.1, 127.5, 122.1, 115.2, 81.1, 73.6, 70.1, 28.1; Exact mass calcd for $C_{19}H_{22}NO_3$ $[M-SO_2Ph]^+$ 312.1594, found 312.1561.

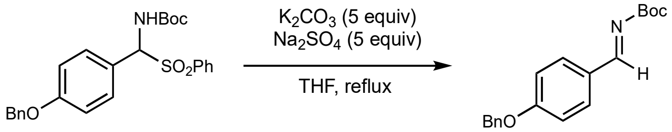

***tert*-Butyl (*E*)-(4-(benzyloxy)benzylidene)carbamate (2).** To a flame-dried 2-neck flask equipped with a stir bar and reflux condenser was added the *N*-Boc-α-amidophenylsulfone (20.0 g, 44.1 mmol), potassium carbonate (42.7 g, 308.7 mmol), and sodium sulfate (37.6 g, 264.6 mmol). Dry THF (221 mL) was added and the solution was heated at reflux until complete as indicated by ^1H NMR (~5 h). The reaction mixture was filtered through an oven-dried glass frit, concentrated, and carried

forward without further purification (13.7 g, quant.). ^{1}H NMR (400 MHz, $CDCl_3$) δ 8.89 (s, 1H), 7.89 (d, *J* = 8.8 Hz, 2H), 7.42-7.40 (m, 4H), 7.37-7.33 (m, 1H), 7.04 (d, *J* = 9.0 Hz, 2H), 5.13 (s, 2H), 1.59 (s, 9H).

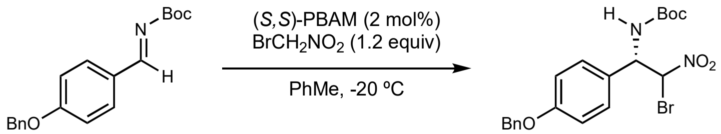

***tert*-Butyl ((1*S*)-1-(4-(benzyloxy)phenyl)-2-bromo-2-nitroethyl)carbamate (3).** In a flame-dried flask equipped with a stir bar, the *N*-Boc imine (13.7 g, 44.1 mmol) was dissolved in toluene (441 mL) and cooled to -40 °C. (*S,S*)-PBAM (447 mg, 882 μmol) was added and the reaction mixture was stirred for 10 minutes. Bromonitromethane (3.69 mL, 52.9 mmol) was added dropwise, and the reaction mixture was warmed to -20 °C, and stirred for 24 hours. The mixture was filtered through a plug of silica gel (EtOAc), and the filtrate was concentrated to afford the bromonitroalkane (18.3 g, 92%) as a 1:1.2 mixture of diastereomers (^{1}H NMR).[6] The diastereomers were determined to be 88%/90% ee by chiral HPLC analysis (Chiralpak AD-H, 20% ^{i}PrOH/hexanes, 1.0 mL/min, t_r(d_1e_1, major, minor) = 12.8 min, t_r(d_2e_2, minor, major) = 16.1 min, t_r(d_1e_2, major, major) = 17.9 min, t_r(d_2e_1, minor, minor) = 22.5 min. Recrystallization from EtOAc/hexanes afforded the bromonitroalkane as a 1:1.1 mixture of diastereomers with 98 and >99% ee (13.5 g, 68%). R_f = 0.38 (20% EtOAc/hexanes); mp = 144-146 °C; IR (film) 3361, 2979, 1691, 1564, 1510, 1354, 1249, 1163, 735 cm^{-1}; ^{1}H NMR (600 MHz, $CDCl_3$) δ 7.44-7.37 (m, 8H), 7.36-7.31 (m, 2H), 7.24 (d, *J* = 8.7 Hz, 2H), 7.21 (d, *J* = 8.7 Hz, 2H), 6.98 (d, *J* = 8.7 Hz, 2H), 6.97 (d, *J* = 8.7 Hz, 2H), 6.31 (br s, 1H), 6.27 (br s, 1H), 5.64 (br s, 1H), 5.58 (br dd, *J* = 8.7, 4.0 Hz, 1H), 5.41 (br s, 1H), 5.30 (br s, 1H), 5.06 (s, 2H), 5.05 (s, 2H), 1.45 (s, 9H), 1.44 (s, 9H); ^{13}C NMR (150 MHz, $CDCl_3$) ppm 159.5 (2C), 154.6, 154.3, 136.7 (4C), 128.8 (2C), 128.4 (2C), 128.3 (2C), 127.6 (2C), 115.5 (2C), 85.3, 82.2, 81.3, 81.2, 70.2 (2C), 57.9, 57.7, 28.4, 28.3; HRMS (ESI): Exact mass calcd for $C_{20}H_{23}BrN_2NaO_5$ $[M+Na]^+$ 473.0688, found 473.0681.

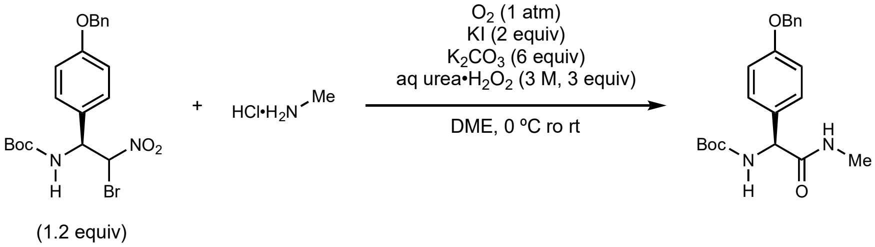

***tert*-Butyl (*S*)-(1-(4-(benzyloxy)phenyl)-2-(methylamino)-2-oxoethyl)carbamate (4)**. To a vigorously stirred mixture of amine (274.3 mg, 4.05 mmol), α-bromo nitroalkane (2.19 g, 4.86 mmol), potassium iodide[20] (1.34 g, 8.10 mmol), and potassium carbonate (3.36 g, 24.3 mmol)[21] in 1,2-dimethoxyethane (40.5 mL) under oxygen was added a freshly prepared solution of urea-hydrogen peroxide in water (3.0 M, 4.05 mL, 12.2 mmol) over 2 h by syringe pump at 0 °C.[22] After the addition was complete, the mixture was stirred for an additional 2 days at rt. Aq sodium thiosulfate was then added and the mixture was extracted with ethyl acetate. The organic layers were washed with 1 M aq HCl and brine, dried (sodium sulfate), and concentrated *in vacuo*. The crude mixture was purified by flash column chromatography (25-40% ethyl acetate in hexanes) to afford the product as a white solid (1.64 g, 67%). Mp = 147-151 °C; $[\alpha]_D^{20}$+77.3 (*c* 0.53, $CHCl_3$); R_f = 0.4 (40% EtOAc/ hexanes); IR (film) 3302, 2977, 1657, 1556, 1527, 1511, 1366, 1249, 1171 cm^{-1}; ^{1}H NMR (600 MHz, $CDCl_3$) δ 7.42-7.36 (m, 4H), 7.33-7.31 (m, 1H), 7.27 (d, *J* = 8.9 Hz, 2H), 6.93 (d, *J* = 8.7 Hz, 2H), 5.94 (s, 1H), 5.81 (br s, 1H), 5.10 (br s, 1H) 5.04 (s, 2H), 2.77 (d, *J* = 4.9 Hz, 3H), 1.26 (s, 9H); ^{13}C NMR (150 MHz, $CDCl_3$) ppm 171.1, 158.9, 155.4, 136.9,131.1,128.7, 128.6, 128.2, 127.6, 115.4, 80.1, 70.2, 58.0, 24.5, 26.6; HRMS (ESI): Exact mass calcd for $C_{21}H_{27}N_2O_4$ $[M+H]^+$ 371.1965, found 371.1967.

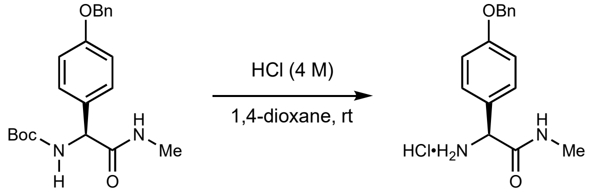

(*S*)-1-(4-(Benzyloxy)phenyl)-2-(methylamino)-2-oxoethan-1-aminium chloride (5). Peptide **4** (4.8 g, 13 mmol) was treated with HCl (4.0 M in 1,4-dioxane, 4 mL) and stirred at rt under argon. Upon complete deprotection (approx. 1-2 h), the volatiles were removed by vacuum (15 Torr). Diethyl ether was added to the flask and the mixture was concentrated to remove residual HCl. This was repeated twice and the resulting solid was concentrated *in vacuo* to yield the crude product as a white solid (4.0 g, quant.) which was carried forward without purification.

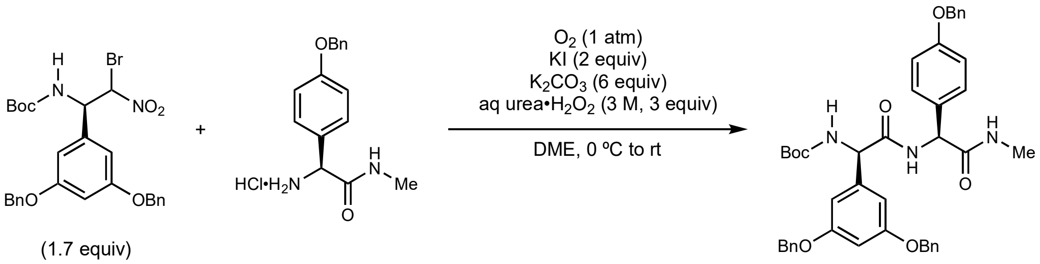

***tert*-Butyl ((*R*)-2-(((*S*)-1-(4-(benzyloxy)phenyl)-2-(methylamino)-2-oxoethyl)amino)-1-(3,5-bis(benzyloxy)phenyl)-2-oxoethyl)carbamate (6)**. To a vigorously stirred mixture of amine (2.871 g, 9.358 mmol), α-bromo nitroalkane (8.70 g, 15.6 mmol), potassium iodide[20] (3.107 g, 18.72 mmol), and potassium carbonate (7.760 g, 56.15 mmol)[21] in 1,2-dimethoxyethane (85 mL) was added a freshly prepared solution of urea-hydrogen peroxide in water (3.0 M, 9.4 mL, 28 mmol) over 2 h by syringe pump at 0 °C.[22] After the addition was complete, the mixture was stirred for an additional 2 days at rt. Aqueous sodium thiosulfate was then added and the mixture was extracted with ethyl acetate. The organic layers were washed with 1 M aq HCl and water. Due to the partial solubility of the product in ethyl acetate, the suspended solids were filtered off, yielding pure dipeptide. The organic layers were then dried (Na_2SO_4), concentrated, and then triturated with diethyl ether, then acetonitrile to yield the desired dipeptide as a white solid (4.4 g, 66%). Mp = 211-215 °C; $[\alpha]_D^{20}$ -12.2 (*c* 0.53, $CHCl_3$); R_f = 0.3 (60% EtOAc/hexanes); IR (film) 3297, 3034, 2980, 2361, 1685, 1641, 1606, 1513, 1164 cm^{-1}; ^1H NMR (600 MHz, DMSO-d_6) δ 8.78 (d, *J* = 7.9 Hz, 1H), 8.23 (d, *J* = 4.1 Hz, 1H), 7.42-7.30 (m, 16H), 7.21 (d, *J* = 8.6 Hz, 2H), 6.88 (d, *J* = 8.7 Hz, 2H), 6.76 (d, *J* = 1.9 Hz, 2H), 6.54 (dd, *J* = 1.9, 1.9 Hz, 1H), 5.34 (d, *J* =7.6 Hz, 2H), 4.99 (br s, 6H), 2.59 (d, *J* = 4.6 Hz, 3H), 1.40 (s, 9H); ^{13}C NMR (150 MHz, DMSO-d_6) ppm 170.2, 169.3, 159.3, 157.7, 154.9, 140.9, 137.0, 136.9, 130.9, 128.43, 128.41, 127.9, 127.8, 127.76, 127.5, 114.4, 106.4, 100.7, 78.5, 69.3, 69.1, 57.4, 55.5, 28.2, 25.6; HRMS (ESI): Exact mass calcd for $C_{43}H_{46}N_3O_7$ $[M+H]^+$ 716.3330, found 716.3335.

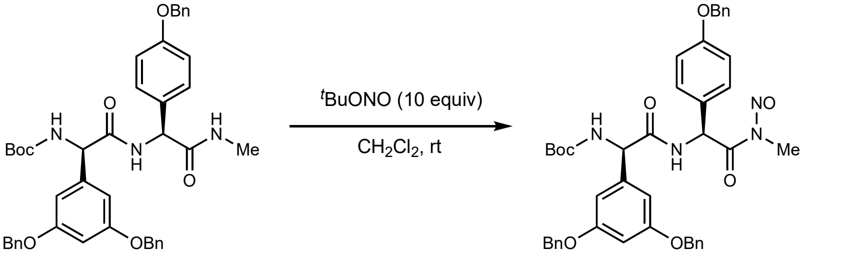

***tert*-Butyl ((*R*)-2-(((*S*)-1-(4-(benzyloxy)phenyl)-2-(methyl(nitroso)amino)-2-oxoethyl)amino)-1-(3,5-bis(benzyloxy)phenyl)-2-oxoethyl)carbamate (7)**. The dipeptide (1.3558 g, 1.8940 mmol) was suspended in CH_2Cl_2 (19 mL) at room temperature. *tert*-Butyl nitrite (2.37 mL, 18.940 mmol) was added in one portion and the consumption of starting amide was monitored by TLC.[7] Upon complete conversion of *N*-methyl amide the reaction becomes homogenous, the solution was then diluted with diethyl ether and concentrated in vacuo three times. All volatiles were removed by high vacuum, and the off-white solid (1.3848 g, 98%) was used immediately without further purification.

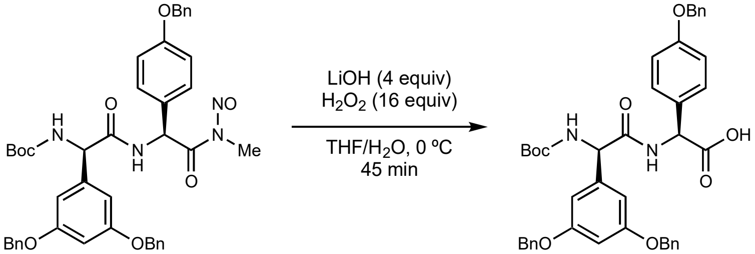

(*S*)-2-(4-(Benzyloxy)phenyl)-2-((*R*)-2-(3,5-bis(benzyloxy)phenyl)-2-((*tert*-butoxycarbonyl)amino)acetamido)acetic acid (8). The *N*-nitroso-*N*-methyl amide (1.3848 g, 1.8592 mmol) was dissolved in 3:1 THF/H_2O (37 mL) and cooled to 0 °C.[8] After 30 minutes at 0 °C, LiOH (178.1 mg, 7.437 mmol) and 30% H_2O_2 (3.04 mL, 29.7 mmol)[22] were added in succession. After 45 min, the reaction was quenched with sodium bisulfite at 0 °C and stirred for an additional 10 min. The reaction mixture was transferred to a separatory funnel and extracted with CH_2Cl_2. The aqueous layer was acidified to pH 3 and re-extracted with CH_2Cl_2 two times. The combined organic layers were washed with brine, dried (Na_2SO_4), filtered, and concentrated to yield an off-white solid (1.3014 g, quant.) that was used without further purification. Mp = 96-101°C; $[\alpha]_D^{20}$ -1.1 (*c* 0.53, $CHCl_3$); R_f = 0.3 (10% MeOH/CH_2Cl_2); IR (film) 3321, 3033, 2977, 2361, 1660, 1602, 1510, 1453, 1370, 1295, 1245, 1161 cm^{-1}; ^1H NMR (600 MHz, $CDCl_3$) δ 7.71 (br s, 1H), 7.38-7.26 (m, 15H), 7.24 (d, *J* = 8.5 Hz, 2H), 6.84 (d, *J* = 8.6 Hz, 2H), 6.55 (br s, 2H), 6.51 (br s, 1H), 5.97 (d, *J* = 8.9 Hz, 1H), 5.87 (br s, 1H), 5.58 (d, *J* = 6.3 Hz, 1H), 4.90-4.78 (m, 6H), 1.40 (s, 9H);[9] ^{13}C NMR (150 MHz, $CDCl_3$) ppm 172.9, 169.6, 160.4, 159.0, 156.3, 140.2, 136.9, 136.7, 128.7 (2C), 128.5, 128.2, 128.1, 127.8, 127.6, 115.1, 106.1, 102.2, 81.4, 70.2, 70.0, 56.9, 55.8, 28.4; HRMS (ESI): Exact mass calcd for $C_{42}H_{43}N_2O_8$ $[M+H]^+$ 703.3014, found 703.3018.

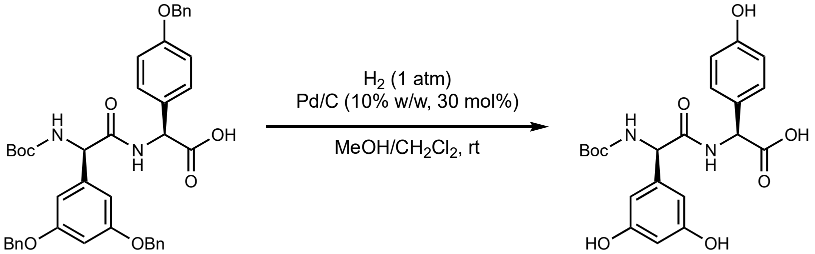

(*S*)-2-((*R*)-2-((*tert*-Butoxycarbonyl)amino)-2-(3,5-dihydroxyphenyl)acetamido)-2-(4-hydroxyphenyl)acetic acid (9). The acid (446.2 mg, 634.9 µmol) and palladium on carbon (202.7 mg, 10% w/w, 30 mol%)[10] were suspended in dichloromethane (6.4 mL) and methanol (6.4 mL) under argon. The atmosphere was evacuated (50 Torr) and refilled with argon three times. The reaction was evacuated (50 Torr) and refilled with hydrogen three times. The reaction was monitored by TLC, and upon completion (2 h), the hydrogen balloon was removed, the flask was purged with nitrogen, and the reaction mixture was filtered through a pad of Celite with methanol to afford the product as an off-white solid (274.5 mg, quant.). ^1H NMR (400 MHz, DMSO-d_6) δ 9.45 (br s, 1H), 9.15 (br s, 2H), 8.57 (br d, *J* = 6.0 Hz, 1H), 7.10 (d, *J* = 8.5 Hz, 2H), 7.01 (d, *J* = 8.9 Hz, 1H), 6.68 (d, *J* = 8.5 Hz, 2H), 6.24 (d, *J* = 2.0 Hz, 2H), 6.08 (dd, *J* = 2.0, 2.0 Hz, 1H), 5.16 (d, *J* = 8.9 Hz, 1H), 5.08 (d, *J* = 7.1 Hz, 1H), 1.38 (s, 9H); ^{13}C NMR (150 MHz, DMSO-d_6) ppm 172.0, 169.6, 158.1, 157.1, 154.6, 140.9, 128.6,

115.1, 105.2, 101.6, 78.4, 57.1, 56.0, 28.2; HRMS (ESI): Exact mass calcd for $C_{21}H_{25}N_2O_8$ $[M+H]^+$ 433.1605, found 433.1591. HRMS data were consistent with literature.[11,12]

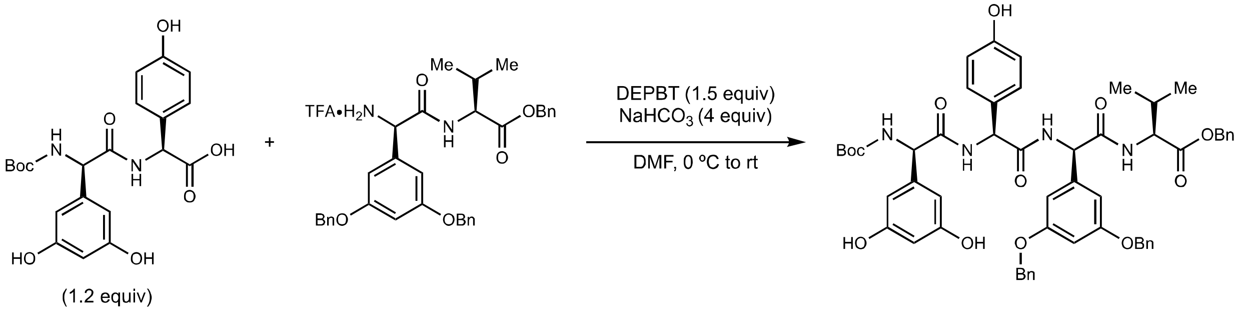

Benzyl ((*R*)-2-(3,5-bis(benzyloxy)phenyl)-2-((*S*)-2-((*R*)-2-((*tert*-butoxycarbonyl)amino)-2-(3,5-dihydroxyphenyl)acetamido)-2-(4-hydroxyphenyl)acetamido)acetyl)-L-valinate (10). To a solution of the amine (52.5 mg, 76.6 µmol) and the carboxylic acid (42.1 mg, 42.1 µmol) in DMF (0.39 mL) at 0 °C was added DEPBT (34.0 mg, 114 mmol) and sodium bicarbonate (25.7 mg, 306 µmol). The reaction mixture was stirred at 0 °C for 2 hours, then gradually warmed to room temperature. After 46 hours, the reaction was cooled to 0 °C and quenched by the addition of water. After stirring for 10 minutes, the mixture was diluted with ethyl acetate and the layers were separated. The aqueous layer was kept at 0 °C by addition of ice, and then extracted with ethyl acetate. The organic layers were combined, washed with ice water, satd aq $NaHCO_3$, 1 N aq HCl, and brine. The organic layer was dried, filtered, and concentrated. The crude mixture was purified by flash column chromatography (SiO_2, 10-55% (3:1 EtOAc:EtOH) in hexanes) to afford the product as a white solid (43.8 mg, 58%).[13] Mp = 152 °C (dec); $[\alpha]_D^{24}$ – 19.9 (*c* 0.92, DMSO); R_f = 0.59 (50% (3:1 EtOAc:EtOH) in hexanes); IR (film) 3301, 2967, 2927, 1638, 1514, 1378, 1165 cm^{-1}; ^1H NMR (600 MHz, DMSO-d_6) δ 9.36 (s, 1H), 9.18 (s, 2H), 9.02 (d, *J* = 8.2 Hz, 1H), 8.72 (d, *J* = 8.5 Hz, 1H), 8.61 (d, *J* = 7.7 Hz, 1H), 7.42-7.31 (m, 15H), 7.18 (d, *J* = 8.4 Hz, 2H), 6.96 (d, *J* = 8.7 Hz, 1H), 6.68 (m, 2H), 6.61 (d, *J* = 8.3 Hz, 2H), 6.49 (dd, *J* = 2.1, 2.1 Hz, 1H), 6.29 (d, *J* = 1.8 Hz, 2H), 6.10 (dd, *J* = 2.1, 2.1 Hz, 1H), 5.69-5.61 (m, 2H), 5.24 (d, *J* = 8.7 Hz, 1H), 5.20 (d, *J* = 12.4 Hz, 1H), 5.16 (d, *J* = 12.4 Hz, 1H), 4.97 (d, *J* = 11.8 Hz, 2H), 4.91 (d, *J* = 11.8 Hz, 2H), 4.22 (dd, *J* = 6.6, 8.3 Hz, 1H), 2.04 (qqd, *J* = 6.7, 6.7, 6.6 Hz, 1H), 1.38 (s, 9H), 0.78 (d, *J* = 6.7 Hz, 3H), 0.73 (d, *J* = 6.7 Hz, 3H); ^{13}C NMR (150 MHz, DMSO-d_6) δ 172.1, 171.3, 169.7, 169.5, 159.2, 158.1, 156.8, 154.6, 141.2, 141.0, 136.8, 135.8, 128.5 (2C), 128.2 (2C), 128.1, 127.9, 127.7, 114.8, 105.8, 105.3, 101.6, 100.9, 78.4, 69.2, 66.1, 57.4, 57.1, 55.6, 55.1, 30.3, 28.2, 18.8, 17.9; HRMS (ESI): Exact mass calcd for $C_{55}H_{59}N_4O_{12}$ $[M+H]^+$ 967.4124, found 967.4084.

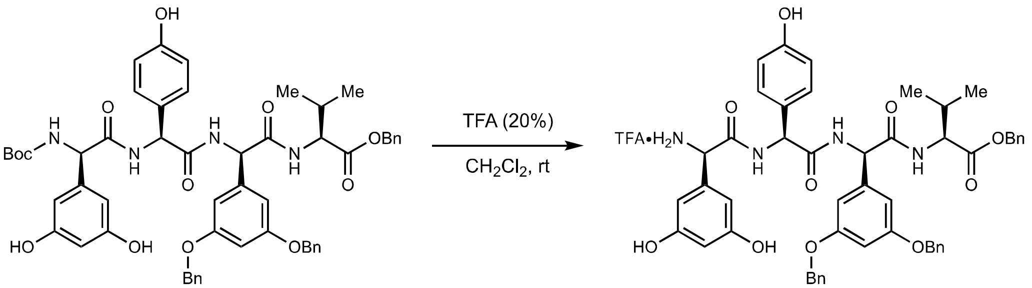

Benzyl ((*R*)-2-((*S*)-2-((*R*)-2-amino-2-(3,5-dihydroxyphenyl)acetamido)-2-(4-hydroxyphenyl)acetamido)-2-(3,5-bis(benzyloxy)phenyl)acetyl)-L-valinate 2,2,2-trifluoroacetic acid (11). The tetrapeptide (25.1 mg, 26.0 µmol) was dissolved in trifluoroacetic acid (0.05 mL) and dichloromethane (0.20 mL) and then stirred at rt under argon. After 5 h the volatiles were removed by vacuum (15 Torr). Diethyl ether was added to the flask and the mixture was concentrated to remove residual trifluoroacetic acid. This was repeated twice, and the resulting solid was concentrated *in vacuo* to yield the crude product as an off-white solid (25.3 mg, 99%). The material was carried forward without purification.

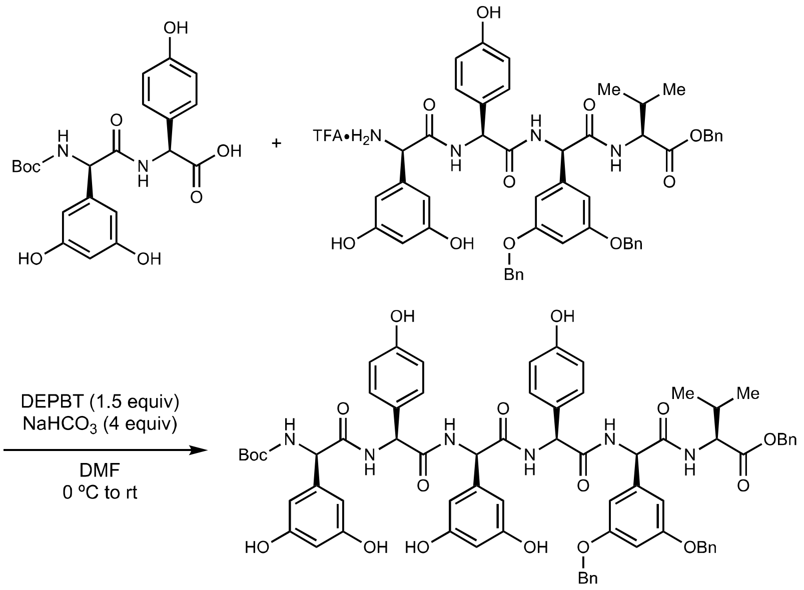

Benzyl ((*R*)-2-(3,5-bis(benzyloxy)phenyl)-2-((*S*)-2-((*R*)-2-((*S*)-2-((*R*)-2-((*tert*-butoxycarbonyl)amino)-2-(3,5-dihydroxyphenyl)acetamido)-2-(4-hydroxyphenyl)acetamido)-2-(3,5-dihydroxyphenyl)acetamido)-2-(4-hydroxyphenyl)acetamido)acetyl)-L-valinate (12). To a mixture of amine (25.3 mg, 25.8 µmol) and carboxylic acid (13.4 mg, 30.9 µmol) in DMF (0.13 mL) cooled to 0 °C[14] was added DEPBT (11.6 mg, 38.7 µmol) and $NaHCO_3$ (8.7 mg, 103 µmol). The mixture was stirred at 0 °C for 2 h, and then gradually warmed to room temperature. After 46 h, the reaction mixture was cooled to 0 °C and quenched with water. The reaction mixture was diluted with ethyl acetate, the layers were separated, and the aqueous layer was extracted with ethyl acetate. The organic layers were combined, and washed sequentially with ice water, satd aq sodium bicarbonate, 1 M aq HCl, and brine. The organic layer was dried and concentrated to a residue that was purified by flash column chromatography (SiO_2, 30-70% (3:1 EtOAc:EtOH) in hexanes) to yield an off-white solid (23.3 mg, 71%). Mp = 130 °C (dec); $[\alpha]_D^{24}$ – 12.4 (*c* 0.32, DMSO); R_f = 0.32 (50% (3:1 EtOAc:EtOH) in hexanes); IR (film) 3301, 2965, 2924, 1634, 1604, 1514, 1454, 1374, 1162 cm^{-1}; ^1H NMR (600 MHz, DMSO-d_6) δ 9.45-9.02 (m, 7H), 8.86-8.72 (m, 3H), 8.49 (d, *J* = 7.9 Hz, 1H), 7.40-7.35 (m, 12H), 7.35-7.30 (m, 3H), 7.17 (d, *J* = 8.4 Hz, 2H), 7.04 (d, *J* = 8.1 Hz, 2H), 7.00 (d, *J* = 8.2 Hz, 1H), 6.71-6.65 (m, 2H), 6.61-6.51 (m, 4H), 6.46 (dd, *J* = 1.9, 1.9 Hz, 1H), 6.29-6.26 (m, 2H), 6.22-6.18 (m, 2H), 6.09 (dd, *J* = 2.0, 2.0 Hz, 1H), 6.05 (dd, *J* = 2.0, 2.0 Hz, 1H), 5.71-5.66 (m, 2H), 5.62 (d, *J* = 7.3 Hz, 1H), 5.53 (d, *J* = 7.8 Hz, 1H), 5.21 (d, *J* = 8.3 Hz, 1H), 5.20 (d, *J* = 12.3 Hz, 1H), 5.15 (d, *J* = 12.3 Hz, 1H), 4.96 (d, *J* = 11.9 Hz, 2H), 4.89 (d, *J* = 11.9 Hz, 2H), 4.22 (dd, *J* = 7.9, 6.5 Hz, 1H), 2.07-1.97 (m, 1H), 1.36 (s, 9H), 0.76 (d, *J* = 6.7 Hz, 3H), 0.69 (d, *J* = 6.7 Hz, 3H); ^{13}C NMR (150 MHz, DMSO-d_6) ppm 171.4, 169.8, 169.4, 169.3, 169.1, 159.2, 158.1, 157.9, 156.7, 156.5, 141.3, 140.9, 140.3, 136.8, 135.7, 128.5, 128.4, 128.2 (2C), 128.1, 127.9, 127.8, 127.7, 114.7, 105.7, 105.4, 105.3, 101.6, 100.9, 78.3, 69.2, 66.2, 57.4, 57.2, 55.7, 55.3, 54.9, 54.8, 30.4, 28.1, 18.7, 17.8; HRMS (ESI): Exact mass calcd for $C_{71}H_{73}N_6O_{17}$ $[M+H]^+$ 1281.5027, found 1281.5040.

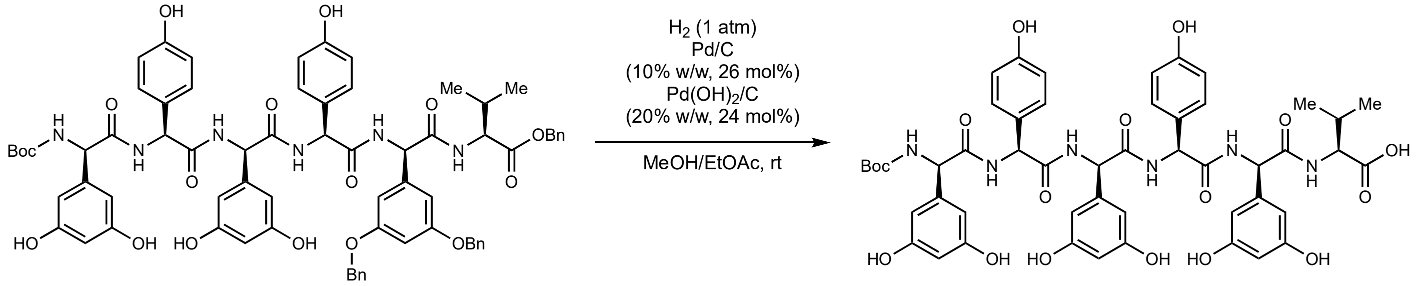

((*R*)-2-((*S*)-2-((*R*)-2-((*S*)-2-((*R*)-2-((*tert*-Butoxycarbonyl)amino)-2-(3,5-dihydroxyphenyl)acetamido)-2-(4-hydroxyphenyl)acetamido)-2-(3,5-dihydroxyphenyl)acetamido)-2-(4-hydroxyphenyl)acetamido)-2-(3,5-dihydroxyphenyl)acetyl)-L-valine (13). The hexapeptide (483.8 mg, 377.6 µmol), palladium on carbon (117.3 mg, 99.05 µmol, 10% w/w), and palladium hydroxide on carbon (63.4 mg, 90.3 µmol, 20% w/w) under argon was suspended in a minimal amount of ethyl acetate (0.20 mL). Methanol (3.8 mL) was added, and the mixture was stirred under argon until dissolved. The flask was evacuated (50 Torr) and backfilled with hydrogen (1 atm) three times. The mixture was stirred at rt for 2 days. The reaction mixture was filtered through Celite, and the pad was rinsed with MeOH. The volatiles were removed to afford the product as a brown solid (377.7 mg, 99%) which was carried forward without further purification. If desired, the hexapeptide could be further purified by preparatory HPLC according to the table below (retention time = 9.8 – 10.0 min). De-salting was achieved by size exclusion chromatography (Sephadex LH-20, MeOH), which afforded the product as a fluffy white solid after lyophilization.

Solvent A: 95:5 H_2O/MeCN (10 mM NH_4OAc), Solvent B: 95:5 MeCN/H_2O (10 mM NH_4OAc), flow rate: 20 mL/min

Time	%B	Time	%B
0.00	0.0	18.00	95.0
3.00	0.0	20.00	95.0
10.00	20.0	21.00	0.0
17.00	20.0	22.50	0.0

Mp = 224-234 °C (decomp); $[\alpha]_D^{20}$ -32.0 (*c* 0.50, MeOH); R_f = 0.1 (30% MeOH/DCM + 0.1% AcOH);[15] IR (neat) 3289, 1634, 1369, 1157, 1004, 681 cm^{-1}; ^{1}H NMR (600 MHz, DMSO-d_6)[16] δ 9.27 (br s, 3H), 9.10 (br s, 4H), 8.87 (d, *J* = 7.7 Hz, 1H), 8.80 (d, *J* = 7.7 Hz, 1H), 8.73(d, *J* = 8.0 Hz, 1H), 8.51 (d, *J* = 7.5 Hz, 1H), 8.34 (d, *J* = 8.3 Hz, 1H), 7.07 (d, *J* = 8.6 Hz, 2H), 7.06 (d, *J* = 9.1 Hz, 2H), 7.03 (d, *J* = 7.0 Hz, 1H), 6.57 (d, *J* = 6.9 Hz, 2H), 6.56 (d, *J* = 7.9 Hz, 2H), 6.28 (br s, 2H), 6.25 (br s, 2H), 6.22 (br s, 2H), 6.10 (br s, 1H), 6.07 (br s, 1H), 6.06 (br s, 1H), 5.67 (d, *J* = 8.2 Hz, 1H), 5.62 (d, *J* = 7.7 Hz, 1H), 5.53 (d, *J* = 7.8 Hz, 1H), 5.48 (d, *J* = 8.1 Hz, 1H), 5.21 (d, *J* = 8.3 Hz, 1H), 4.12 (dd, *J* = 8.3, 6.0 Hz, 1H), 2.00 (dqq, *J* = 13.0, 6.5, 6.5 Hz, 1H), 1.40 (s, 9H), 0.77 (d, *J* = 6.7 Hz, 3H), 0.73 (d, *J* = 6.7 Hz, 3H); ^{13}C NMR (150 MHz, DMSO-d_6) ppm 173.0, 169.9, 169.6, 169.5, 169.3, 169.1, 158.1, 158.0, 157.9, 156.55, 156.48, 154.6, 140.9, 140.7, 140.3, 128.9, 128.8, 127.9, 127.9, 114.7, 105.5, 105.4, 105.4, 101.6, 78.4, 57.3, 57.2, 55.8, 54.9, 54.8, 30.4, 28.2, 19.1; HRMS (ESI): Exact mass calcd for $C_{50}H_{54}N_6NaO_{17}$ $[M+Na]^+$ 1033.3438, found 1033.3439.

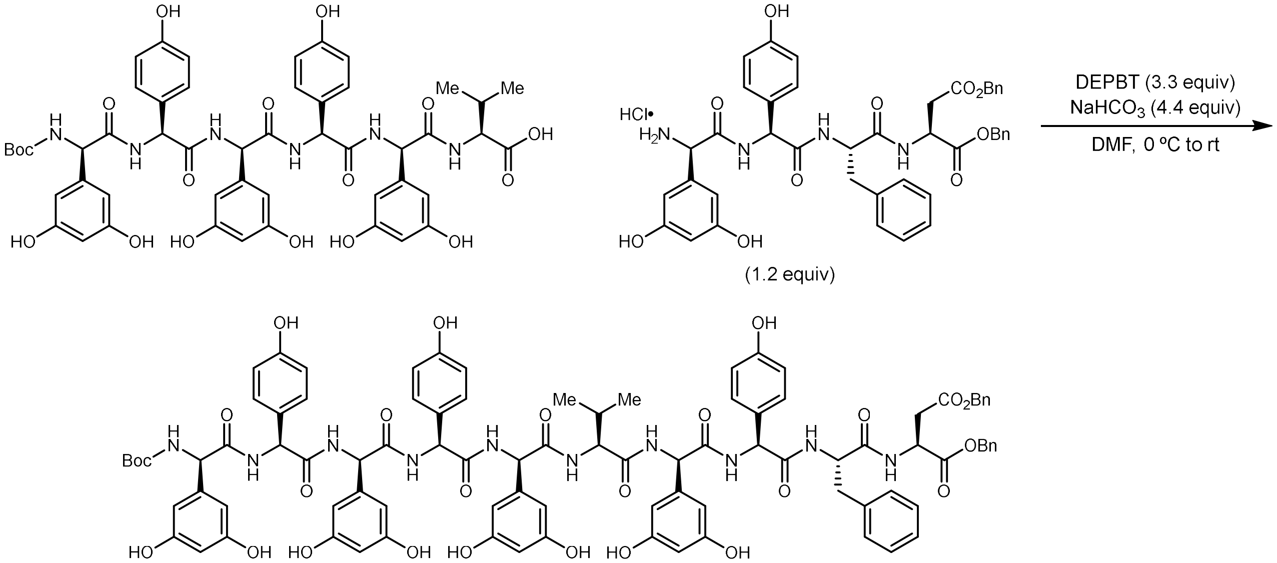

Dibenzyl ((*S*)-2-((*R*)-2-((*S*)-2-((*R*)-2-((*S*)-2-((*R*)-2-((*S*)-2-((*R*)-2-((*tert*-butoxycarbonyl)amino)-2-(3,5-dihydroxyphenyl)acetamido)-2-(4-hydroxyphenyl)acetamido)-2-(3,5-dihydroxyphenyl)acetamido)-2-(4-hydroxyphenyl)acetamido)-2-(3,5-dihydroxyphenyl)acetamido)-3-methylbutanamido)-2-(3,5-dihydroxyphenyl)acetamido)-2-(4-hydroxyphenyl)acetyl)-L-phenylalanyl-L-aspartate (14). In a flame-dried vial, the acid (26 mg, 30.9 µmol)[a] was dissolved in DMF (500 µL) and the solution was cooled to 0 °C. To the vial was added DEPBT (30.3 mg, 101 µmol) and $NaHCO_3$ (11.4 mg, 135 µmol), and the mixture was stirred for 3 hours. The amine·HCl salt (32.5 mg, 0.037 µmol)[b] was added as a solid, and DMF (175 µL) was used to transfer any residual amine from the vial. The reaction mixture stirred at 0 °C for 2 hours and then at room temperature for 48 hours. The reaction was quenched with a few drops of water, and the mixture was concentrated. The crude material was purified by preparative HPLC (retention time = 15.1 min) according to the table below to afford the product as a cream-colored solid (22.7 mg, 38%).[17] Mp >275 °C; $[\alpha]_D^{20}$ -20 (*c* 0.82, MeOH); R_f = 0.07 (20% MeOH/DCM); IR (film) 3294, 2965, 2922, 1731, 1645, 1515, 1159 cm^{-1}; ^1H NMR (600 MHz, DMSO-d_6) δ 9.29-9.10 (series of br s, 10H)[18], 8.84 (d, *J* = 7.9 Hz, 1H), 8.72 (d, *J* = 6.3 Hz, 1H), 8.68 (d, *J* = 7.3 Hz, 1H), 8.56 (d, *J* = 8.0 Hz, 1H), 8.53-8.47 (m, 3H), 8.41 (d, *J* = 8.2 Hz, 1H), 8.17 (d, *J* = 6.7 Hz, 1H), 7.35-7.29 (m, 12H), 7.23-7.16 (series of m, 5H), 7.07 (appt dd, *J* = 7.9, 7.9 Hz, 3H), 7.08 (d, *J* = 8.1 Hz, 1H), 6.97-6.94 (m, 2H), 6.57-6.55 (m, 6H), 6.29 (dd, *J* = 13.2, 1.7 Hz, 4H), 6.22-6.21 (m, 3H), 6.10-6.09 (m, 2H), 6.06 (d, *J* = 1.8 Hz, 2H), 5.63 (d, *J* = 7.7 Hz, 2H), 5.54 (d, *J* = 8.1 Hz, 1H), 5.49 (d, *J* = 7.9 Hz, 1H), 5.45 (d, *J* = 7.2 Hz, 1H), 5.32 (d, *J* = 7.5 Hz, 1H), 5.21 (d, *J* = 8.4 Hz, 1H), 5.09-5.01 (m, 4H), 4.73-4.69 (m, 1H), 4.58-4.51 (m, 1H), 4.38 (br dd, *J* = 8.4, 6.1 Hz, 1H), 2.99-2.97 (m, 1H), 2.86-2.71 (series of m, 3H), 1.88 (qqd, *J* = 6.6, 6.6, 6.1 Hz, 1H), 1.38 (s, 9H), 0.62 (d, *J* = 6.5 Hz, 3H), 0.59 (d, *J* = 6.5 Hz, 3H); ^{13}C NMR (150 MHz, DMSO-d_6) ppm 171.0 (2C), 170.7, 170.4, 169.9 (2C), 169.8, 169.7, 169.6, 169.4, 169.2 (2C), 158.3, 158.2 (2C), 158.1, 156.8, 156.7 (2C), 154.8, 141.1 (2C), 140.7, 140.6, 137.5, 135.9, 135.8, 129.4, 129.0, 128.8, 128.6 (2C), 128.4 (2C), 128.3 (2C), 128.1, 128.0, 126.6, 115.0, 105.8, 105.6, 105.5 (2C), 101.9, 101.8, 78.6, 66.6, 66.1, 57.4, 57.2, 56.3, 56.0, 55.9, 55.7, 55.1 (2C), 53.9, 48.7, 37.7, 35.8, 31.7, 28.3, 19.2, 17.7; HRMS (ESI): Exact mass calcd for $C_{93}H_{93}N_{10}O_{26}$ $[M-H]^-$ 1765.6268, found 1765.6323.

Solvent A: H_2O (0.1% HCOOH), Solvent B: MeCN (0.1% HCOOH), flow rate: 20 mL/min

Time	%B	Time	%B
0.00	0.0	23.00	95.0
2.00	20.0	23.50	20.0
20.00	75.0		

[a] This specific preparation used HPLC-purified carboxylic acid.

[b] This specific preparation used an HCl-amine salt, however, comparable yields were obtained with TFA-amine salts.

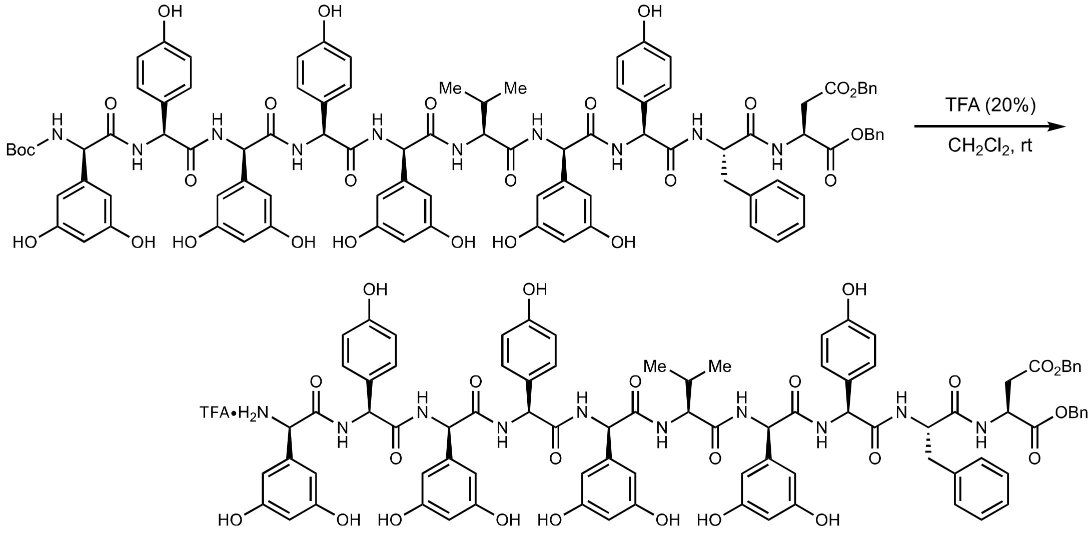

Dibenzyl ((*S*)-2-((*R*)-2-((*S*)-2-((*R*)-2-((*S*)-2-((*R*)-2-((*S*)-2-((*R*)-2-amino-2-(3,5-dihydroxyphenyl)acetamido)-2-(4-hydroxyphenyl)acetamido)-2-(3,5-dihydroxyphenyl)acetamido)-2-(4-hydroxyphenyl)acetamido)-2-(3,5-dihydroxyphenyl)acetamido)-3-methylbutanamido)-2-(3,5-dihydroxyphenyl)acetamido)-2-(4-hydroxyphenyl)acetyl)-L-phenylalanyl-L-aspartate, 2,2,2-trifluoroacetic acid (15). The decapeptide (24.7 mg, 14.0 μmol) was dissolved in trifluoroacetic acid (0.20 mL) and dichloromethane (0.80 mL), and then stirred at rt under argon. After 16 h, the volatiles were removed by vacuum (15 Torr). Diethyl ether was added to the flask and the mixture was concentrated to remove residual trifluoroacetic acid. This was repeated twice and the resulting solid was concentrated *in vacuo* to yield the crude product as an off-white solid (22.5 mg, 90%) which was carried forward without purification.

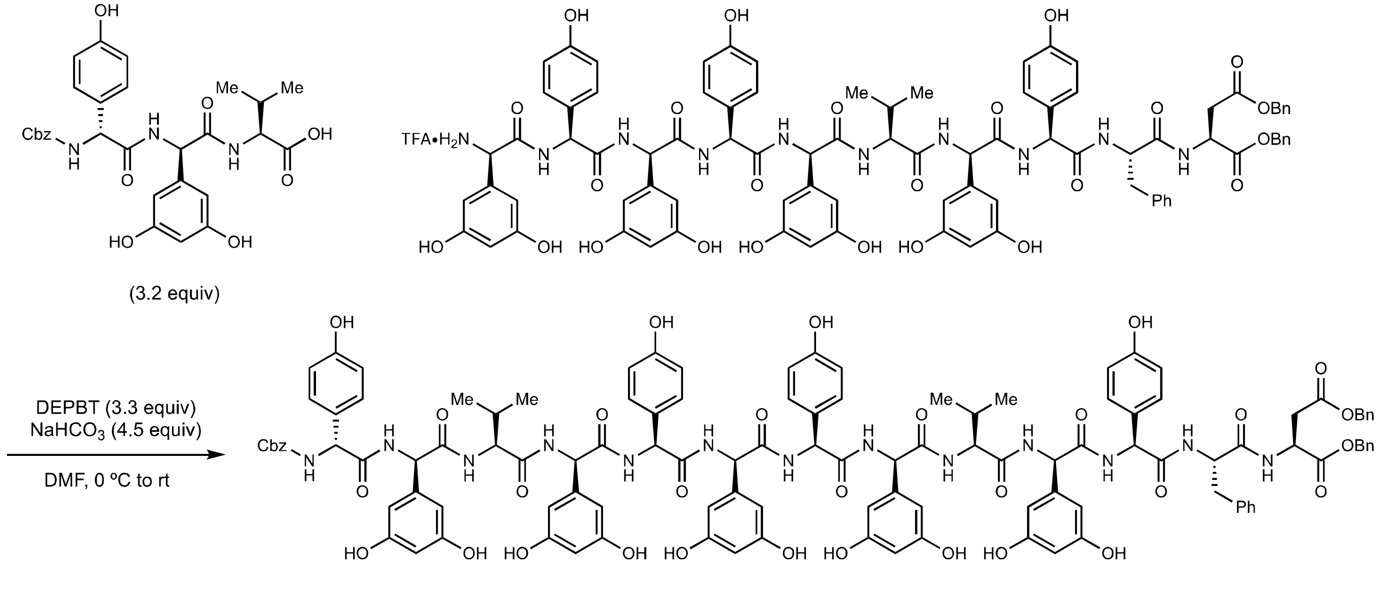

Dibenzyl ((*S*)-2-((*R*)-2-((*S*)-2-((*R*)-2-((*S*)-2-((*R*)-2-((*S*)-2-((*R*)-2-((*S*)-2-((*R*)-2-((*R*)-2-(((benzyloxy)carbonyl)amino)-2-(4-hydroxyphenyl)acetamido)-2-(3,5-dihydroxyphenyl)acetamido)-3-methylbutanamido)-2-(3,5-dihydroxyphenyl)acetamido)-2-(4-hydroxyphenyl)acetamido)-2-(3,5-dihydroxyphenyl)acetamido)-2-(4-hydroxyphenyl)acetamido)-2-(3,5-dihydroxyphenyl)acetamido)-3-methylbutanamido)-2-(3,5-dihydroxyphenyl)acetamido)-2-(4-hydroxyphenyl)acetyl)-L-phenylalanyl-L-aspartate (16). To a solution of the amine

(22.5 mg, 12.6 µmol) and carboxylic acid (22.9 mg, 40.5 µmol) in DMF (0.21 mL) at 0 °C was added DEPBT (12.3 mg, 41.1 µmol) and sodium bicarbonate (4.8 mg, 57 µmol). The reaction was stirred at 0 °C for 2 h, then was gradually warmed to room temperature. After 46 h, the reaction was cooled to 0 °C and quenched with water. After stirring for 10 min, the reaction mixture was diluted with methanol and concentrated. Size exclusion chromatography (Sephadex LH-20, MeOH) yielded the desired tridecapeptide as a white solid (18.4 mg, 66%).[17] ^{1}H NMR (600 MHz, DMSO-d_6) δ 9.55-9.06 (br m, 14H), 8.78 (d, J = 7.3 Hz, 1H), 8.69 (d, J = 7.7 Hz, 1H), 8.67 (d, J = 8.1 Hz, 1H), 8.62 (d, J = 6.7 Hz, 1H), 8.59-8.43 (m, 5H), 8.41 (d, J = 8.5 Hz, 1H), 8.11 (d, J = 7.5 Hz, 1H), 7.88 (d, J = 8.4 Hz, 1H), 7.85 (d, J = 8.5 Hz, 1H), 7.36-7.27 (m, 15H), 7.24-7.12 (m, 7H), 7.06 (d, J = 8.5 Hz, 2H), 7.03 (d, J = 8.6 Hz, 2H), 6.93 (d, J = 8.4 Hz, 2H), 6.68 (d, J = 8.5 Hz, 2H), 6.59-6.51 (m, 6H), 6.35 (d, J = 1.8 Hz, 2H), 6.31 (d, J = 1.7 Hz, 2H), 6.29 (d, J = 1.7 Hz, 2H), 6.22 (d, J = 1.8 Hz, 2H), 6.19 (d, J = 1.9 Hz, 2H), 6.12 (dd, J = 2.0, 2.0 Hz, 1H), 6.11 (dd, J = 2.1, 2.1 Hz, 1H), 6.09 (dd, J = 2.0, 2.0, 1H), 6.07 (dd, J = 2.0, 2.0 Hz, 1H), 6.06 (dd, J = 2.0, 2.0 Hz, 1H), 5.64 (d, J = 7.9 Hz, 1H), 5.59 (d, J = 7.8 Hz, 1H), 5.57 (d, J = 7.7 Hz, 1H), 5.52 (d, J = 7.8 Hz, 1H), 5.46 (d, J = 7.5 Hz, 1H), 5.40 (d, J = 7.2 Hz, 2H), 5.33-5.27 (m, 2H), 5.10-4.97 (m, 6H), 4.72-4.67 (m, 1H), 4.58-4.51 (m, 1H), 4.43-4.31 (m, 2H), 3.02-2.94 (m, 1H), 2.87-2.69 (m, 3H), 1.92-1.81 (m, 2H), 0.61 (d, J = 6.6 Hz, 3H), 0.59 (d, J = 6.8 Hz, 3H), 0.57 (d, J = 6.8 Hz, 3H), 0.54 (d, J = 6.6 Hz, 3H); ^{13}C NMR (150 MHz, DMSO-d_6) ppm 170.8 (2C), 170.4, 170.3, 169.8, 169.7 (2C), 169.6, 169.5 (2C), 169.4, 169.2, 169.1 (2C), 169.0, 158.1 (3C), 158.0 (3C), 156.9, 156.7, 156.6, 156.5, 140.9 (2C), 140.8, 140.6 (2C), 137.4, 136.9, 135.8, 135.6, 129.2 (2C), 128.9, 128.5 (2C), 128.4 (3C), 128.3, 128.2, 128.1 (3C), 128.0 (2C), 127.8 (2C), 127.7, 126.4, 115.0 (2C), 114.8 (6C), 105.7, 105.6 (3C), 105.5, 105.4 (3C), 105.3, 105.2, 101.8 (2C), 101.7 (2C), 101.6, 66.4, 66.0, 65.6, 57.4, 57.0, 56.8, 56.1 (2C), 55.8, 55.7, 55.6, 55.5, 55.0, 54.9, 53.7, 48.5, 37.6, 35.7, 31.5, 31.4, 19.1, 19.0, 17.6, 17.2; HRMS (ESI): Exact mass calcd for $C_{117}H_{119}N_{14}O_{32}$ [M+NH$_4$]$^+$ 2231.8109, found 2231.8080.

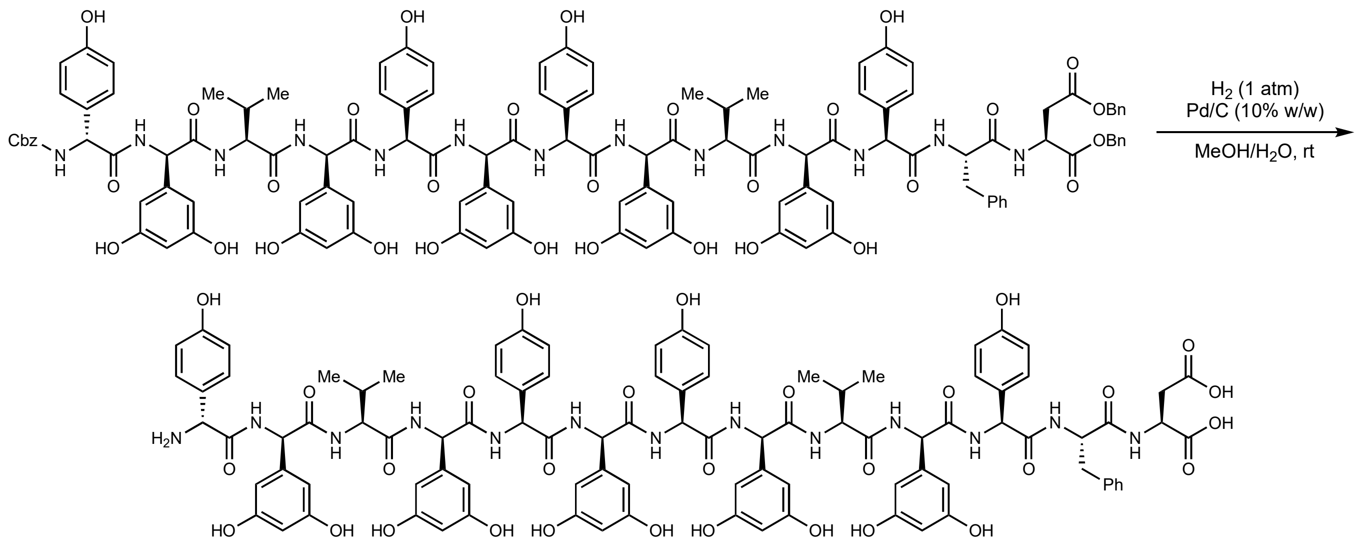

Feglymycin (17). To a flask containing tridecapeptide (47.1 mg, 21.3 µmol) and palladium on carbon (10% w/w, 57.4 mg) under Argon was added methanol (2.5 mL). The flask was evacuated (50 Torr) and refilled with hydrogen, cycling five times. After 3 h, water (0.05 mL) was added to the reaction mixture and the flask was evacuated (50 Torr) and refilled with hydrogen, cycling five times. After 16 h, the reaction flask was evacuated (50 Torr) and refilled with argon, cycling three times. The reaction mixture was filtered through Celite with methanol, and then concentrated to yield a nearly pure off-white solid (26.8 mg, 66%). The sample for characterization was further purified *via* reverse-phase preparatory HPLC (t_R = 11.7 min, see table for gradient). $[\alpha]_D^{23}$ – 47.9 (c 0.11, MeOH); ^{1}H NMR (900 MHz, DMSO-d_6) δ 12.72 (br s, 1H), 12.41 (br s, 1H), 9.73 (s, 1H), 9.31 (s, 1H), 9.29 (s, 1H), 9.27 (s, 1H), 9.22 (s, 2H), 9.15 (s, 2H), 9.13 (s, 2H), 9.09 (s, 2H), 9.08 (s, 2H), 8.92 (d, J = 7.8 Hz, 1H), 8.77 (d, J = 7.4 Hz, 1H), 8.67 (d, J = 7.8 Hz, 1H), 8.64 (d, J = 8.6 Hz, 1H), 8.62 (d, J = 7.8 Hz, 1H), 8.51 (d, J = 7.2 Hz, 2H), 8.47 (d, J = 7.3 Hz, 1H), 8.42 (br s, 2H), 8.35 (d, J = 8.1 Hz, 1H), 8.27 (br s, 1H), 8.11 (d, J = 8.6 Hz, 1H), 7.95 (d, J = 9.0 Hz, 1H), 7.32 (d, J = 8.5 Hz, 2H), 7.26-7.20 (m, 4H), 7.17-7.13 (m, 1H), 7.06 (d, J = 8.4 Hz, 2H), 7.05 (d, J = 8.4 Hz, 2H), 6.93 (d, J = 8.6 Hz, 2H), 6.81 (d, J = 8.6 Hz, 2H), 6.60-6.50 (m, 6H), 6.36 (d, J = 2.0 Hz, 2H), 6.29 (d, J = 1.9 Hz, 2H), 6.25 (d, J = 1.9 Hz, 2H), 6.20 (d, J = 1.8 Hz, 2H), 6.17 (d, J = 1.8 Hz, 2H), 6.13 (dd, J = 1.8, 1.8 Hz, 1H), 6.09 (dd, J = 1.8, 1.8 Hz, 1H),

6.07 (dd, *J* = 1.9, 1.9 Hz, 1H), 6.06 (dd, *J* = 2.0, 2.0 Hz, 2H), 5.61 (d, *J* = 8.1 Hz, 1H), 5.59 (d, *J* = 8.0 Hz, 1H), 5.53 (d, *J* = 7.7 Hz, 1H), 5.51 (d, *J* = 7.2 Hz, 1H), 5.49 (d, *J* = 7.3 Hz, 1H), 5.46 (d, *J* = 7.7 Hz, 1H), 5.40 (d, *J* = 7.6 Hz, 1H), 5.31 (d, *J* = 7.6 Hz, 1H), 4.99 (br s, 1H), 4.56-4.50 (m, 1H), 4.47 (br s, 1H), 4.39-4.33 (m, 2H), 3.04 (d, *J* = 13.8, 4.2 Hz, 1H), 2.81 (dd, *J* = 13.5, 8.9 Hz, 1H), 2.61 (br s, 1H), 1.93-1.82 (m, 2H), 0.61 (d, *J* = 6.8 Hz, 3H), 0.59 (d, *J* = 6.8 Hz, 3H), 0.55 (d, *J* = 6.8 Hz, 3H), 0.53 (d, *J* = 6.8 Hz, 3H); ^{13}C NMR (226 MHz, DMSO-*d*$_6$) ppm 170.3, 170.2, 169.7, 169.6, 169.5, 169.4, 169.0 (2C), 158.1, 158.0, 157.9, 156.6, 156.5 (2C), 141.0, 140.9, 140.8, 140.7, 140.6, 137.5, 129.4, 129.3, 128.6 (2C), 128.2, 128.1 (2C), 128.0, 126.3, 115.5, 114.8 (2C), 114.7, 105.6 (2C), 105.4 (2C), 105.3, 101.8, 101.7, 101.6, 57.0, 56.7, 56.3, 56.0, 55.8, 55.7 (2C), 55.4, 55.1, 54.9, 53.8, 48.6, 40.0, 39.9, 31.4, 19.0 (2C), 17.5, 17.1; HRMS (ESI): Exact mass calcd for $C_{95}H_{98}N_{13}O_{30}$ [M+H]$^+$ 1900.6537, found 1900.6533.

Solvent A: H_2O (0.1% TFA), Solvent B: MeCN (0.1% TFA), flow rate: 15 mL/min

Time	%B	Time	%B
0.00	15.0	25.00	95.0
15.00	30.0	30.00	95.0

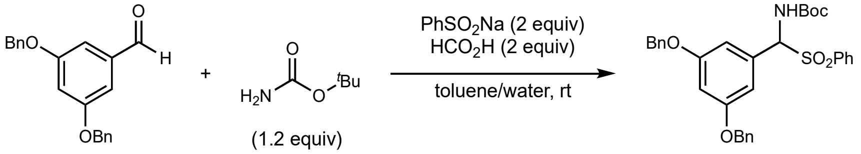

***tert*-Butyl ((3,5-bis(benzyloxy)phenyl)(phenylsulfonyl)methyl)carbamate (18).** To a solution of aldehyde (23.8 g, 74.8 mmol) in toluene (250 mL), was added *tert*-butyl carbamate (10.5 g, 89.7 mmol) and sodium benzene sulfinate (24.6 g, 150 mmol). Formic acid (5.3 mL, 140 mmol) was added dropwise followed by H_2O (77 mL) and the reaction was stirred for 8 days. Toluene was added, and the white precipitate was collected by vacuum filtration, washing with toluene to provide the desired α-amidosulfone as a white amorphous solid (37.3 g, 89%) without further purification. Mp = 158 °C (dec); R$_f$ = 0.53 (30% EtOAc/ hexanes); IR (film) 3350, 3063, 3034, 2979, 2932, 2871, 1705, 1598, 1519, 1448, 1308, 1164, 1145, 1049, 736, 687 cm^{-1}; (400 MHz, $CDCl_3$) δ 7.92 (d, *J* = 7.6 Hz, 2H), 7.64 (dd, *J* = 7.5, 7.4 Hz, 1H), 7.53 (dd, *J* = 7.7, 7.6 Hz, 2H), 7.46-7.30 (m, 10H), 6.71 (br s, 2H), 6.65 (dd, *J* = 2.1, 2.1 Hz, 1H), 5.88 (d, *J* = 10.7 Hz, 1H), 5.81 (d, *J* = 10.7 Hz, 1H), 4.99 (s, 4H), 1.27 (s, 9H); ^{13}C NMR (100 MHz, $CDCl_3$) ppm 160.2, 153.6, 137.1, 136.6, 134.1, 132.2, 129.6, 129.2, 128.7, 128.2, 127.7, 108.3, 103.6, 81.3, 74.1, 70.4, 28.2; HRMS (ESI): Exact mass calcd for $C_{32}H_{37}N_2O_6S^+$ [M+NH$_4$]$^+$ 577.2367, found 577.2365.

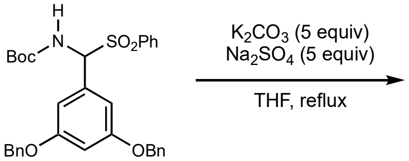

***tert*-Butyl (*E*)-(3,5-bis(benzyloxy)benzylidene)carbamate (19).** To a flame-dried 2-neck flask equipped with a stir bar and reflux condenser was added *N*-Boc-α-amidophenylsulfone (25.0 g, 44.7 mmol), potassium carbonate (30.9 g, 223 mmol), and sodium sulfate (31.7 g, 223 mmol). Dry THF (225 mL) was added and the solution was heated at reflux until complete (^{1}H NMR, ~5 h). The reaction mixture was filtered through an oven-dried glass frit, concentrated, and carried forward without further purification. ^{1}H NMR (400 MHz, $CDCl_3$) δ 8.76 (s, 1H), 7.39-7.32 (m, 10H), 7.16 (d, *J* = 2.3 Hz, 2H), 6.81 (t, *J* = 2.3 Hz, 1H), 5.07 (s, 4H), 1.59 (s, 9H).

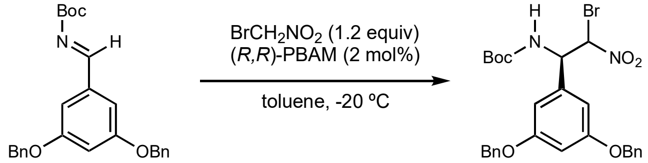

***tert*-Butyl ((1*R*)-1-(3,5-bis(benzyloxy)phenyl)-2-bromo-2-nitroethyl)carbamate (20).** In a flame-dried flask equipped with a stir bar, the *N*-Boc imine (18.7 g, 44.7 mmol) was dissolved in toluene (450 mL) and cooled to -40 °C. (*R,R*)-PBAM (453

mg, 894 µmol) was added and the reaction mixture was stirred for 10 minutes. Bromonitromethane (4.7 mL, 67 mmol) was added dropwise, the reaction mixture was warmed to -20 °C, and stirred for 24 hours. Upon completion of the reaction, the mixture was filtered through a plug of silica gel (EtOAc), and concentrated to afford the bromonitroalkane (24.2 g, 97%) as a 1:1.1 mixture of diastereomers (^{1}H NMR).[19] The diastereomers were determined to be 92%/92% ee by chiral HPLC analysis (Chiralpak OD-H, 5% EtOH/hexanes, 1.0 mL/min, t_r(d_1e_1, major, minor) = 18.0 min, t_r(d_2e_1, minor, major) = 20.4 min, t_r(d_2e_2, minor, major) = 22.6 min, t_r(d_1e_2, major, major) = 26.9 min. Mp = 161.0–164.0 °C; R_f = 0.54 (20% EtOAc/hexanes); IR (film) 3362, 2983, 2357, 1686, 1561, 1164 cm^1; 1:1.6 mixture of diastereomers: ^{1}H NMR (400 MHz, DMSO-d_6) δ 7.93 (d, *J* = 9.6 Hz, 1H), 7.79 (d, *J* = 10.0 Hz, 1H), 7.45–7.30 (m, 20 H), 6.77 (s, 2H), 6.71 (d, *J* = 2.0 Hz, 2H), 6.65 (d, *J* = 10.0 Hz, 1H), 6.64 (d, *J* = 2.0 Hz, 2H), 6.52 (d, *J* = 9.6, 1H), 5.35 (dd, *J* = 9.6, 9.0 Hz, 1H), 5.25 (dd, *J* = 9.6, 9.4 Hz, 1H), 5.07 (s, 4H), 5.05 (s, 4H), 1.38 (s, 9H), 1.33 (s, 9H); ^{13}C NMR (100 MHz, DMSO-d_6) ppm 159.6 (2C), 159.5 (2C), 154.7, 154.2, 138.3, 138.2, 136.8, 136.7, 128.4 (8C), 127.9 (12C), 107.3 (2C), 106.8 (2C), 101.7 (2C), 83.7, 80.7, 79.1 (2C), 69.5 (4C), 58.6, 58.0, 28.1, 28.0; HRMS (ESI): Exact mass calcd for $C_{27}H_{29}N_2O_6Na$ [M+Na]$^+$ 579.1136, found 579.1107.

Recrystallization from EtOAc/hexanes afforded the bromonitroalkane as a 1:1.1 mixture of diastereomers with 98 and 99% ee (18.9 g, 76%).

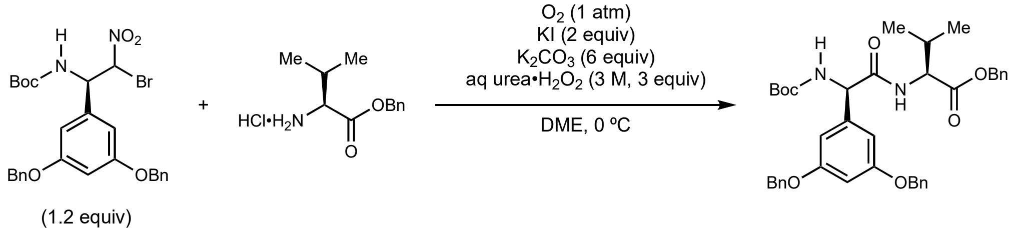

Benzyl ((*R*)-2-(3,5-bis(benzyloxy)phenyl)-2-((tert-butoxycarbonyl)amino)acetyl)-L-valinate (21). To a vigorously stirred mixture of amine (2.184 g, 8.959 mmol), α-bromo nitroalkane (6.000 g, 10.76 mmol), potassium iodide[20] (2.974 g, 17.92 mmol) and potassium carbonate (7.429 g, 53.76 mmol)[21] in 1,2-dimethoxyethane (90 mL) under oxygen was added a freshly prepared solution of urea-hydrogen peroxide in water (3.0 M, 9.0 mL, 27 mmol) over 2 h by syringe pump at 0 °C.[22] After the addition was complete, the mixture was stirred for an additional 22 h at the same temperature. Aq sodium thiosulfate was then added and the mixture was extracted with ethyl acetate. The organic layers were washed with 1 M aq HCl and brine, dried (sodium sulfate), and then concentrated *in vacuo*. The residue was purified by flash column chromatography (10-50% EtOAc in hexanes) or recrystallization[23] to give pure amide product (4.347 g, 74%). Mp = 107-109 °C (recrystallized from diethyl ether/hexanes); $[\alpha]_D^{20}$ -34 (*c* 0.49, CHCl₃); R_f = 0.46 (30% EtOAc/hexanes); IR (film) 3319, 2977, 2924, 1699, 1655, 1596, 1496, 1454, 1368, 1161, 1053, 698 cm^{-1}; ^{1}H NMR (400 MHz, $CDCl_3$) δ 7.40-7.31 (series of m, 15H), 6.63 (d, *J* = 2.1 Hz, 2H), 6.54 (dd, *J* = 2.3, 2.3 Hz, 1H), 6.21 (d, *J* = 9.0 Hz, 1H), 5.66 (br s, 1H), 5.18 (d, *J* = 12.2 Hz, 1H), 5.12 (d, *J* = 12.2 Hz, 1H), 5.05 (br s, 1H), 5.02 (d, *J* = 12.1 Hz, 2H), 5.00 (d, *J* = 11.9 Hz, 2H), 4.57 (d, *J* = 9.0, 4.7 Hz, 1H), 2.14-2.03 (m, 1H), 1.41 (s, 9H), 0.74 (d, *J* = 6.9 Hz, 3H), 0.65 (d, *J* = 6.9 Hz, 3H); ^{13}C NMR (100 MHz, $CDCl_3$) ppm 171.6, 169.7, 160.6, 155.1, 140.8, 136.8, 135.3, 128.83, 128.78, 128.7, 128.6, 128.5, 128.4, 128.2, 127.8, 127.7, 106.7, 106.4, 102.4, 80.3, 70.3, 67.3, 59.2, 57.2, 31.5, 28.4, 19.0, 17.3; HRMS (ESI): Exact mass calcd for $C_{39}H_{45}N_2O_7$ [M+H]$^+$ 653.3221, found 653.3225.

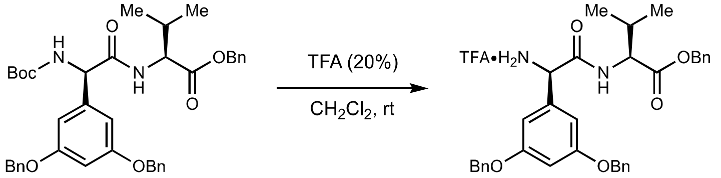

Benzyl ((*R*)-2-amino-2-(3,5-bis(benzyloxy)phenyl)acetyl)-L-valinate 2,2,2-trifluoroacetic acid (22). The dipeptide (51.4 mg, 13 µmol) was dissolved in trifluoroacetic acid (0.50 mL) and dichloromethane (2.0 mL), and the solution was stirred at rt under argon. Upon complete deprotection (approx. 1-2 h), the volatiles were removed by vacuum (15 Torr). Diethyl ether was added to the flask and the mixture was concentrated to remove residual trifluoroacetic acid. This was repeated twice

and the resulting solid was concentrated *in vacuo* to yield the crude product as an off-white solid (52.5 mg, quant.), which was used without purification.

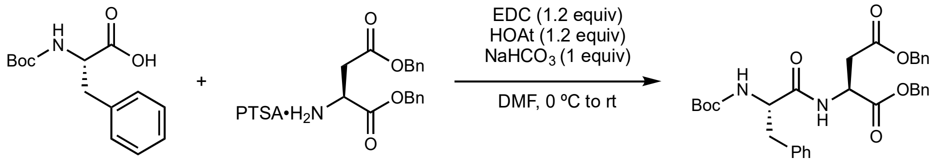

Dibenzyl (*tert*-butoxycarbonyl)-L-phenylalanyl-L-aspartate (23).[11] (*S*)-Aspartic acid dibenzylester p-toluenesulfonate (4.0086 g, 8.2558 mmol) was added to DMF (33 mL) under argon and cooled to 0 °C. (*S*)-*N*-Boc-phenylalanine (2.1936 g, 8.2681 mmol), EDC (1.8992 g, 9.9070 mmol), $NaHCO_3$ (693.6 mg, 8.2558 mmol), and HOAt (1.3484 g, 9.9070 mmol) were added and the mixture was stirred for 2 hours at 0 °C, then gradually warmed to room temperature overnight. After 2 days, the reaction was quenched with water and the aqueous phase was extracted with ethyl acetate. The combined organic phases were washed with 1 M aq HCl, satd $NaHCO_3$, and brine, dried, and concentrated. The crude solid was purified by flash chromatography (SiO_2, 10-50% EtOAc in hexanes) to afford the dipeptide as a white solid (4.1567 g, 90%). ^{1}H NMR (400 MHz, DMSO-d_6) δ 8.53 (d, *J* = 8.2 Hz, 1H), 7.38-7.28 (m, 10H), 7.27-7.13 (m, 5H), 6.90 (d, *J* = 8.7 Hz, 1H), 5.09 (s, 4H), 4.83-4.73 (m, 1H), 4.24-4.13 (m, 1H), 2.97-2.87 (m, 2H), 2.81 (dd, *J* = 16.3, 6.4 Hz, 1H), 2.67 (dd, *J* = 13.2, 11.6 Hz, 1H), 1.27 (s, 9H); ^{13}C NMR (100 MHz, DMSO-d_6) ppm 171.9, 170.4, 169.8, 155.2, 138.1, 135.8, 135.7, 129.1, 128.4, 128.3, 128.0 (3C), 127.8, 126.1, 78.0, 66.3, 65.9, 55.4, 48.6, 37.4, 35.8, 28.1. Analytical data (^{1}H NMR, ^{13}C NMR) were consistent with previous reports.[11]

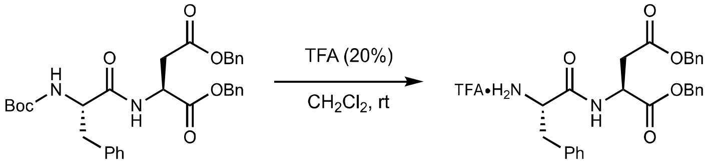

Dibenzyl L-phenylalanyl-L-aspartate, 2,2,2-trifluoroacetic acid (24). The dipeptide (26.6 mg, 47.4 µmol) was dissolved in trifluoroacetic acid (0.10 mL) and dichloromethane (0.40 mL) and was stirred at rt under argon. After complete deprotection by TLC (~1-2 h) the volatiles were removed by vacuum (15 Torr). Diethyl ether was added to the flask and the mixture was concentrated to remove residual trifluoroacetic acid. This was repeated twice and the resulting solid was concentrated *in vacuo* to yield the crude product as an off-white solid (27.3 mg, quant.) and was carried forward without purification.

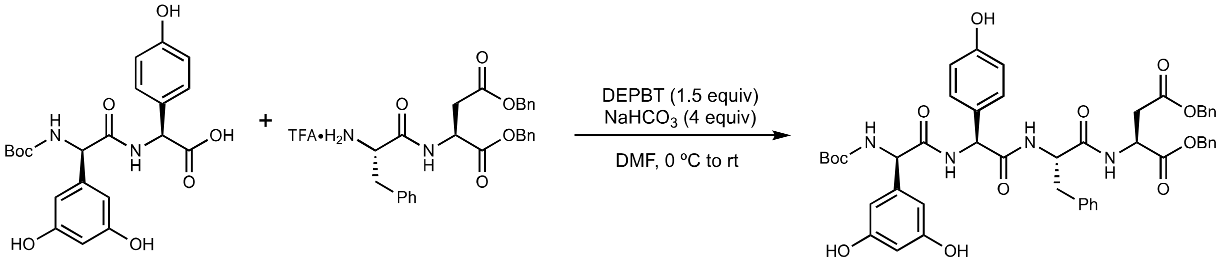

Dibenzyl ((*S*)-2-((*R*)-2-((*tert*-butoxycarbonyl)amino)-2-(3,5-dihydroxyphenyl)acetamido)-2-(4-hydroxyphenyl)acetyl)-L-phenylalanyl-L-aspartate (25).[11] The carboxylic acid (24.6 mg, 56.9 µmol) and amine (27.3 mg, 47.4 µmol) were dissolved in DMF (0.24 mL), and the solution was cooled to 0 °C. To the flask was added DEPBT (21.3 mg, 71.1 µmol) and $NaHCO_3$ (15.9 mg, 190 µmol). The reaction mixture was stirred at 0 °C for 2 hours, gradually warmed to room temperature, and stirred for 43 hours. The mixture was quenched with water and the aqueous layers were extracted several times with ethyl acetate. The organic layers were combined and washed with copious amounts of ice water followed by $NaHCO_3$ and brine. The organic layers were dried and concentrated. The resulting residue was purified by column chromatography (SiO_2, 20-60% (3:1 EtOAc:EtOH) in hexanes) to afford the product as a white solid (38.9 mg, 94%). ^{1}H NMR (400 MHz, DMSO-d_6) δ 9.32 (br s, 1H), 9.17 (br s, 2H), 8.51 (d, *J* = 7.9 Hz, 1H), 8.49 (d, *J* = 8.7 Hz, 1H), 8.31 (d, *J* = 8.2 Hz, 1H), 7.36-7.28 (m, 10H),

7.24-7.15 (m, 5H), 7.06 (d, *J* = 7.4 Hz, 1H), 6.91 (d, *J* = 8.5 Hz, 2H), 6.55 (d, *J* = 8.5 Hz, 2H), 6.26 (d, *J* = 2.0 Hz, 2H), 6.10 (dd, *J* = 2.0, 2.0 Hz, 1H), 5.24 (d, *J* = 7.3 Hz, 1H), 5.12 (d, *J* = 7.7 Hz, 1H), 5.10-5.03 (m, 4H), 4.76-4.65 (m, 1H), 4.61-4.50 (m, 1H), 3.06 (dd, *J* = 13.9, 3.6 Hz, 1H), 2.88 (dd, *J* = 16.6, 6.9 Hz, 1H), 2.81-2.72 (m, 2H), 1.38 (s, 9H); ^{13}C NMR (150 MHz, DMSO-d_6) ppm 170.7, 170.3, 169.9, 169.7, 169.6, 158.1, 156.7, 154.9, 140.3, 137.5, 135.8, 135.6, 129.2, 128.4 (2C), 128.3, 128.1 (2C), 128.0 (2C), 127.8, 126.2, 114.8, 105.4, 101.7, 78.4, 66.4, 66.0, 57.3, 55.9, 53.5, 48.6, 35.7, 28.2; HRMS (ESI): Exact mass calcd for $C_{48}H_{51}N_4O_{12}$ [M+H]$^+$ 875.3498, found 875.3499. Analytical data (^{1}H NMR, ^{13}C NMR, HRMS) were consistent with reported values.[11]

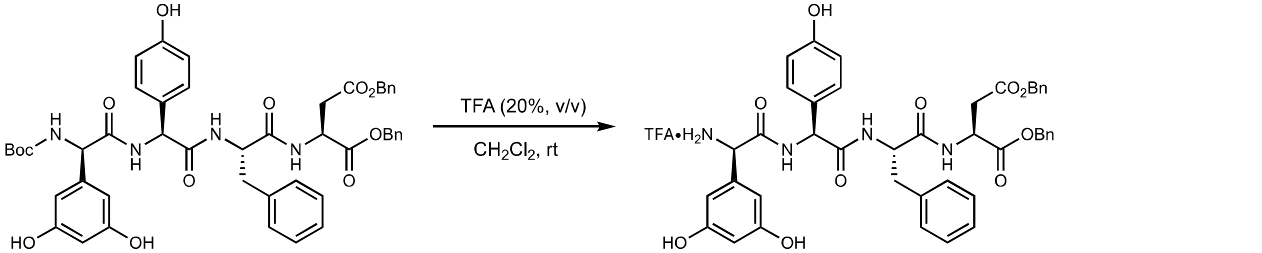

Dibenzyl ((*S*)-2-((*R*)-2-amino-2-(3,5-dihydroxyphenyl)acetamido)-2-(4-hydroxyphenyl)acetyl)-L-phenylalanyl-L-aspartate, 2,2,2-trifluoroacetic acid (26). The tetrapeptide (393.0 mg, 449.2 µmol) was dissolved in trifluoroacetic acid (1 mL) and dichloromethane (4 mL) and stirred at rt under an argon. After 3 h and 10 min, the volatiles were removed by vacuum (15 Torr). Diethyl ether was added to the flask and the mixture was concentrated to remove residual TFA. This was repeated twice and the resulting solid was concentrated *in vacuo* to yield the crude product as an off-white solid (quant.) which was carried forward without purification.

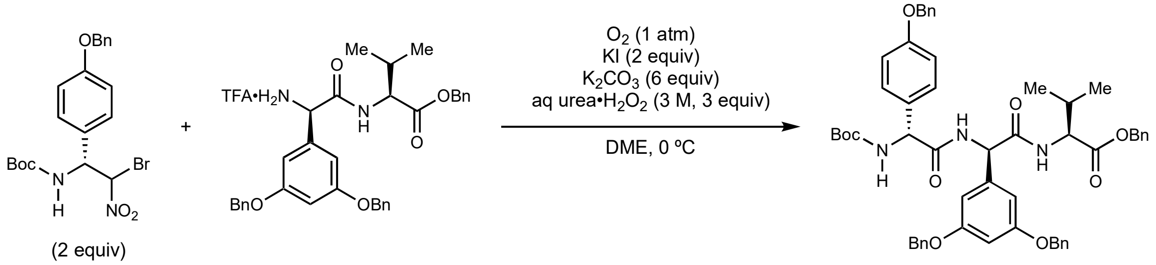

((*R*)-2-((*R*)-2-((*tert*-Butoxycarbonyl)amino)-2-(4-hydroxyphenyl)acetamido)-2-(3,5-dihydroxyphenyl)acetyl)-L-valine (27). The amine (242.1 mg, 363.2 µmol) and bromonitroalkane (327.8 mg, 726.4 µmol) was added to the flask, followed by DME (3.6 mL), and the mixture was cooled to 0 °C. Potassium iodide (120.6 mg, 726.4 µmol) was added, followed by potassium carbonate (301.2 mg, 2.179 mmol). A freshly prepared solution of urea-hydrogen peroxide in water was then added (0.36 mL, 1.1 mmol, 3 M), and the reaction was stirred under an oxygen atmosphere at 0 °C for 2 hours, and then at room temperature for 24 hours. The reaction was quenched with satd aq $Na_2S_2O_4$ and the aqueous layers were extracted several times with ethyl acetate. The organic layers were combined and washed with aq $Na_2S_2O_4$ followed by 1 M aq HCl and brine.[24] The organic layers were concentrated, and the resulting solid was precipitated from 2-MeTHF with hexanes to provide the product as a light-yellow solid (154.3 mg, 48%). $[\alpha]_D^{20}$ -64.8 (*c* 0.48, $CHCl_3$); R$_f$ = 0.19 (30% EtOAc/hexanes); IR (film) 3294, 3065, 2969, 1713, 1639, 1604, 1509, 1453 cm^{-1};^{1}H NMR (600 MHz, DMSO-d_6) δ 8.64 (d, *J* = 8.4 Hz, 1H), 8.62 (d, *J* = 8.2 Hz, 1H), 7.42 (d, *J* = 8.7 Hz, 1H), 7.44-7.32 (m, 22H), 6.92 (d, *J* = 8.46, 2H), 6.82 (d, *J* = 1.9 Hz, 2H), 6.56 (br s, 1H), 5.64 (d, *J* = 8.2 Hz, 1H), 5.30 (d, *J* = 9.0 Hz, 1H), 5.13 (br s, 2H), 5.05 (d, *J* = 11.8 Hz, 3H), 5.02 (d, *J* = 10.1 Hz, 3H), 4.18 (dd, *J* = 8.3, 6.4 Hz, 1H), 2.00 (dqq, *J* = 6.6, 6.6, 6.6 Hz, 1H), 1.36 (br s, 9H), 0.74 (d, *J* = 6.6 Hz, 3H), 0.69 (d, *J* = 6.4 Hz, 3H); ^{13}C NMR (150 MHz, DMSO-d_6) ppm 171.1, 169.9, 169.6, 159.3, 157.7, 154.9, 141.4, 137.0, 136.9, 135.8, 130.8, 128.6, 128.4(3C), 128.1, 128.0 (2C), 127.9, 127.8, 127.7 (2C), 127.6, 114.4, 105.9, 100.7, 78.5, 69.3, 69.1, 66.0, 57.3, 55.6, 30.2, 28.1, 18.7, 17.7; HRMS (APCI): Exact mass calcd for $C_{54}H_{58}N_3O_9$ [M+H]$^+$ 892.4173, found 892.4184.

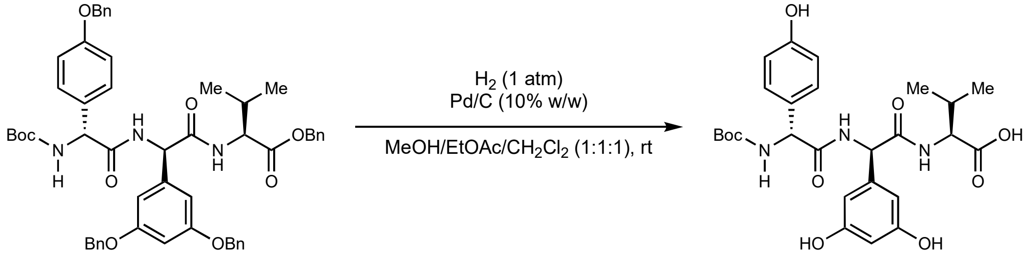

((*R*)-2-((*R*)-2-((*tert*-Butoxycarbonyl)amino)-2-(4-hydroxyphenyl)acetamido)-2-(3,5-dihydroxyphenyl)acetyl)-L-valine (28). The tripeptide (207.4 mg, 232.5 µmol) and palladium on carbon (10% w/w, 101.1 mg, 95.00 µmol) under argon were suspended in dichloromethane (1.6 mL), ethyl acetate (1.6 mL), and methanol (1.6 mL) respectively, and the flask was sealed with a septum. The flask was evacuated (50 Torr) and refilled with argon, and the process was repeated twice. The reaction flask was evacuated (50 Torr) and refilled with hydrogen five times. After 4 hours the reaction flask was evacuated and refilled with argon once, and the reaction mixture was filtered through a pad of Celite, and then washed with methanol. The volatiles were removed, CH_2Cl_2 was added to the resulting residue, and the solvent was removed at reduced pressure. This was repeated twice, and the resulting purple foam was dried under high vacuum for an hour (120.3 mg, 97%). ^{1}H NMR (600 MHz, DMSO-d_6) δ 9.46 (br s, 3H), 8.41 (d, *J* = 7.6 Hz, 1H), 7.26 (d, *J* = 8.2 Hz, 1H), 7.21 (d, *J* = 8.4 Hz, 1H), 7.16 (d *J* = 8.4 Hz, 2H), 6.65 (d, *J* = 8.4 Hz, 2H), 6.29 (d, *J* = 1.8 Hz, 2H), 6.10 (dd, *J* = 1.8, 1.8 Hz, 1H), 5.23 (d, *J* = 7.6 Hz, 1H), 5.18 (d, *J* = 8.4 Hz, 1H), 3.74 (dd, *J* = 8.3, 4.1 Hz, 1H), 1.92 (qqd, *J* = 7.1, 7.1, 4.1 Hz, 1H), 1.36 (s, 9H), 0.60 (d, *J* = 7.1 Hz, 3H), 0.59 (d, *J* = 7.1 Hz, 3H); ^{13}C NMR (150 MHz, DMSO-d_6) ppm 173.1, 169.7, 168.2, 158.3, 157.1, 154.7, 141.2, 129.0, 128.3, 115.0, 105.4, 101.7, 78.3, 59.1, 57.0, 56.8, 48.6, 31.2, 28.2, 19.6, 18.0; HRMS (ESI): Exact mass calcd for $C_{26}H_{33}N_3NaO_9$ $[M+Na]^+$ 554.2114, found 554.2126. Remaining analytical data (HRMS) were consistent with a previous report.[11,25]

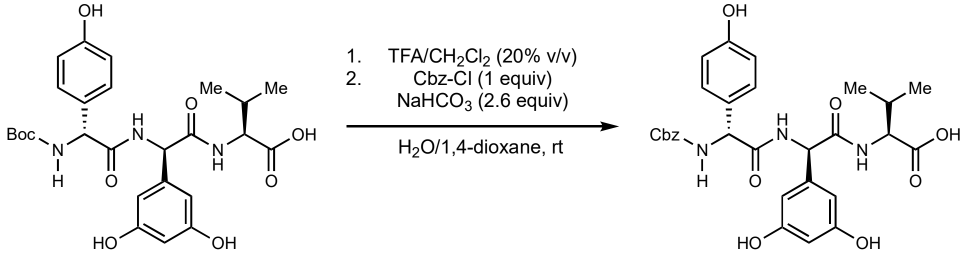

((*R*)-2-((*R*)-2-(((Benzyloxy)carbonyl)amino)-2-(4-hydroxyphenyl)acetamido)-2-(3,5-dihydroxyphenyl)acetyl)-L-valine (30).[26] The tripeptide (73.0 mg, 137 µmol) was dissolved in dichloromethane (1.6 mL) and trifluoroacetic acid (0.4 mL) and stirred under argon. After one hour the volatiles were removed. Diethyl ether was added to the flask and the mixture was concentrated to remove excess trifluoroacetic acid. This was repeated twice and the resulting off-white foam was dried under high vacuum for an hour. The amine salt was then dissolved in a 1:1 mixture of H_2O/dioxane (1.34 mL), and $NaHCO_3$ (29.7 mg, 354 µmol) was added to the flask. CbzCl (33 µL, 137 µmol) in 1,4-dioxane (0.67 mL) was added dropwise via syringe pump over 45 minutes to the reaction mixture. The reaction mixture was stirred overnight, and then water was added. The aqueous phase was washed with ethyl acetate, and then acidified to pH 3 with 1 M aq HCl. The suspension was then extracted with ethyl acetate, and the combined organic layers were washed with 1 M aq HCl, water, and brine. The organic phase was dried and concentrated to afford the desired tripeptide as a white solid (76.3 mg, 98%). ^{1}H NMR (600 MHz, DMSO-d_6) δ 9.37 (br s, 1H), 9.18 (br s, 2H), 8.52 (d, *J* = 8.1 Hz, 1H), 8.15 (d, *J* = 8.8 Hz, 1H), 7.82 (d, *J* = 8.8 Hz, 1H), 7.37-7.33 (m, 4H), 7.32-7.30 (m, 1H), 7.21 (d, *J* = 8.6 Hz, 2H), 6.67 (d, *J* = 8.6 Hz, 2H), 6.33 (d, *J* = 1.9 Hz, 2H), 6.12 (dd, *J* = 1.9, 1.9 Hz, 1H), 5.45 (d, *J* = 8.1 Hz, 1H), 5.31 (d, *J* = 8.8 Hz, 1H), 5.02 (s, 2H), 4.10 (dd, *J* = 8.8, 5.7 Hz, 1H), 1.95 (qqd, *J* = 6.9, 6.9, 5.7 Hz, 1H), 0.71 (d, *J* = 6.9 Hz, 3H), 0.68 (d, *J* = 6.9 Hz, 3H); ^{13}C NMR (150 MHz, DMSO-d_6) ppm 172.8, 169.8, 169.7, 158.1, 156.8, 155.5, 140.8, 137.0, 128.9, 128.5, 128.4, 128.1, 127.8, 127.7, 126.6, 126.4, 115.0, 105.5, 101.7, 65.6, 62.9, 57.4, 56.9, 55.9, 30.3, 19.0, 17.6; HRMS (ESI): Exact mass calcd for $C_{29}H_{32}N_3O_9$ $[M+H]^+$ 566.2139, found 566.2115. Analytical data (^{1}H NMR, ^{13}C NMR, HRMS) were consistent with previous reports.[11]

[1] Dettner, F.; Hänchen, A.; Schols, D.; Toti, L.; Nußer, A.; Süssmuth, Roderich D.; *Angew. Chem. Int. Ed.* **2009** *48*, 1856.
[2] Fuse, S.; Mifune, Y.; Nakamura, H.; Tanaka, H.; *Nat. Commun.* **2016** *7*, 13491.
[3] Marschall, E.; Cass, R. W.; Prasad, K. M.; Swarbrick, J. D.; McKay, A. I.; Payne, J. A. E.; Cryle, M. J.; Tailhades, J.; *Chem. Sci.* **2024** *15*, 195.
[4] Pangborn, A. B.; Giardello, M. A.; Grubbs, R. H.; Rosen, R. K.; Timmers, F. J. *Organometallics* **1996**, *15*, 1518.
[5] On large scale (> 5 g), some residual aldehyde impurity may be present in the isolated solid. While the presence of the aldehyde will not negatively impact the subsequent aza-Henry reaction, it can be removed by suspending the white solid in diethyl ether, stirring overnight, and filtering the solid.
[6] The diastereomeric ratio of the bromonitroalkane can vary between 1:1 to 1:3 dr. If the reaction proceeds longer than 24 h, an enrichment in the dr may be observed.
[7] Yedage, S. L.; Bhanage, B. M.; *J. Org. Chem.* **2017** *82*, 5769.
[8] Ellman, J. A. R., S.D.;Lacour, J.; *J. Am. Chem. Soc.* **1997** *119*, 3419.
[9] COO*H* not observed due to broadening.
[10] The ability to effect the hydrogenolysis was highly dependent on the quality of the Pd/C. For difficult hydrogenolyses, a 1:1 mixture of Pd/C (10% w/w) and $Pd(OH)_2/C$ (20% w/w) at 10 mol% Pd per benzyl was used with more reproducible results. Moreover, Pd/C (10% w/w) purchased from Strem led to consistently faster reactions with more reproducible results.
[11] Dettner, F.; Hänchen, A.; Schols, D.; Toti, L.; Nußer, A.; Süssmuth, R. D.; *Angew. Chem. Int. Ed.* **2009** *48*, 1856.
[12] Some differences were noted when comparing NMR spectra of **9** with ref. 11. However, ^{1}H NMR data of subsequent coupled products were consistently identical, so these small differences were attributed to the presence of residual methanol in the characterized NMR spectra of ref. 11.
[13] On larger scales (>500 mg), peptide can be purified by passage through a silica plug with ethyl acetate followed by trituration with diethyl ether.
[14] Internal temperature should be monitored as epimerization may occur if DEPBT is added without rigorously cooling to 0 °C.
[15] This compound streaks on the TLC plate.
[16] Seven of nine phenolic O-*H* were observed.
[17] A diethyl phosphate impurity was noted in this material, similar to a previous report: Fuse, S.; Mifune, Y.; Nakamura, H.; Tanaka, H.; *Nat. Commun.* **2016** *7*, 13491.). The yield for this step was calculated without estimating the impurity amount. Therefore the yield of the subsequent reaction is slightly depressed relative to actual.
[18] Ten of eleven phenolic O-*H* were observed
[19] The diastereomeric ratio of the bromonitroalkane can vary between 1:1 to 3:1 dr. If the reaction proceeds longer than 24 h, an enrichment in the dr may be observed without change in ee.
[20] For best results, the potassium iodide should be ground by mortar & pestle prior to use.
[21] Potassium carbonate should be added just before addition of the urea-hydrogen peroxide solution to prevent decomposition of the bromonitroalkane.
[22] A small piece of Tygon tubing was used in place of a metal needle for the addition of the urea hydrogen peroxide to prevent iron-catalyzed decomposition of the hydrogen peroxide.
[23] On gram-scale the peptide can be purified by recrystallization from diethyl ether after passage through a silica plug with ethyl acetate.
[24] At this point on large scale, much precipitate may be observed. If so, the solids are collected prior to concentration.
[25] Some differences were noted when comparing NMR spectra of **28** with ref. 11. However, ^{1}H NMR data of subsequent coupled products were consistently identical, so these small differences were attributed to sensitivity of **28** to sample preparation and conditions (e.g. concentration, temperature).
[26] Procedure adapted from ref. 11.

NMR Spectroscopic Data

Contents

Figure S1. ^{1}H NMR (400 MHz, $CDCl_3$) of 1 3
Figure S2. ^{13}C NMR (100 MHz, $CDCl_3$) of 1 4
Figure S3. ^{1}H NMR (600 MHz, $CDCl_3$) of (*S*)-3 5
Figure S4. ^{13}C NMR (150 MHz, $CDCl_3$) of (*S*)-3 6
Figure S5. ^{1}H NMR (600 MHz, $CDCl_3$) of 4 7
Figure S6. ^{13}C NMR (150 MHz, $CDCl_3$) of 4. 8
Figure S7. ^{1}H NMR (600 MHz, DMSO-d_6) of 6 9
Figure S8. ^{13}C NMR (150 MHz, DMSO-d_6) of 6. 10
Figure S9. ^{1}H NMR (600 MHz, $CDCl_3$) of 8 11
Figure S10. ^{13}C NMR (150 MHz, $CDCl_3$) of 8 12
Figure S11. ^{1}H NMR (400 MHz, DMSO-d_6) of 9 13
Figure S12. ^{13}C NMR (150 MHz, DMSO-d_6) of 9 14
Figure S13. ^{1}H NMR (600 MHz, DMSO-d_6) of 10 15
Figure S14. ^{13}C NMR (150 MHz, DMSO-d_6) of 10 16
Figure S15. ^{1}H NMR (600 MHz, DMSO-d_6) of 12 17
Figure S16. ^{13}C NMR (150 MHz, DMSO-d_6) of 12 18
Figure S17. ^{1}H NMR (600 MHz, DMSO-d_6) of 13 19
Figure S18. ^{13}C NMR (150 MHz, DMSO-d_6) of 13 20
Figure S19. ^{1}H NMR (600 MHz, DMSO-d_6) of 14 21
Figure S20. ^{13}C NMR (150 MHz, DMSO-d_6) of 14 22
Figure S21.^{1}H NMR (600 MHz, DMSO-d_6) of 16 23
Figure S22. ^{13}C NMR (150 MHz, DMSO-d_6) of 16 24
Figure S23. HSQC (600 MHz, DMSO-d_6) of 16 25
Figure S24. Crude ^{1}H NMR (600 MHz, DMSO-d_6) of 17 26
Figure S25. ^{1}H NMR (900 MHz, DMSO-d_6) of 17 27
Figure S26. ^{13}C NMR (225 MHz, DMSO-d_6) of 17 28
Figure S27. HSQC (900 MHz, DMSO-d_6) of 17 29
Figure S28. ^{1}H NMR (400 MHz, $CDCl_3$) of 18 30
Figure S29. ^{13}C NMR (100 MHz, $CDCl_3$) of 18 31
Figure S30. ^{1}H NMR (400 MHz, DMSO-d_6) of 20 32
Figure S31. ^{13}C NMR (100 MHz, DMSO-d_6) of 20 33
Figure S32. ^{1}H NMR (600 MHz, $CDCl_3$) of 21 34

Figure S33. ^{13}C NMR (150 MHz, $CDCl_3$) of 21 35
Figure S34. ^{1}H NMR (400 MHz, DMSO-d_6) of 23 36
Figure S35. ^{13}C NMR (100 MHz, DMSO-d_6) of 23 37
Figure S36. ^{1}H NMR (600 MHz, DMSO-d_6) of 25 38
Figure S37. ^{13}C NMR (150 MHz, DMSO-d_6) of 25 39
Figure S38. ^{1}H NMR (600 MHz, DMSO-d_6) of 27 40
Figure S39. ^{13}C NMR (150 MHz, DMSO-d_6) of 27 41
Figure S40. ^{1}H NMR (600 MHz, DMSO-d_6) of 28 42
Figure S41. ^{13}C NMR (150 MHz, DMSO-d_6) of 28 43
Figure S42. ^{1}H NMR (600 MHz, DMSO-d_6) of 30 44
Figure S43. ^{13}C NMR (150 MHz, DMSO-d_6) of 30 45
Figure S44. HPLC chromatogram of *rac*-3 46
Figure S45. HPLC *c*hromatogram of (*S*)-3 before recrystallization 46
Figure S46. HPLC *c*hromatogram of (*S*)-3 after recrystallization 47
Figure S47. HPLC chromatogram of *rac*-20 47
Figure S48. HPLC chromatogram of (*R*)-20 before recrystallization 48
Figure S49. HPLC chromatogram of (*R*)-20 after recrystallization 48

Figure S1. ^{1}H NMR (400 MHz, $CDCl_3$) of **1**

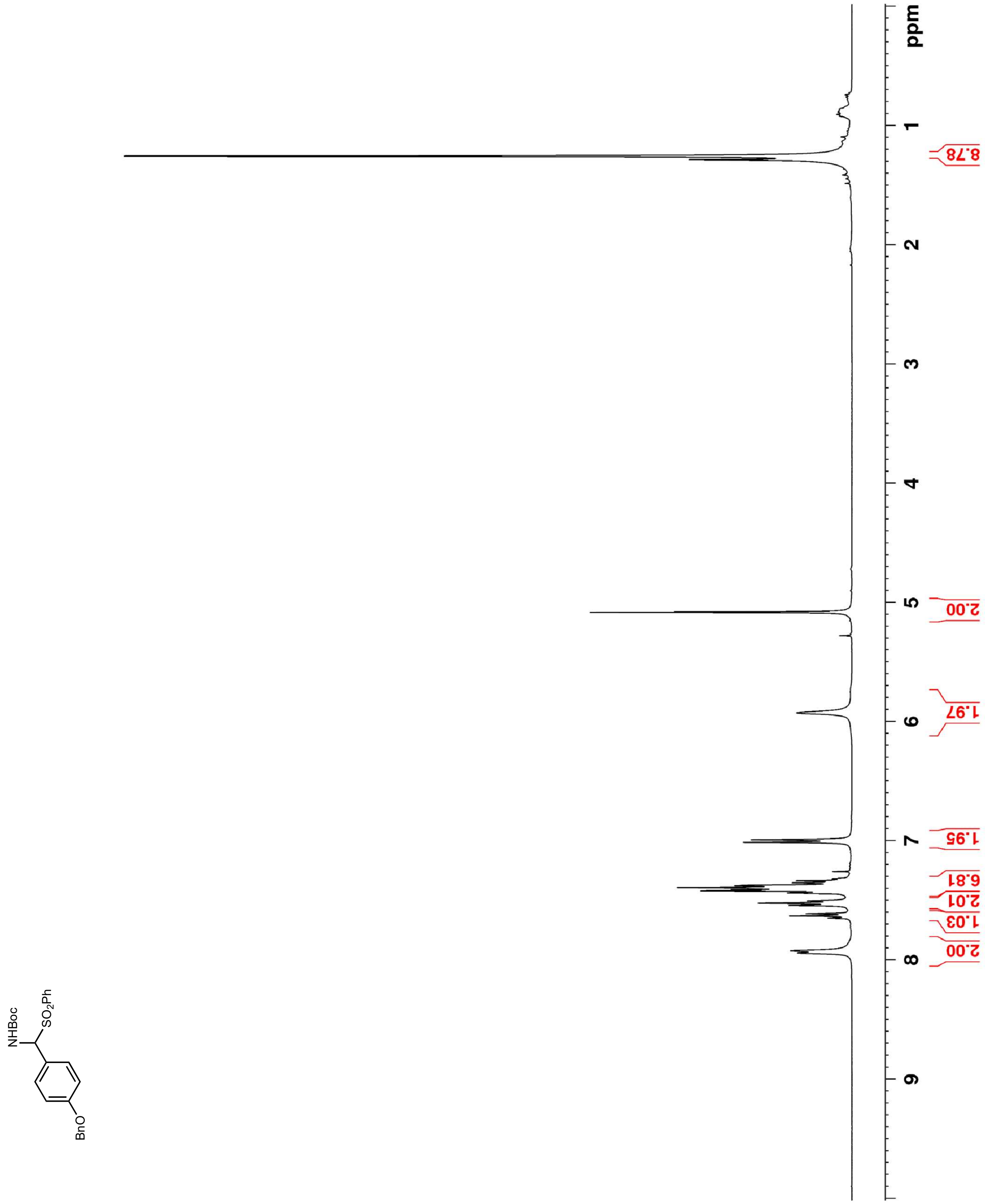

Figure S2. ^{13}C NMR (100 MHz, $CDCl_3$) of **1**

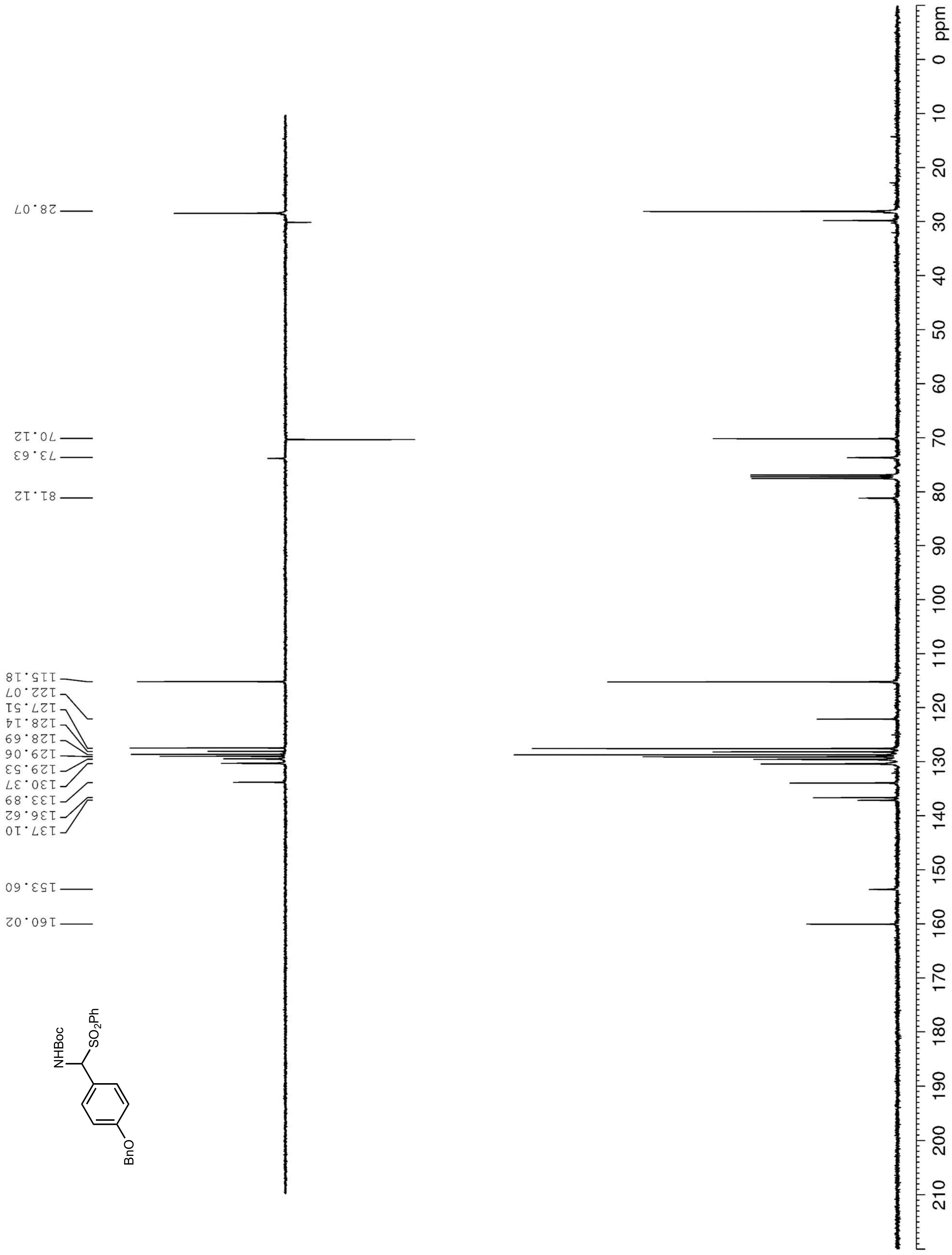

Figure S3. ^{1}H NMR (600 MHz, $CDCl_3$) of (*S*)-**3**

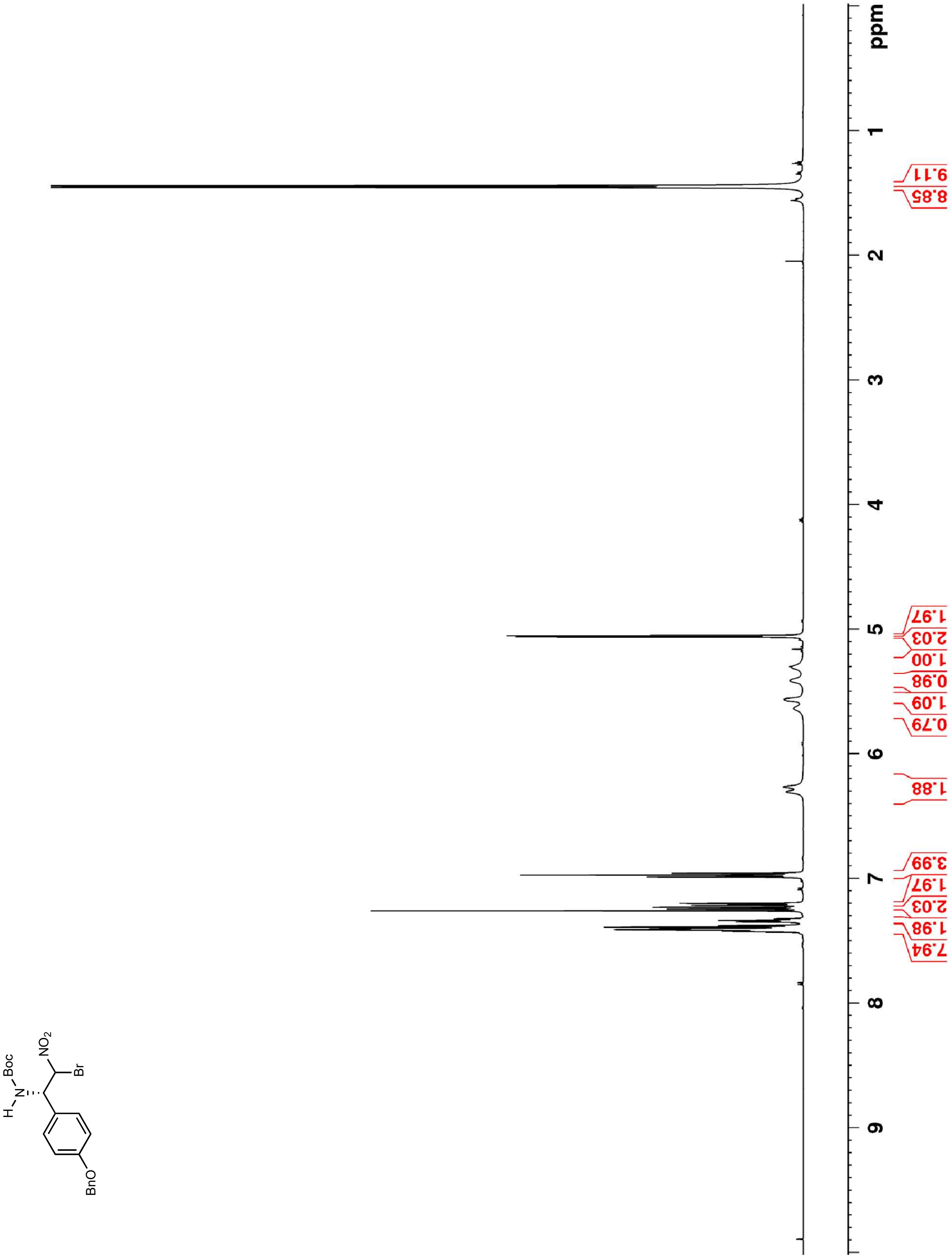

Figure S4. ^{13}C NMR (150 MHz, $CDCl_3$) of (*S*)-**3**

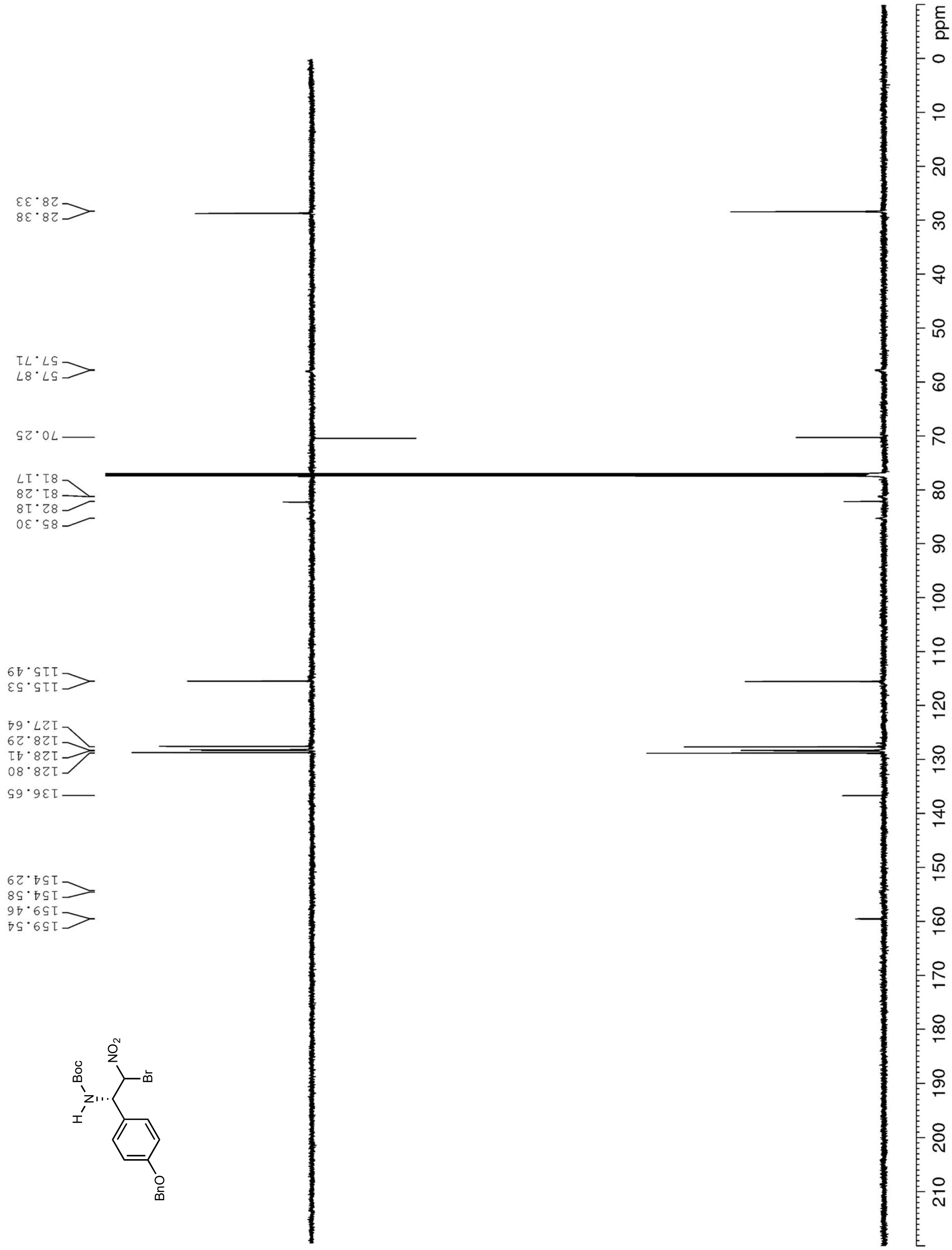

Figure S5. ^{1}H NMR (600 MHz, $CDCl_3$) of **4**

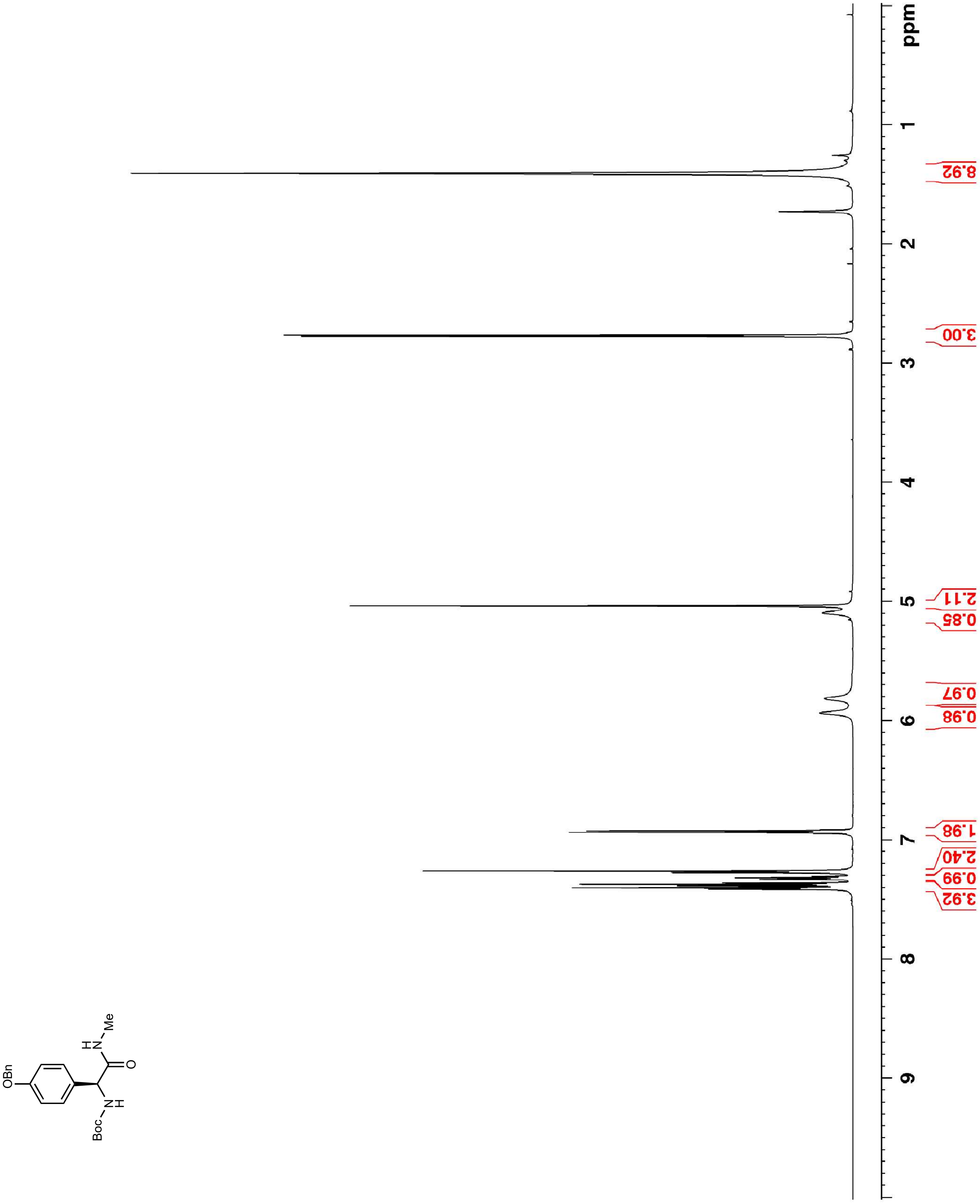

Figure S6. ^{13}C NMR (150 MHz, $CDCl_3$) of **4.**

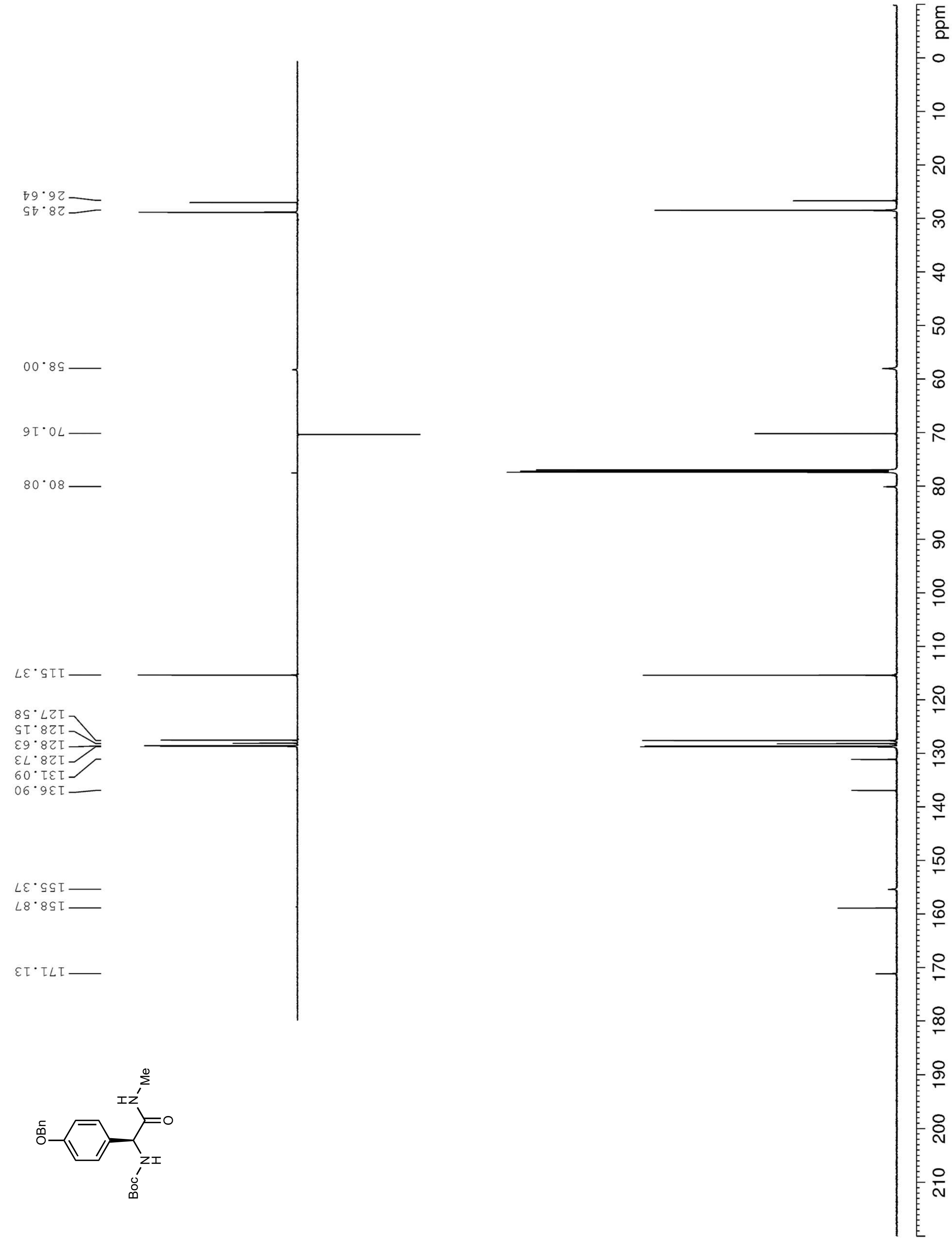

Figure S7. ^{1}H NMR (600 MHz, DMSO-d_6) of **6**

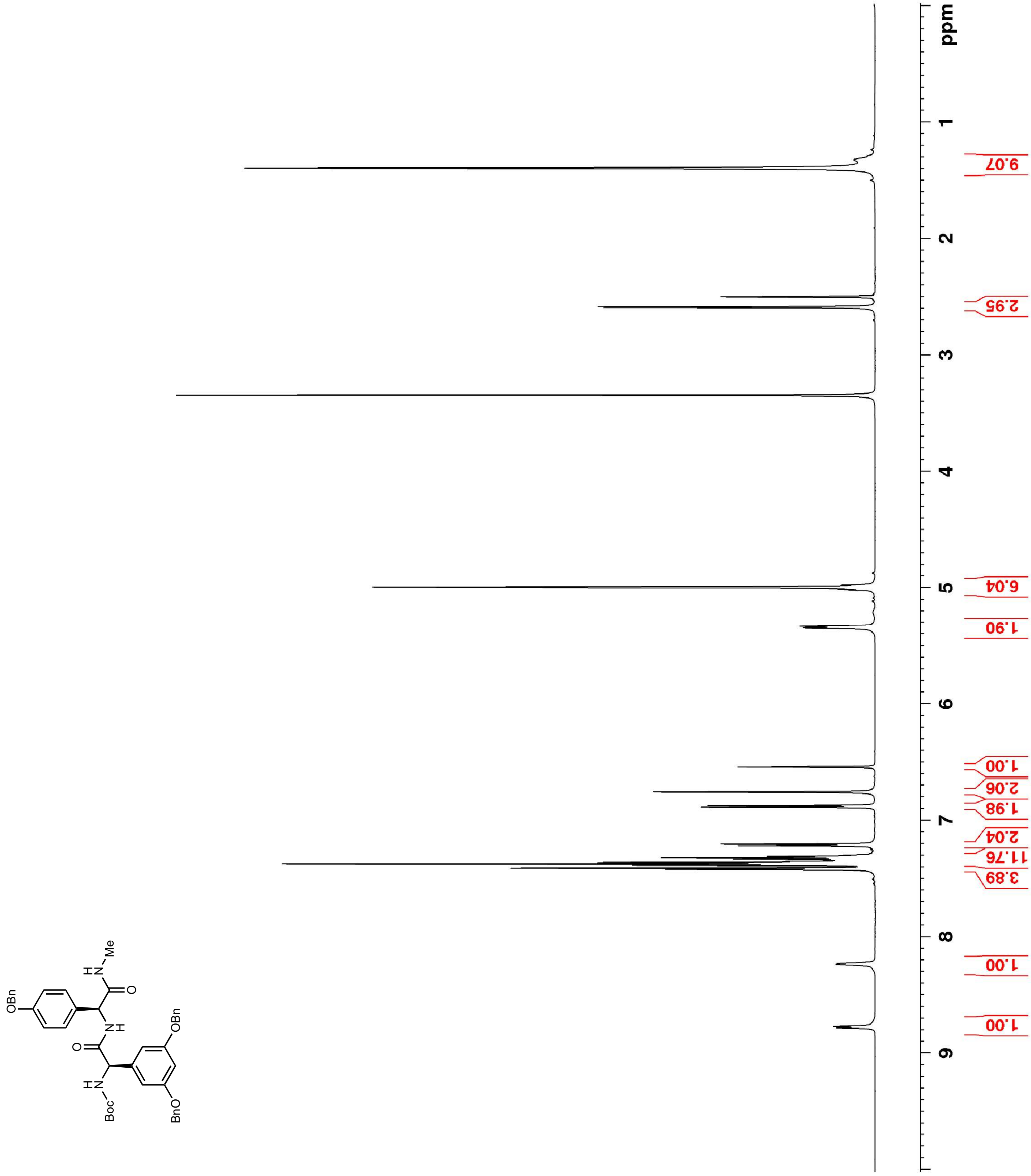

Figure S8. ^{13}C NMR (150 MHz, DMSO-d_6) of **6.**

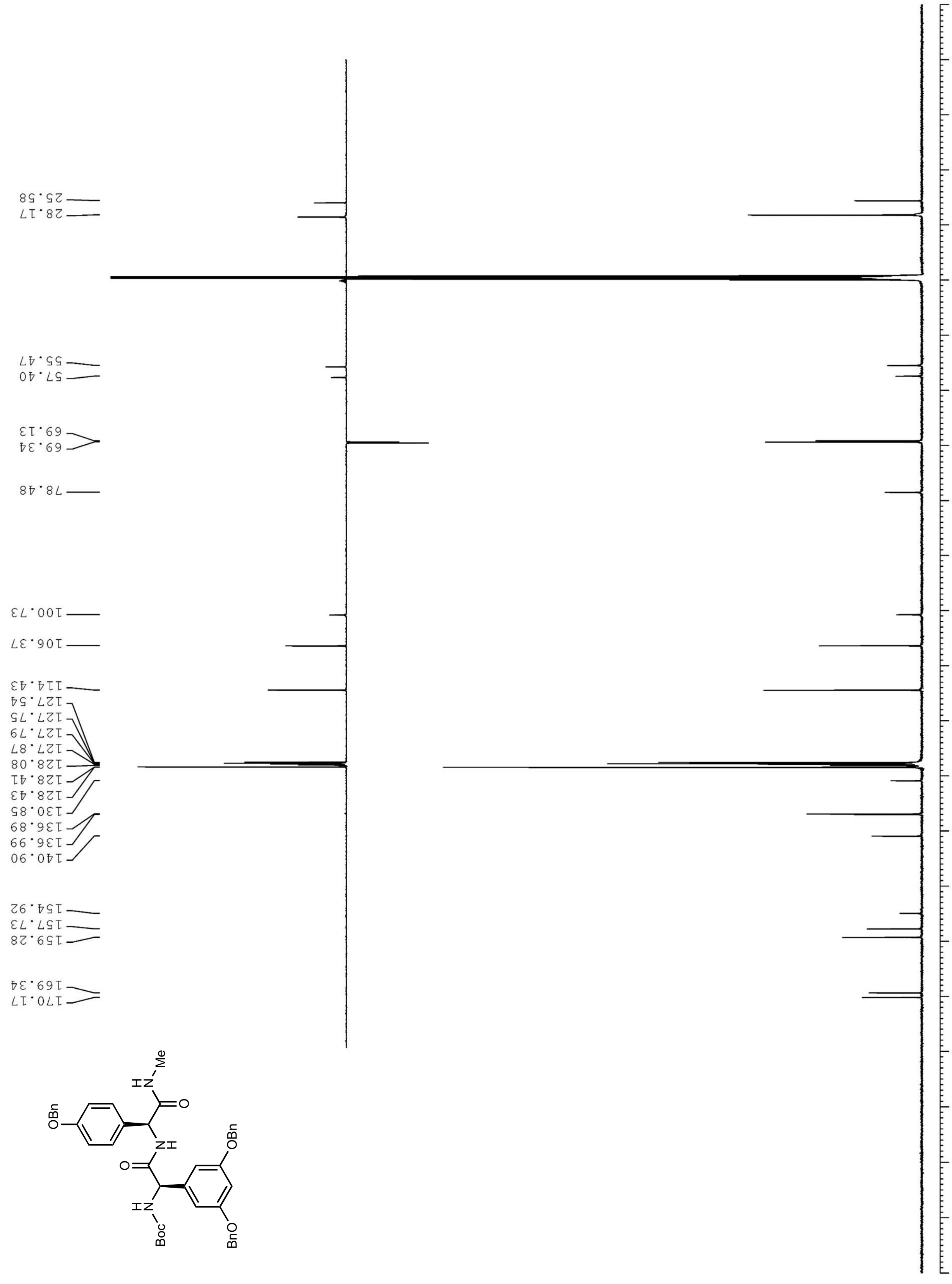

Figure S9. ^{1}H NMR (600 MHz, $CDCl_3$) of **8**

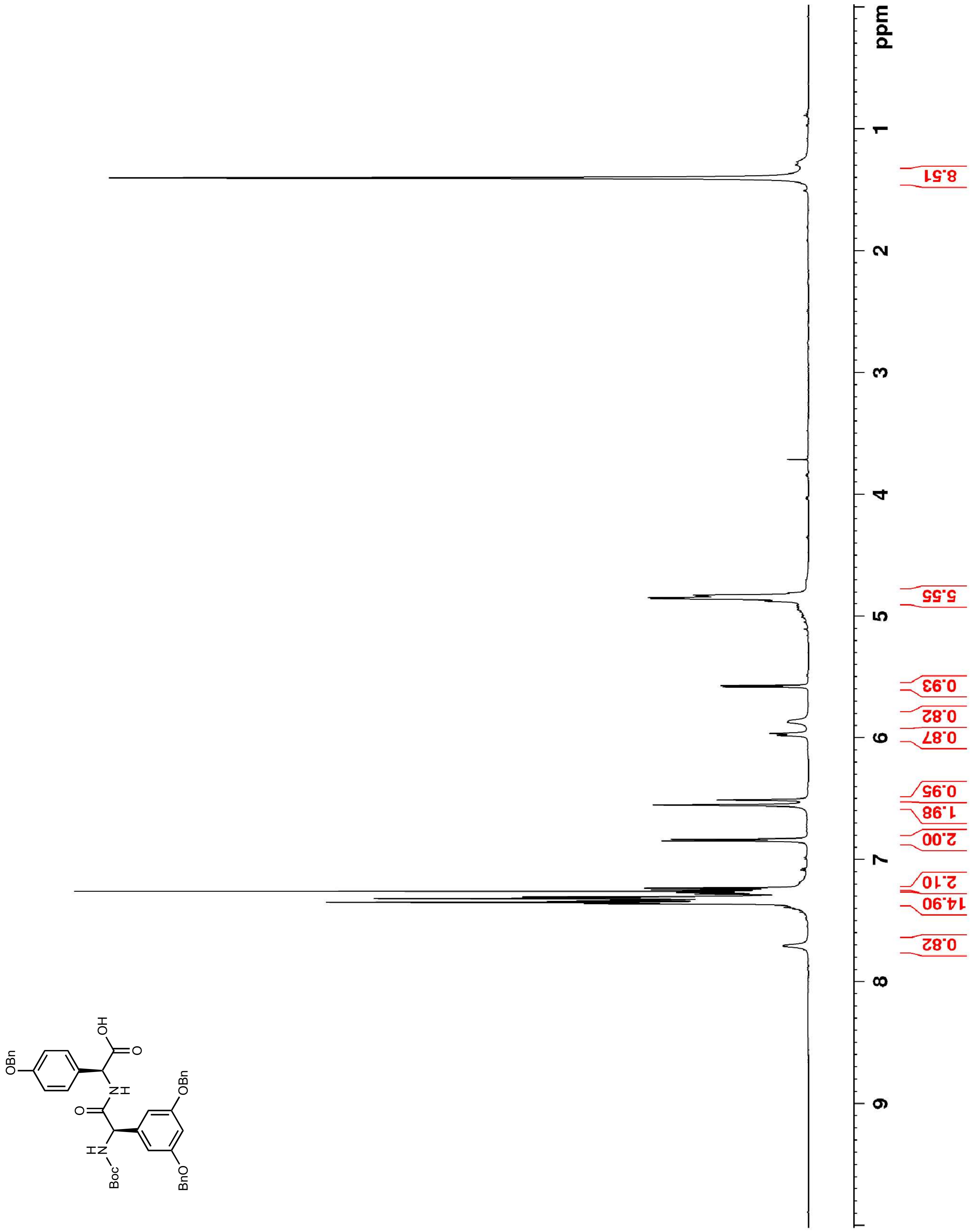

Figure S10. ^{13}C NMR (150 MHz, $CDCl_3$) of **8**

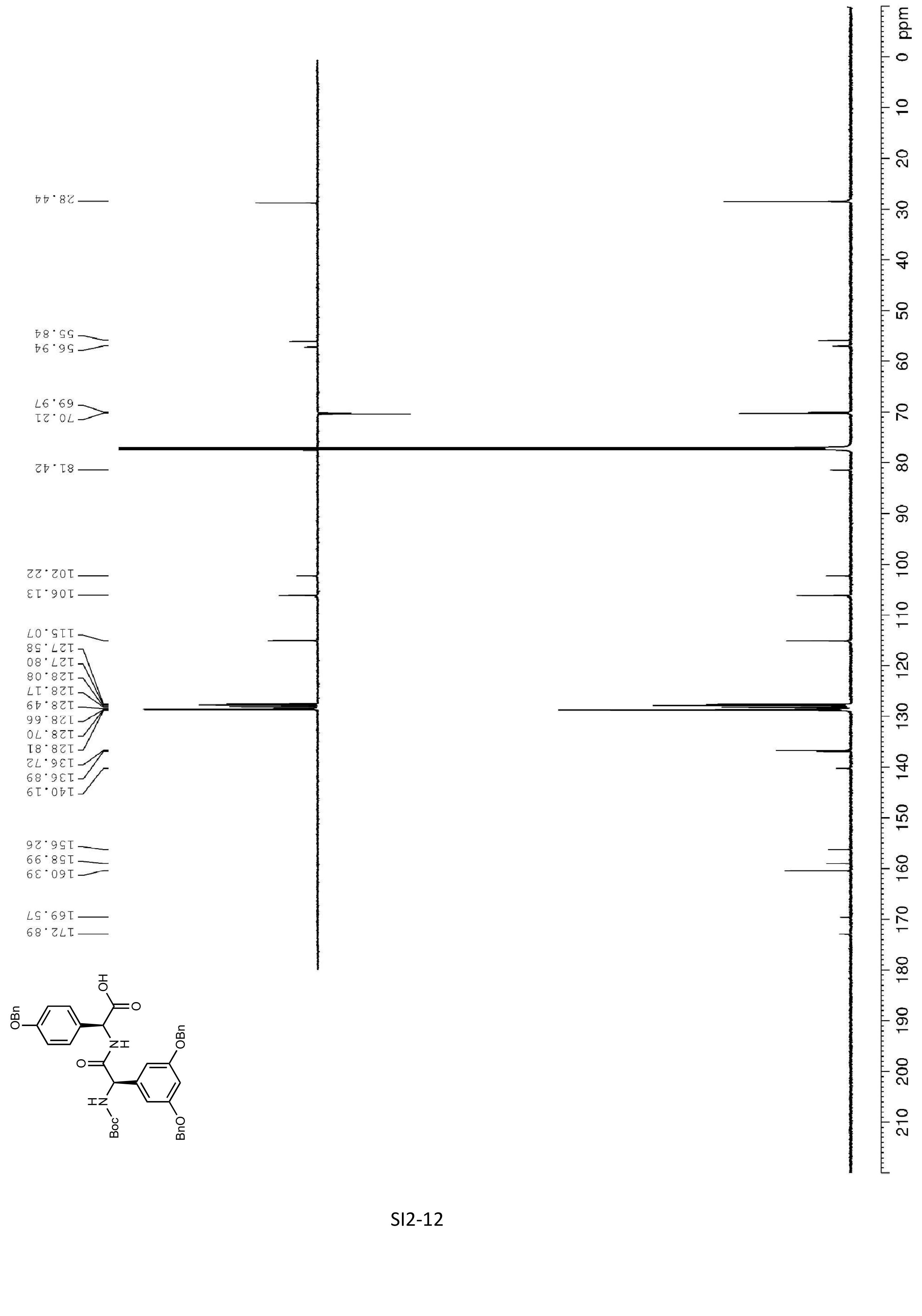

Figure S11. ^{1}H NMR (400 MHz, DMSO-d_6) of 9

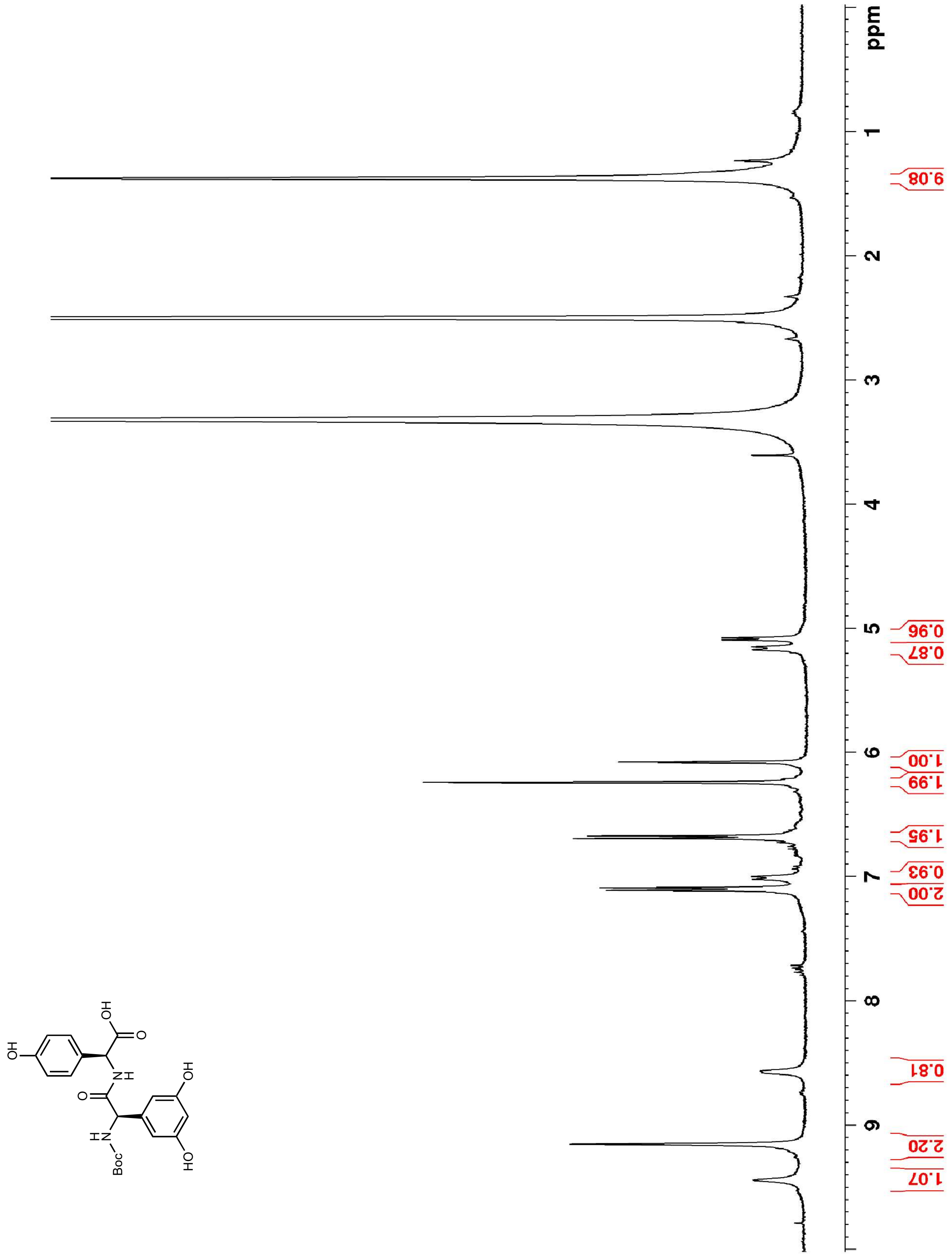

Figure S12. ^{13}C NMR (150 MHz, DMSO-d_6) of 9

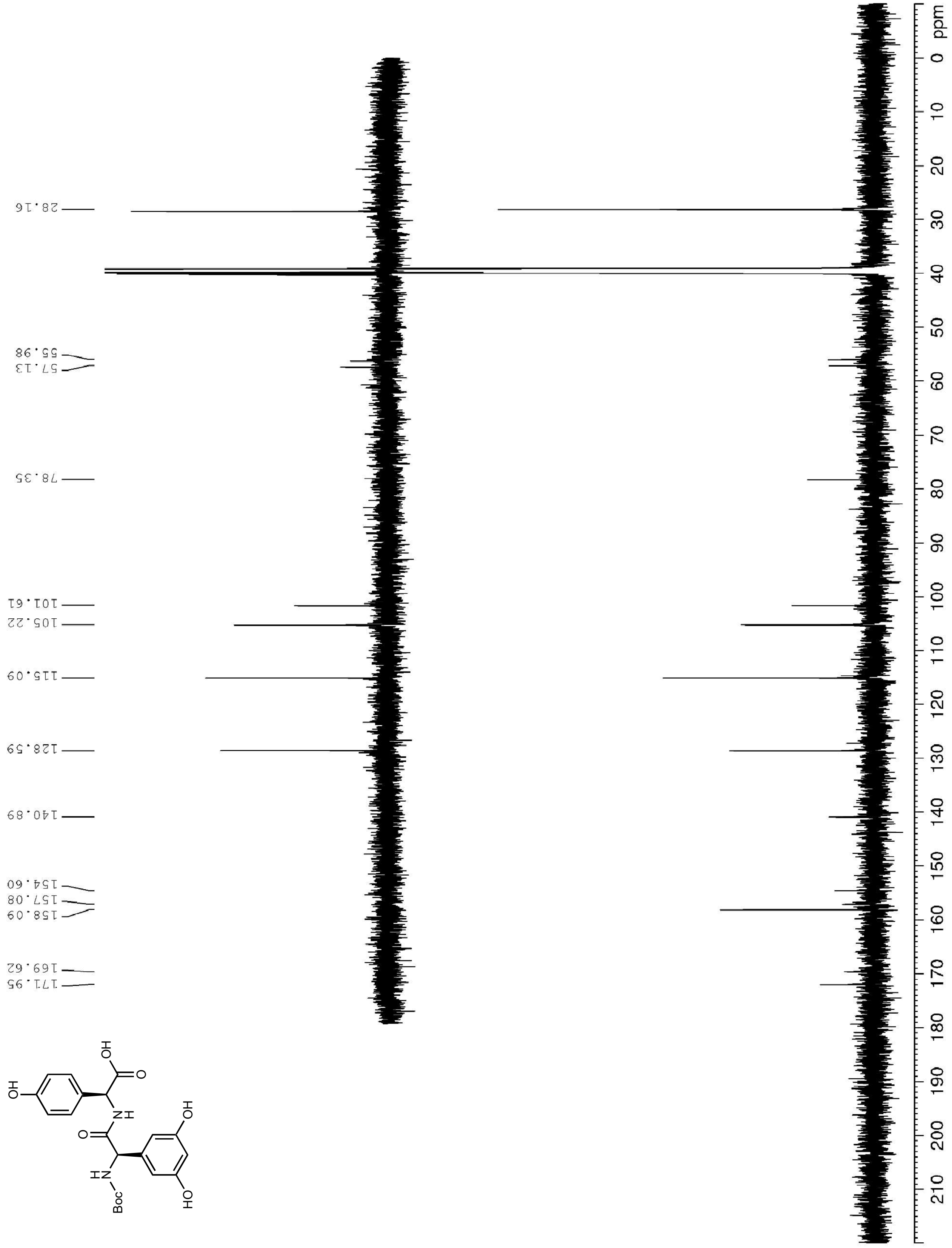

Figure S13. ^{1}H NMR (600 MHz, DMSO-d_6) of 1**0**

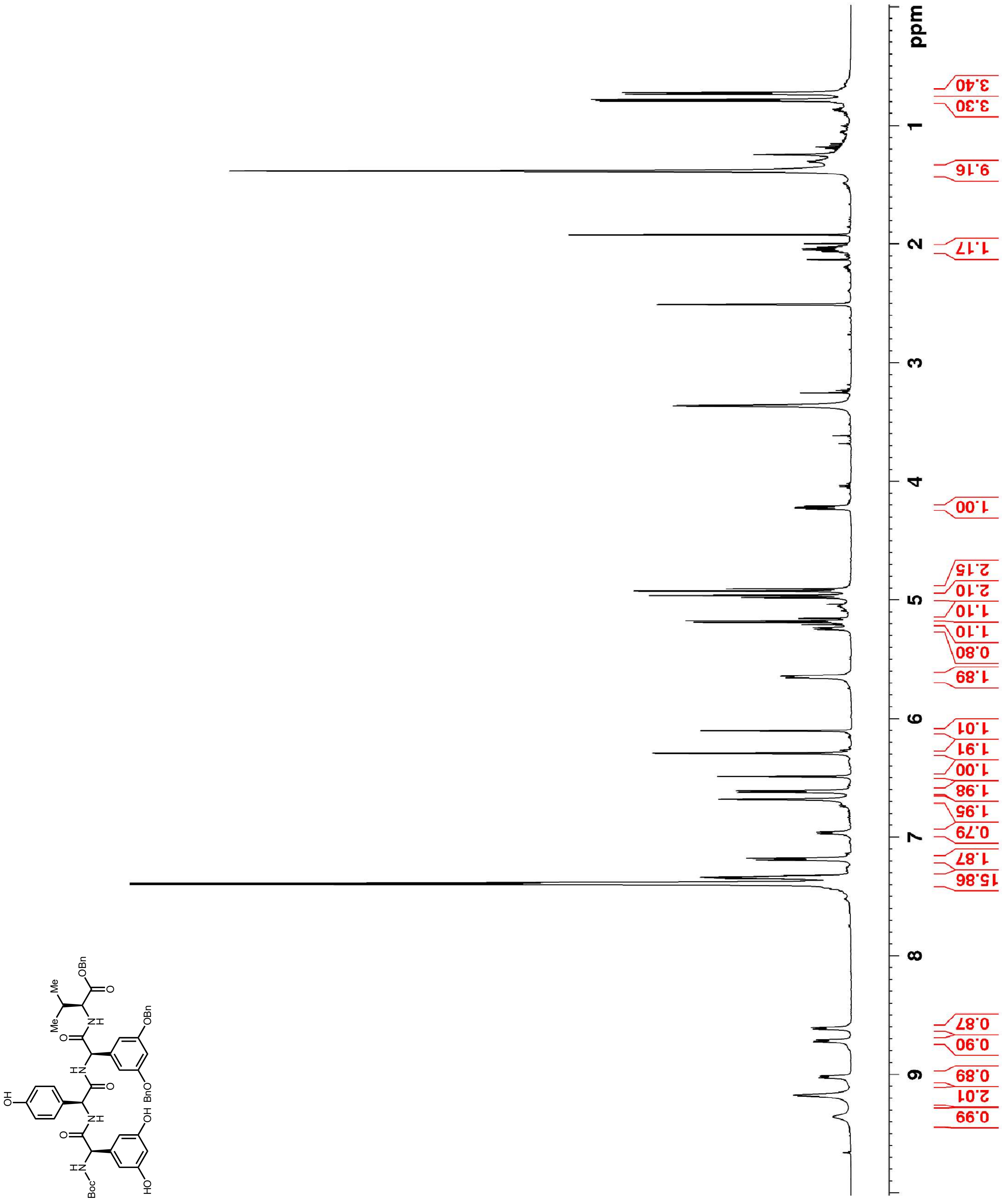

Figure S14. ^{13}C NMR (150 MHz, DMSO-d_6) of **10**

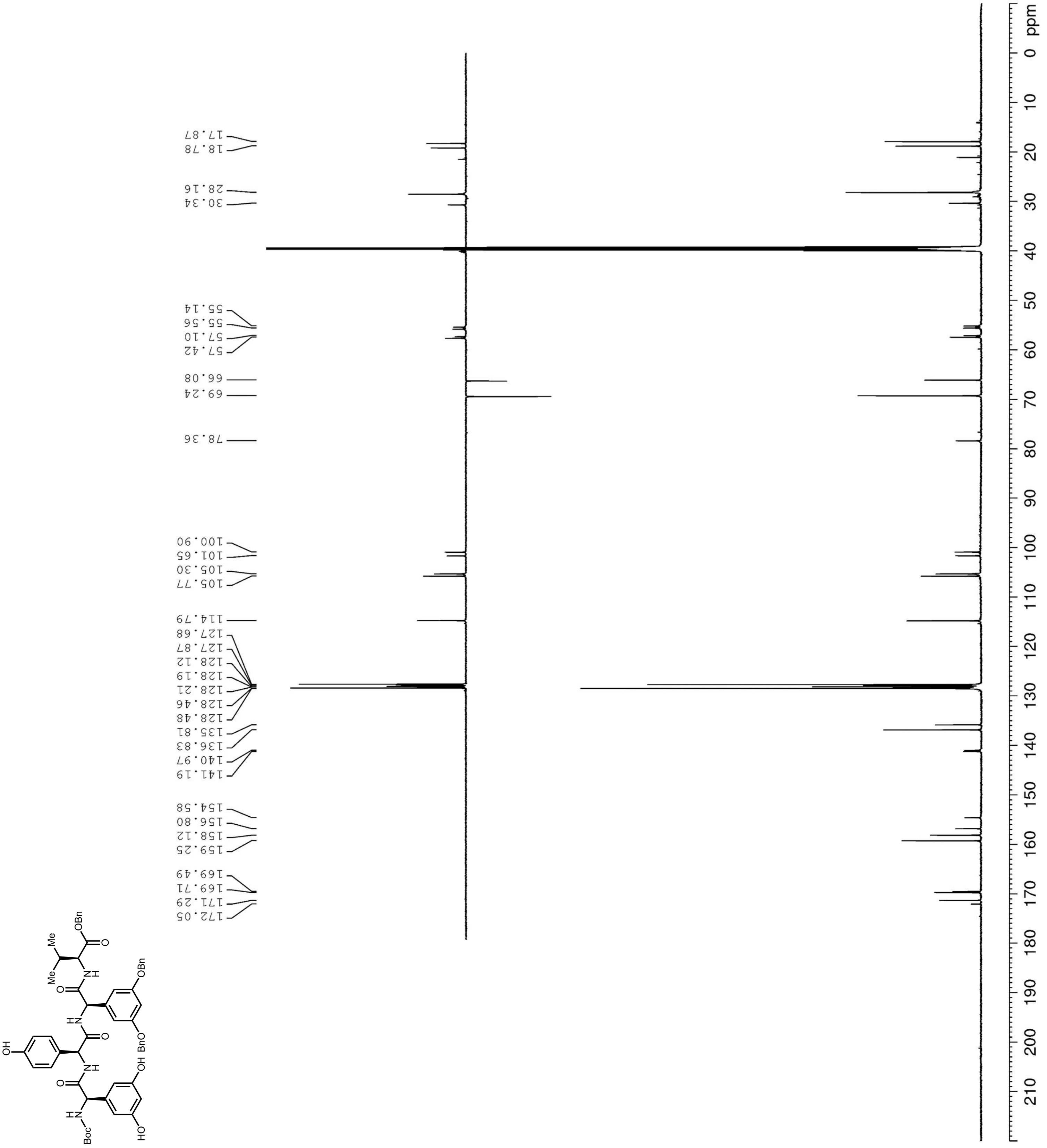

Figure S15. ^{1}H NMR (600 MHz, DMSO-d_6) of **12**

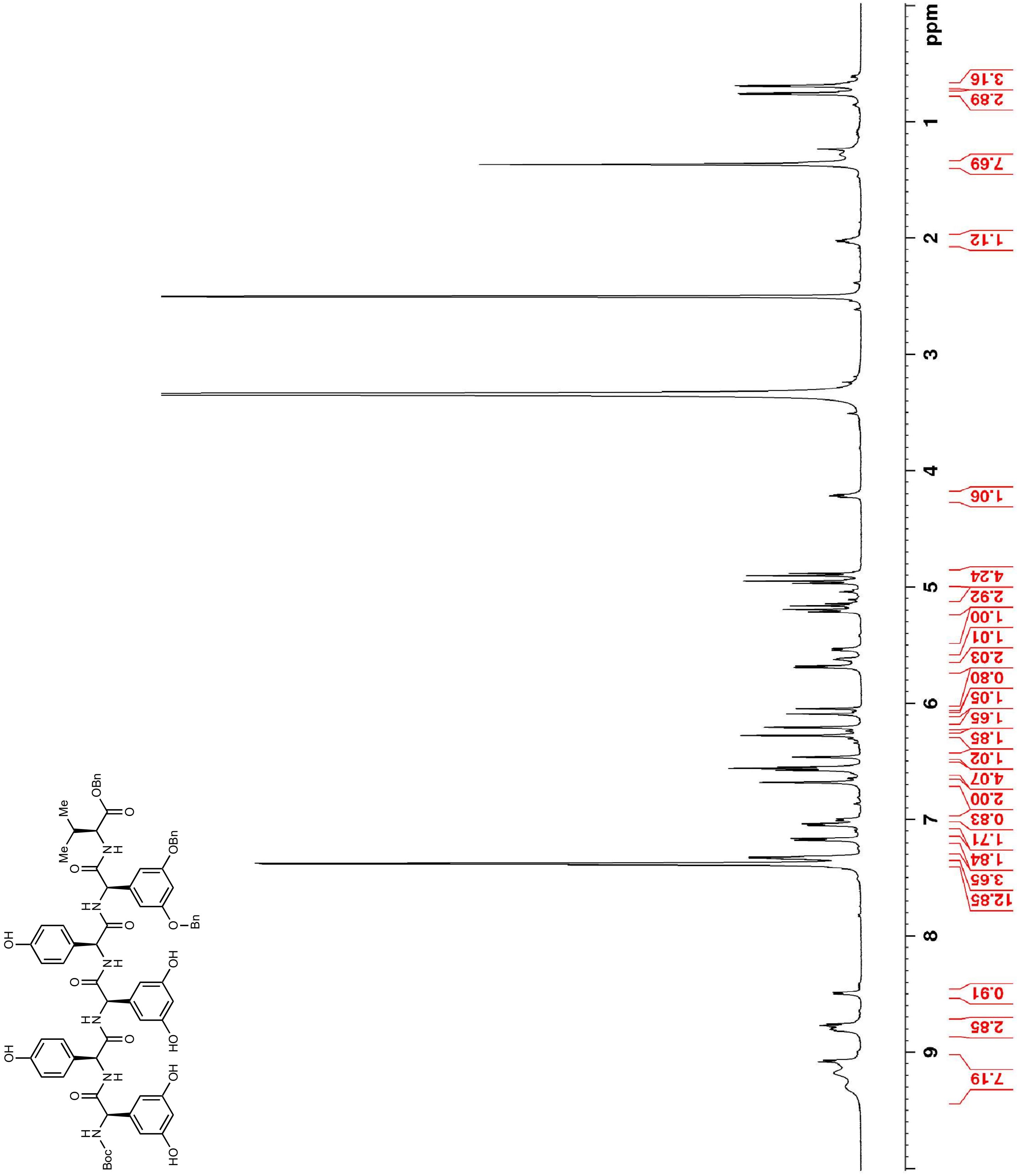

Figure S16. ^{13}C NMR (150 MHz, DMSO-d_6) of **12**

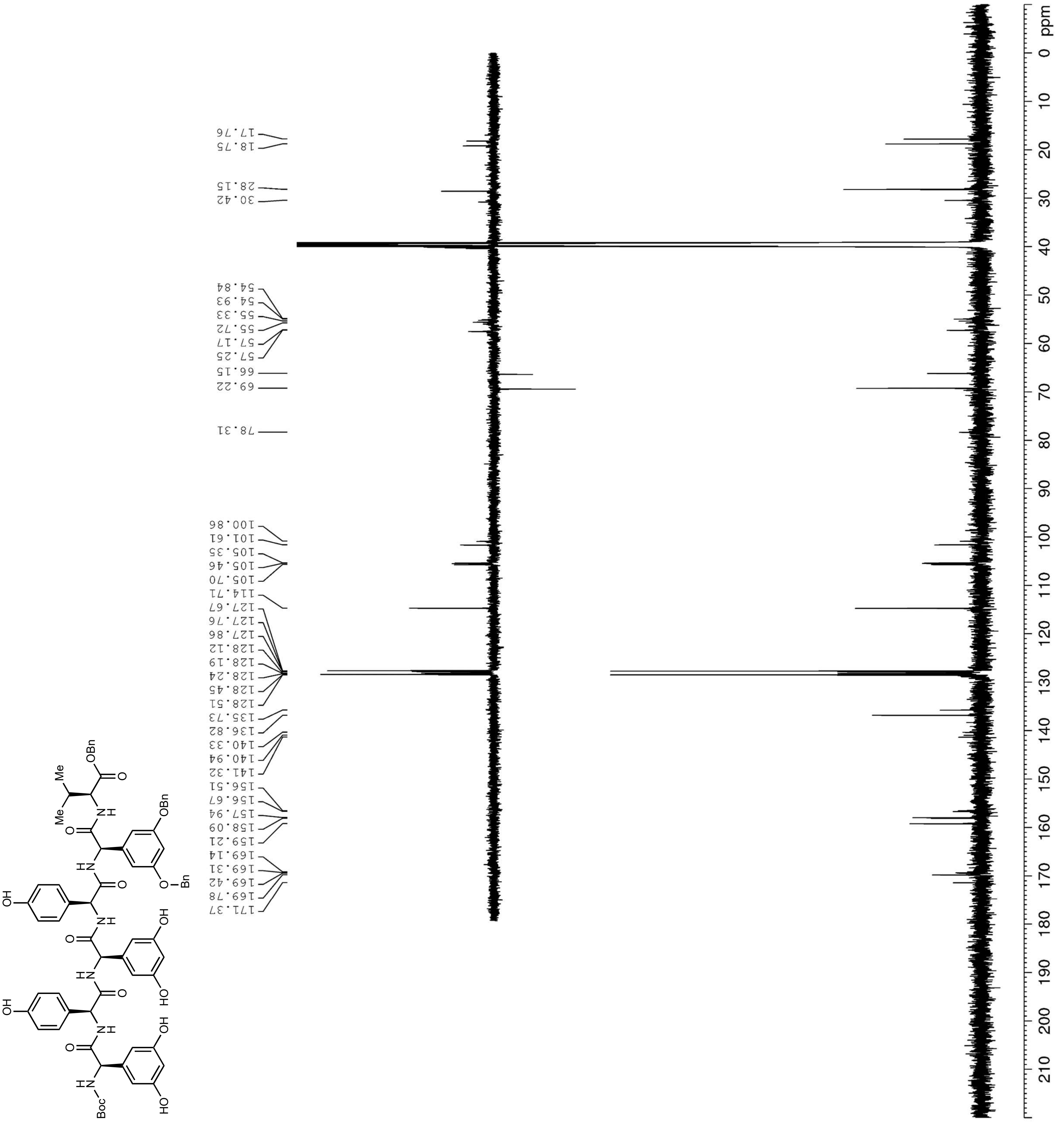

Figure S17. ^{1}H NMR (600 MHz, DMSO-d_6) of **13**

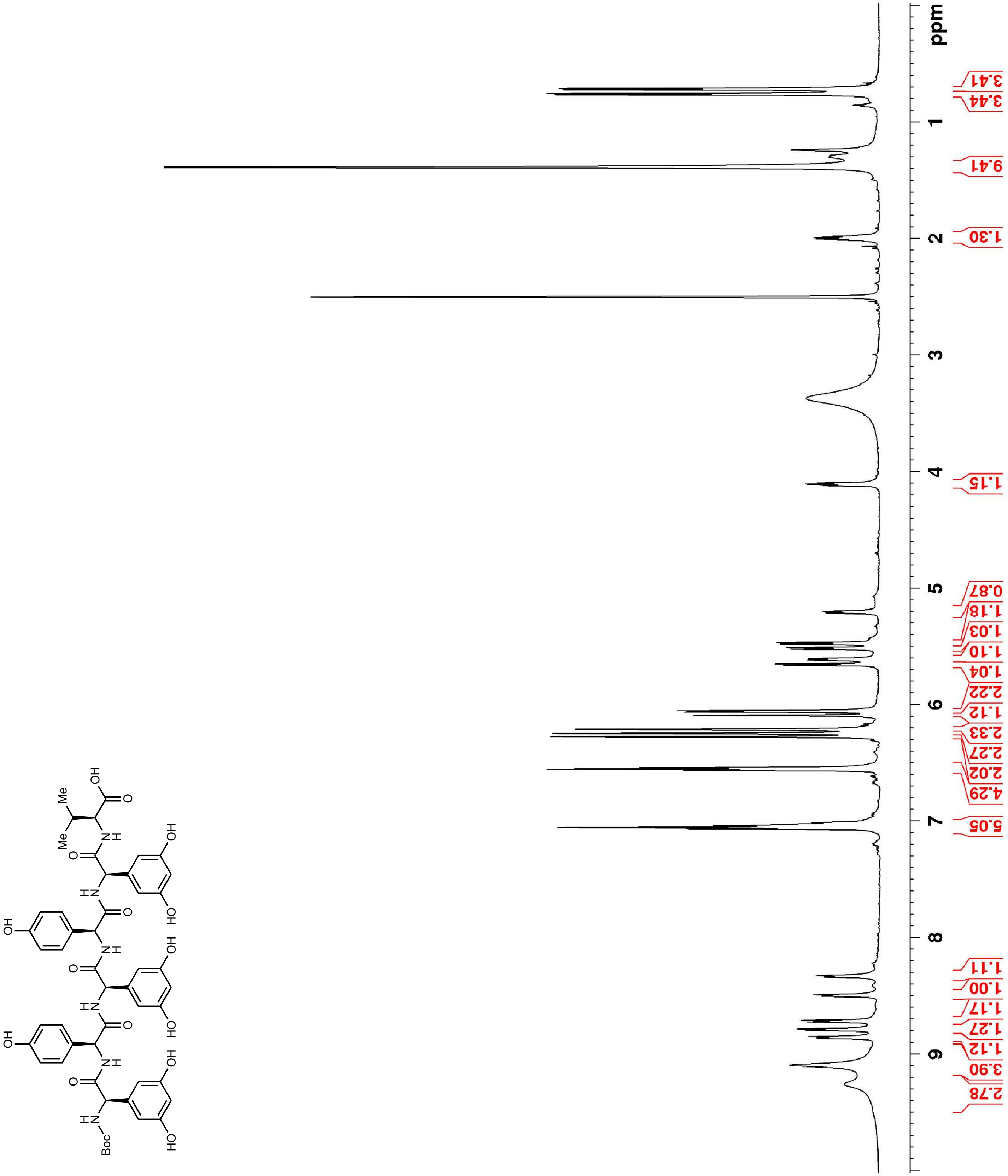

Figure S18. ^{13}C NMR (150 MHz, DMSO-d_6) of **13**

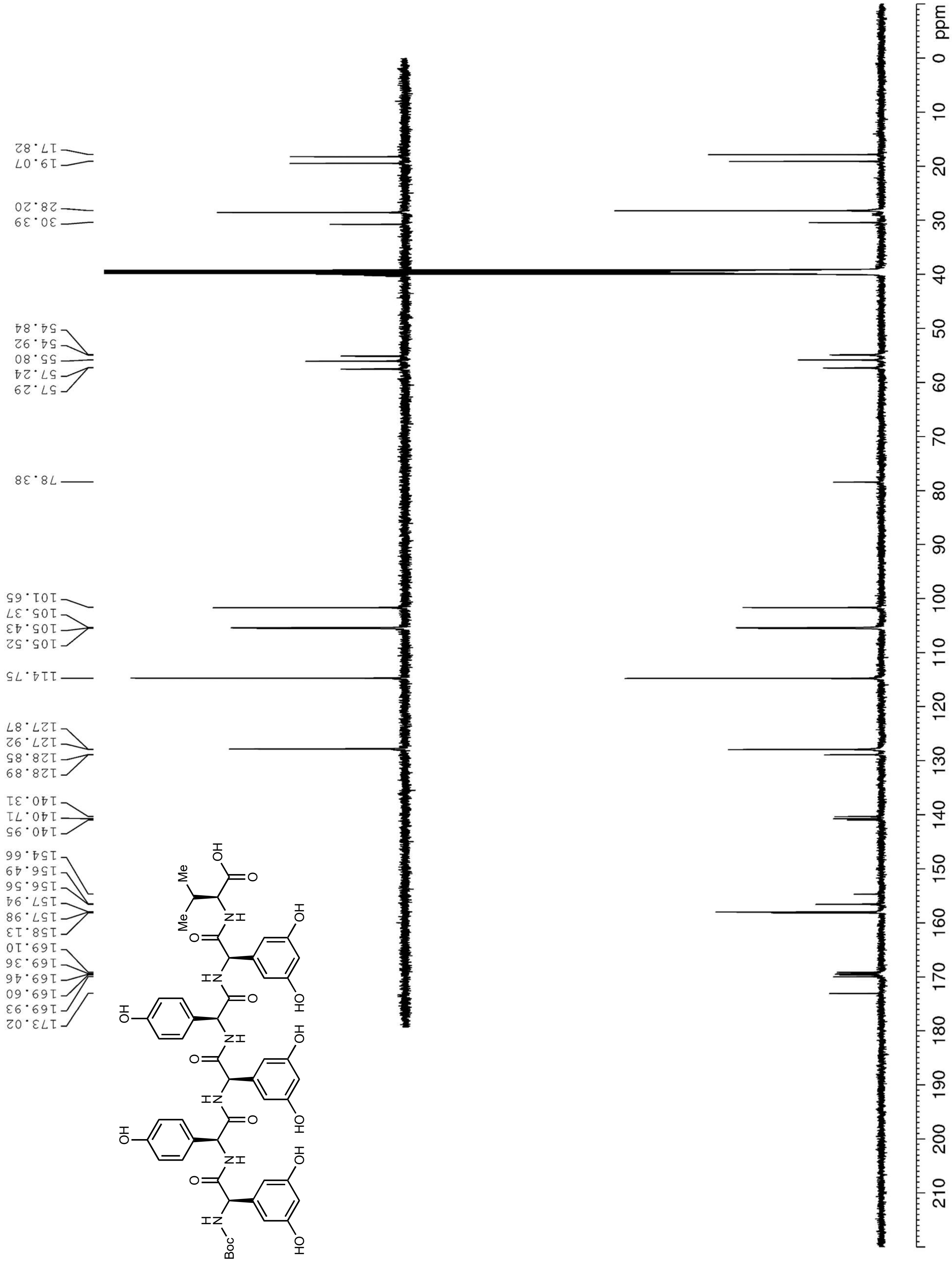

Figure S19. ^{1}H NMR (600 MHz, DMSO-d_6) of **14**

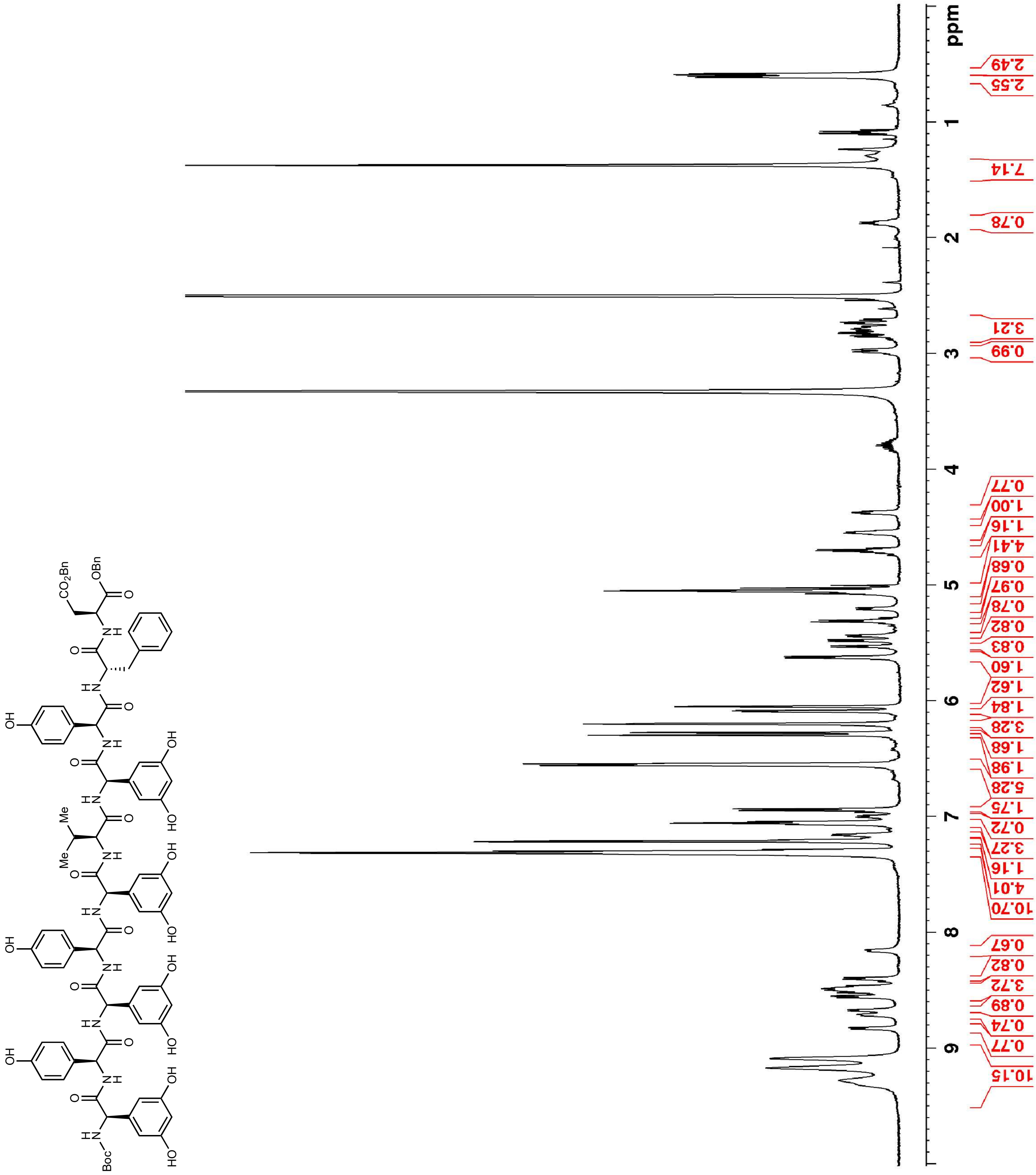

Figure S20. ^{13}C NMR (150 MHz, DMSO-d_6) of **14**

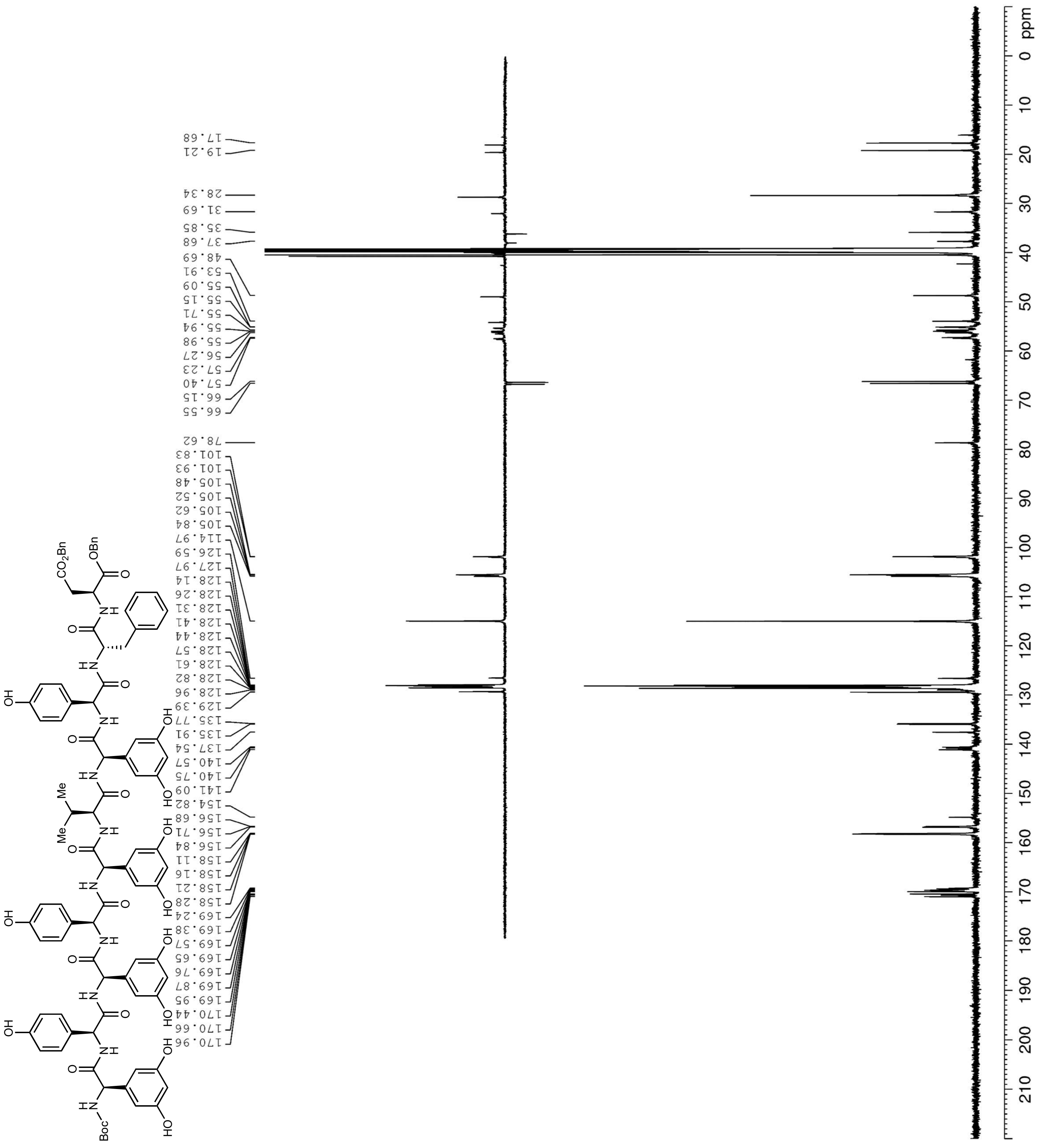

Figure S21. ^{1}H NMR (600 MHz, DMSO-d_6) of **16**

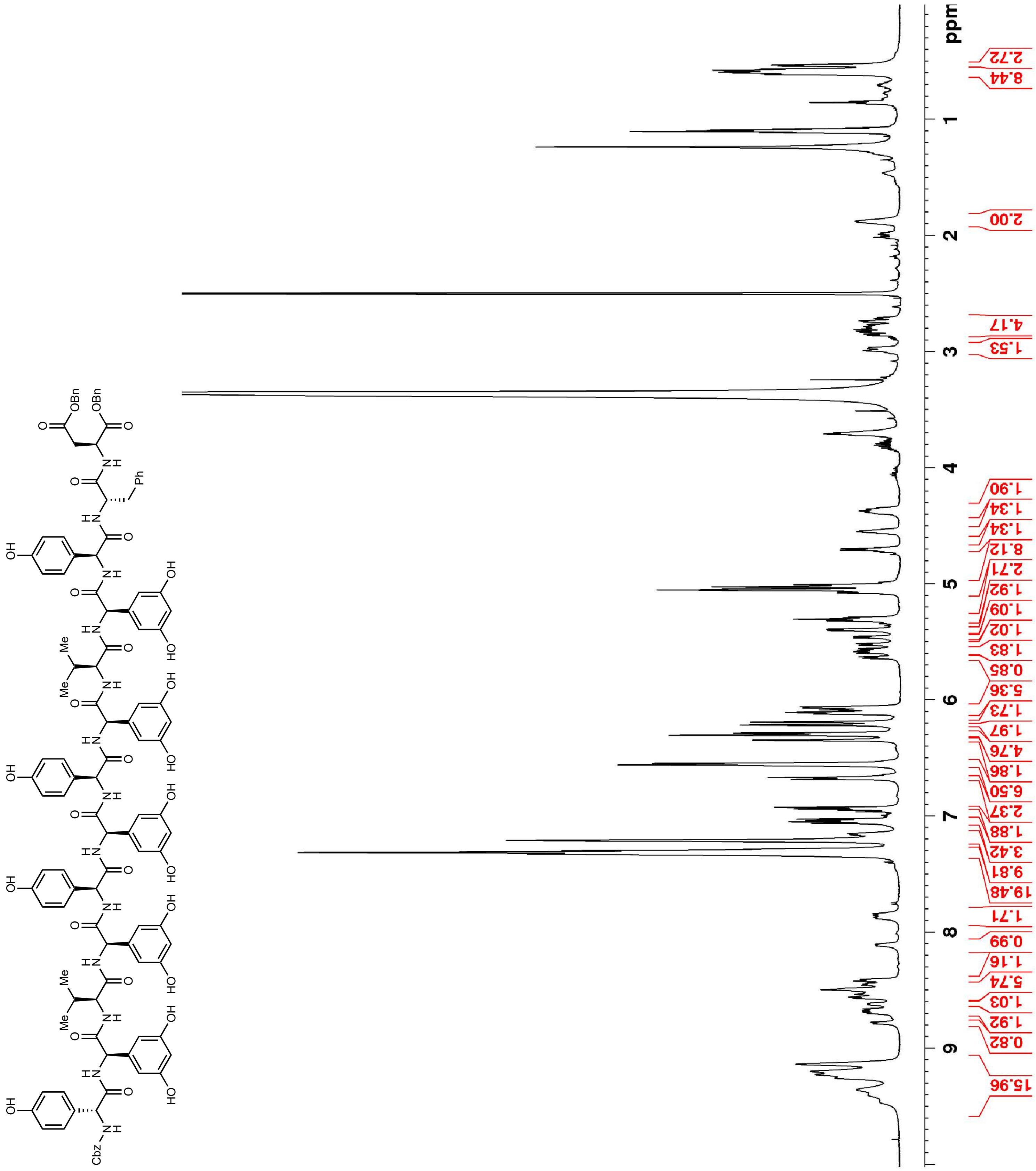

Figure S22. ^{13}C NMR (150 MHz, DMSO-d_6) of **16**

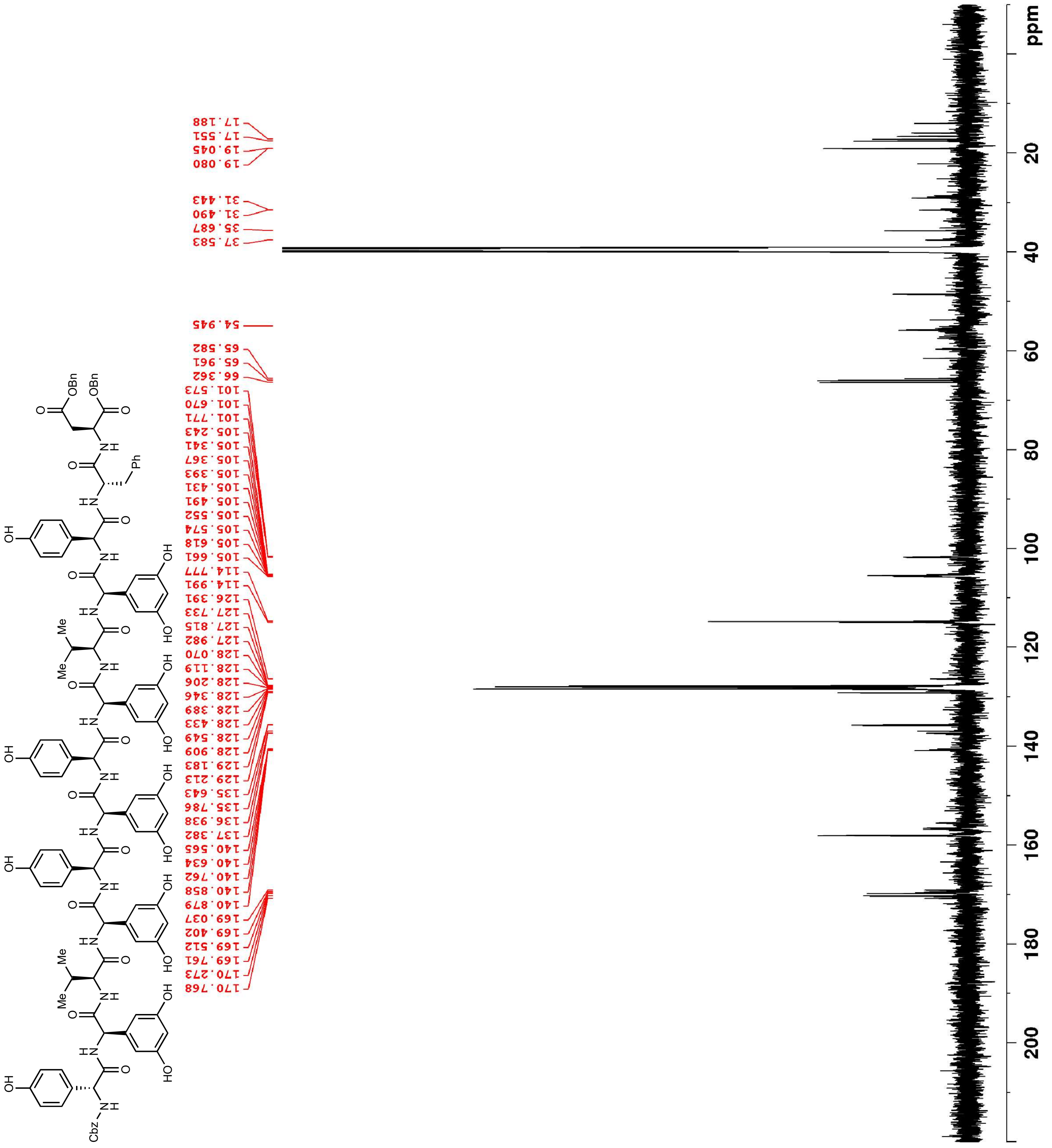

Figure S23. HSQC (600 MHz, DMSO-d_6) of **16**

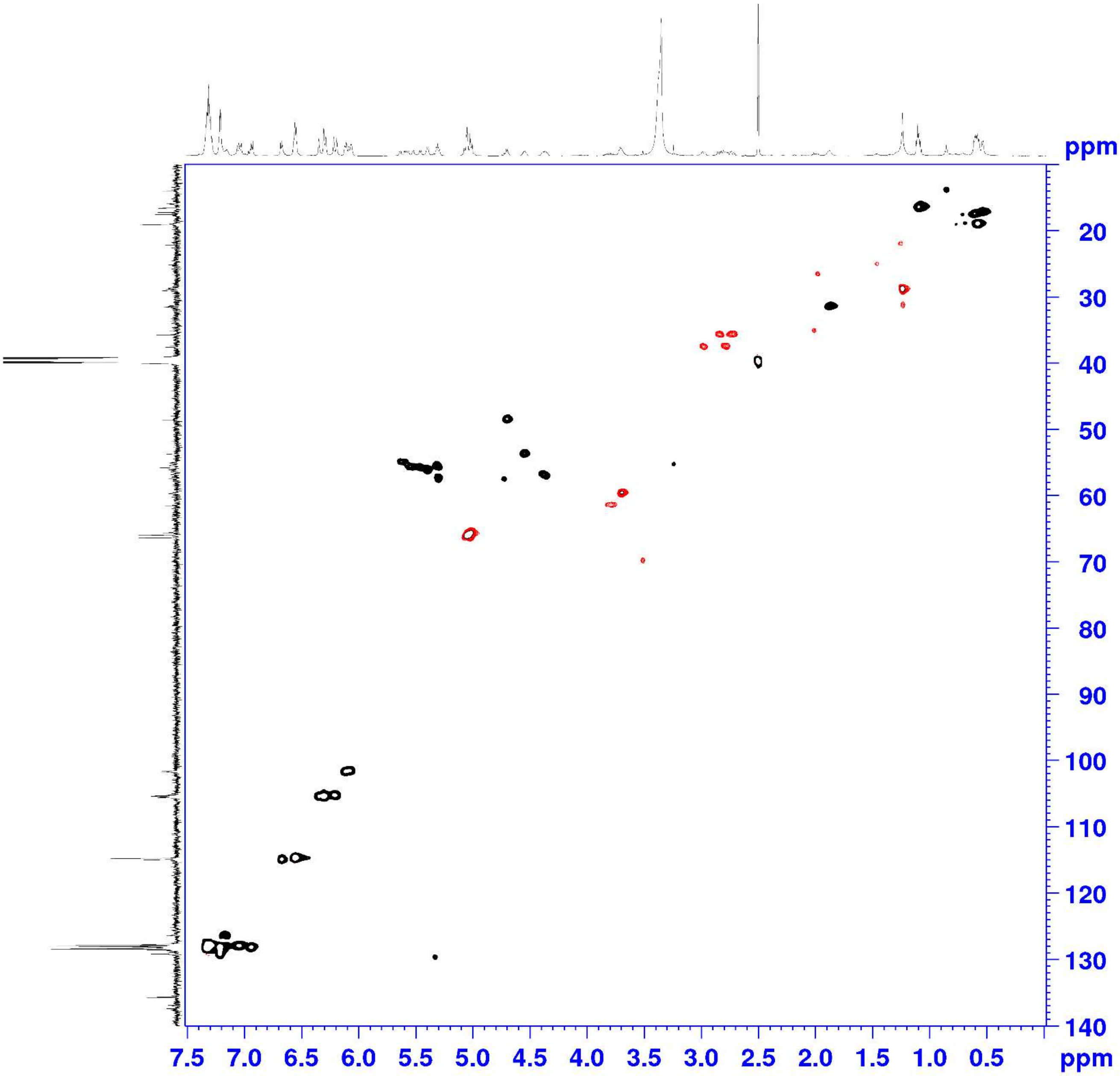

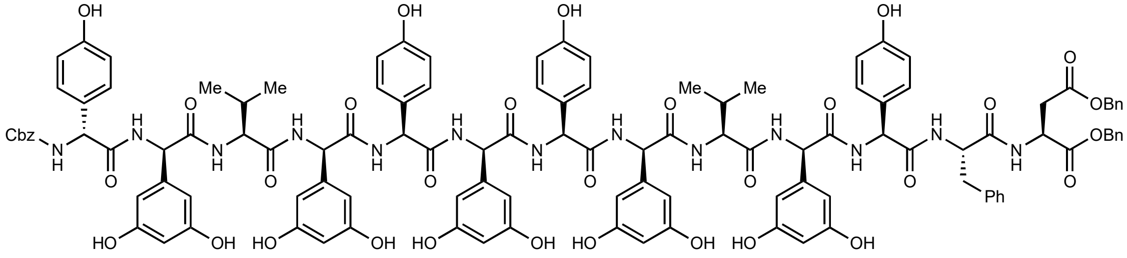

Figure S24. Crude ^{1}H NMR (600 MHz, DMSO-d_6) of **17**

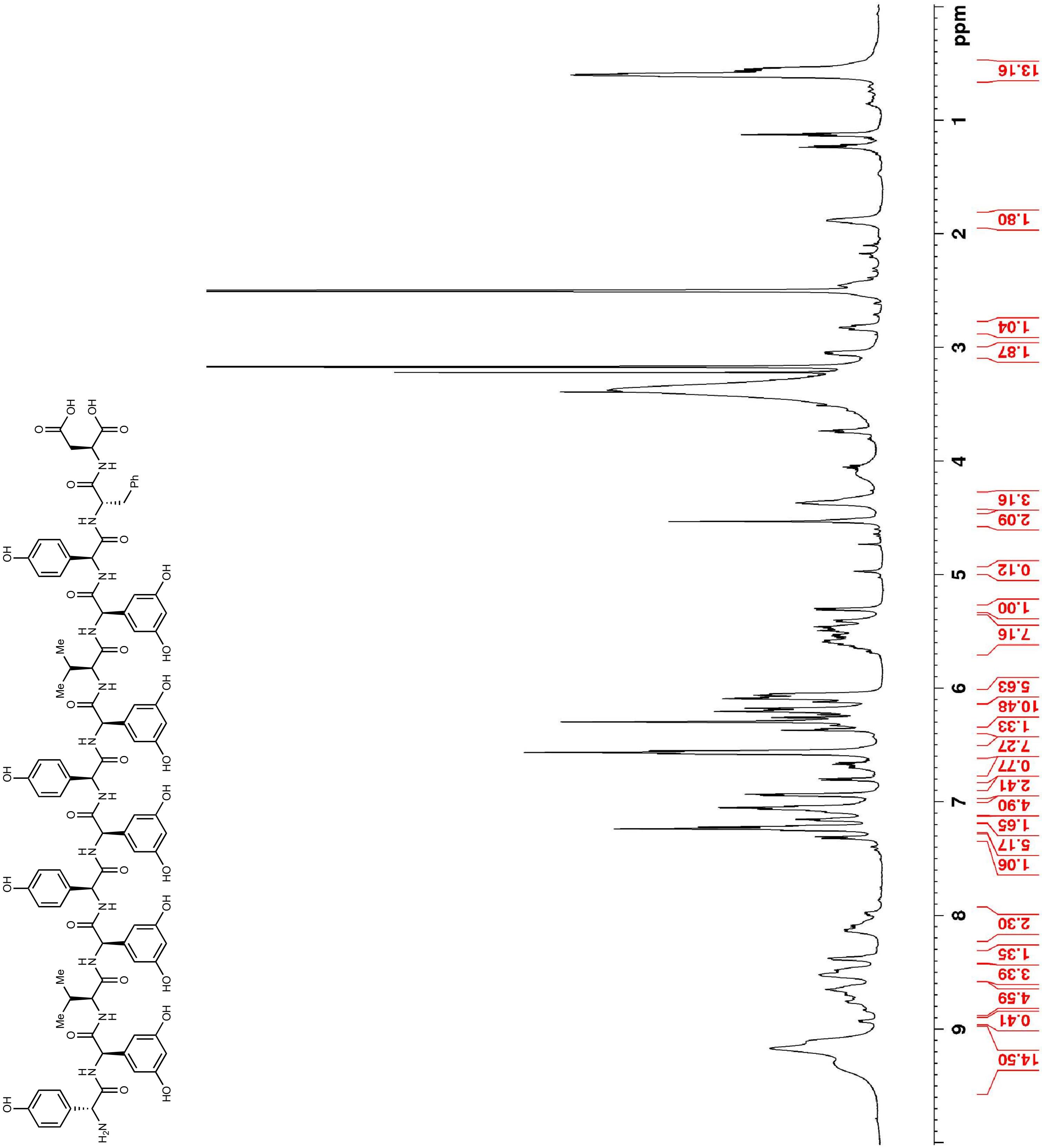

Figure S25. ^{1}H NMR (900 MHz, DMSO-d_6) of **17**

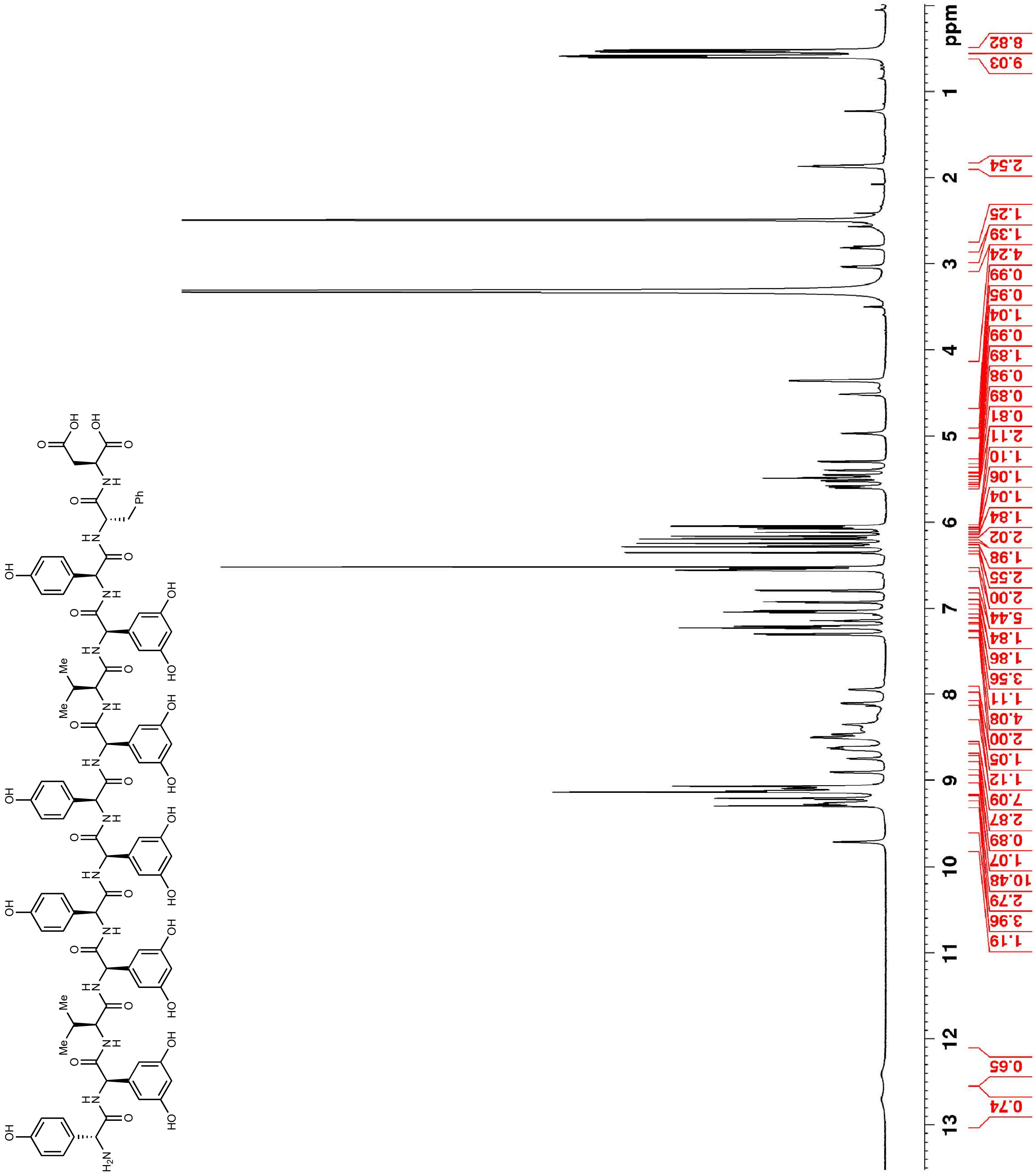

Figure S26. ^{13}C NMR (225 MHz, DMSO-d_6) of **17**

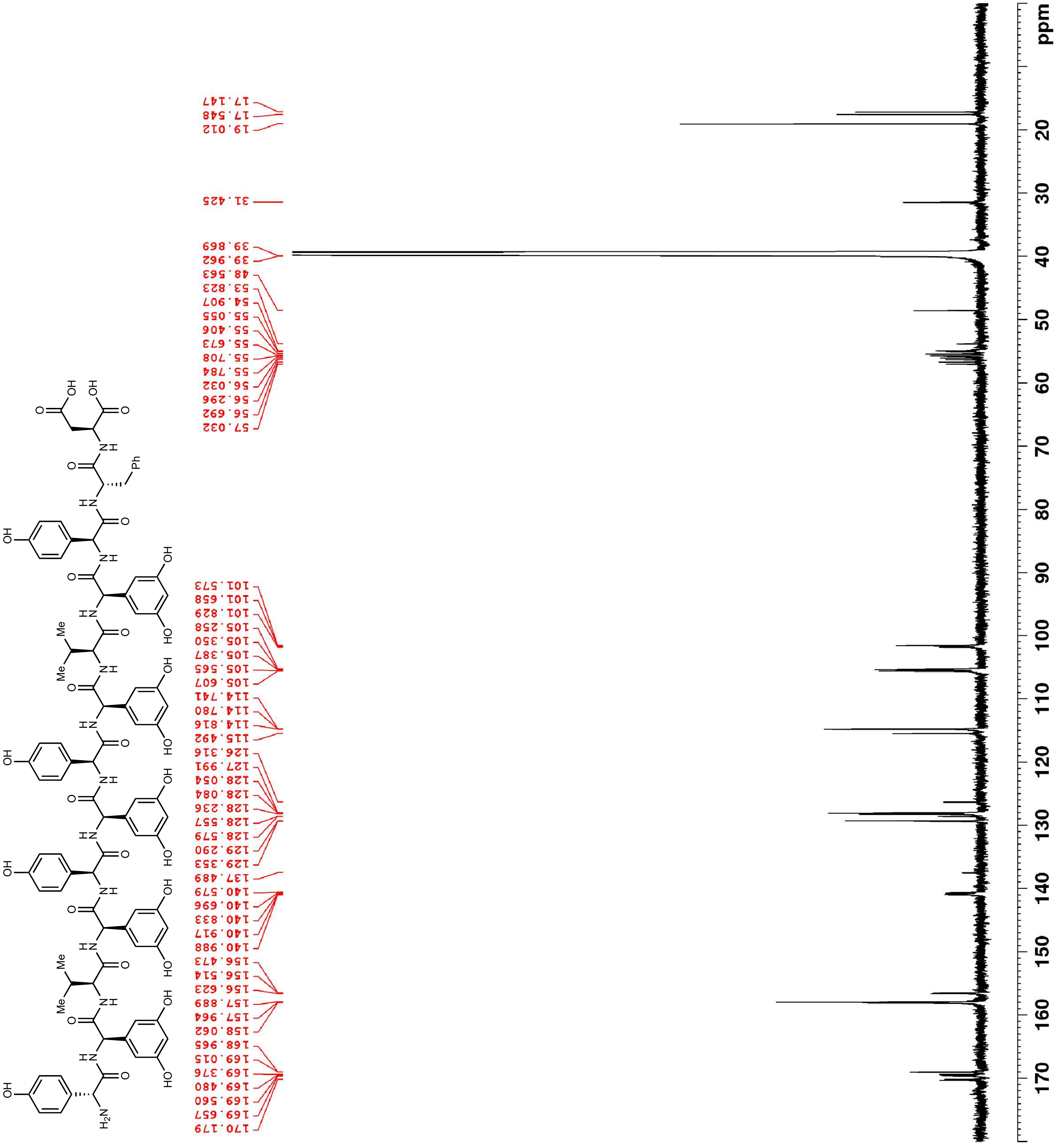

Figure S27. HSQC (900 MHz, DMSO-d_6) of **17**

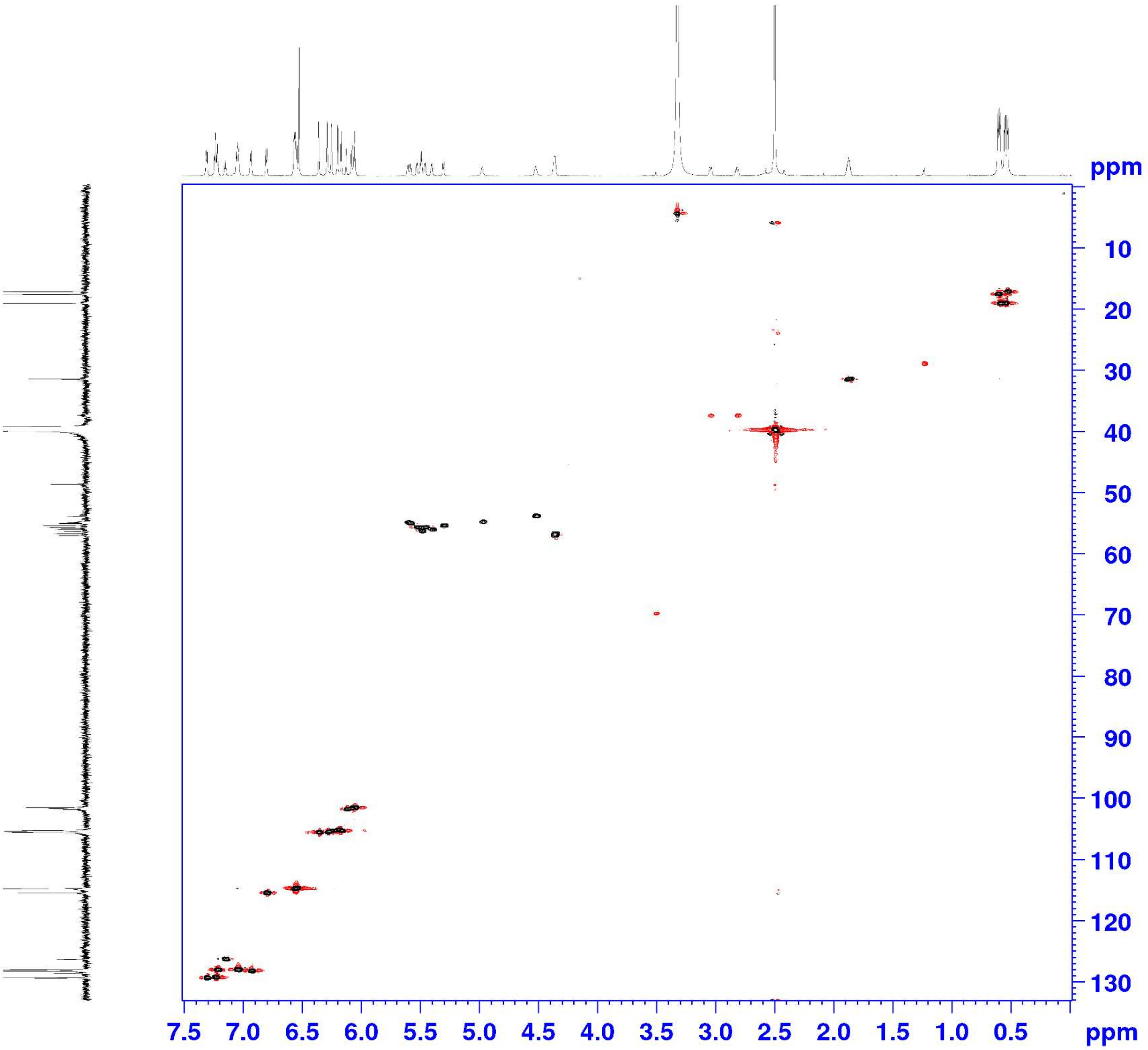

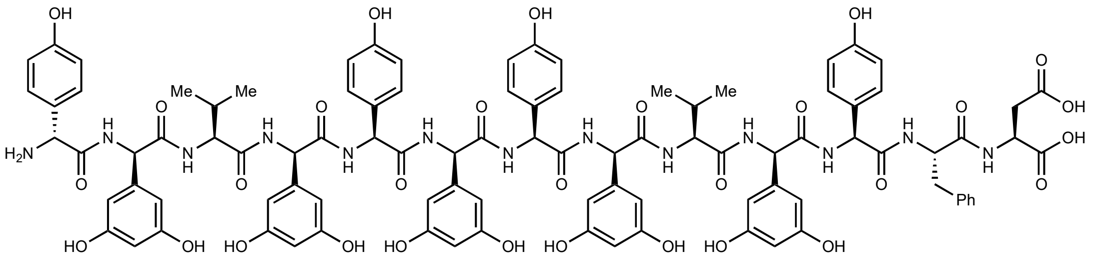

Figure S28. ^{1}H NMR (400 MHz, $CDCl_3$) of **18**

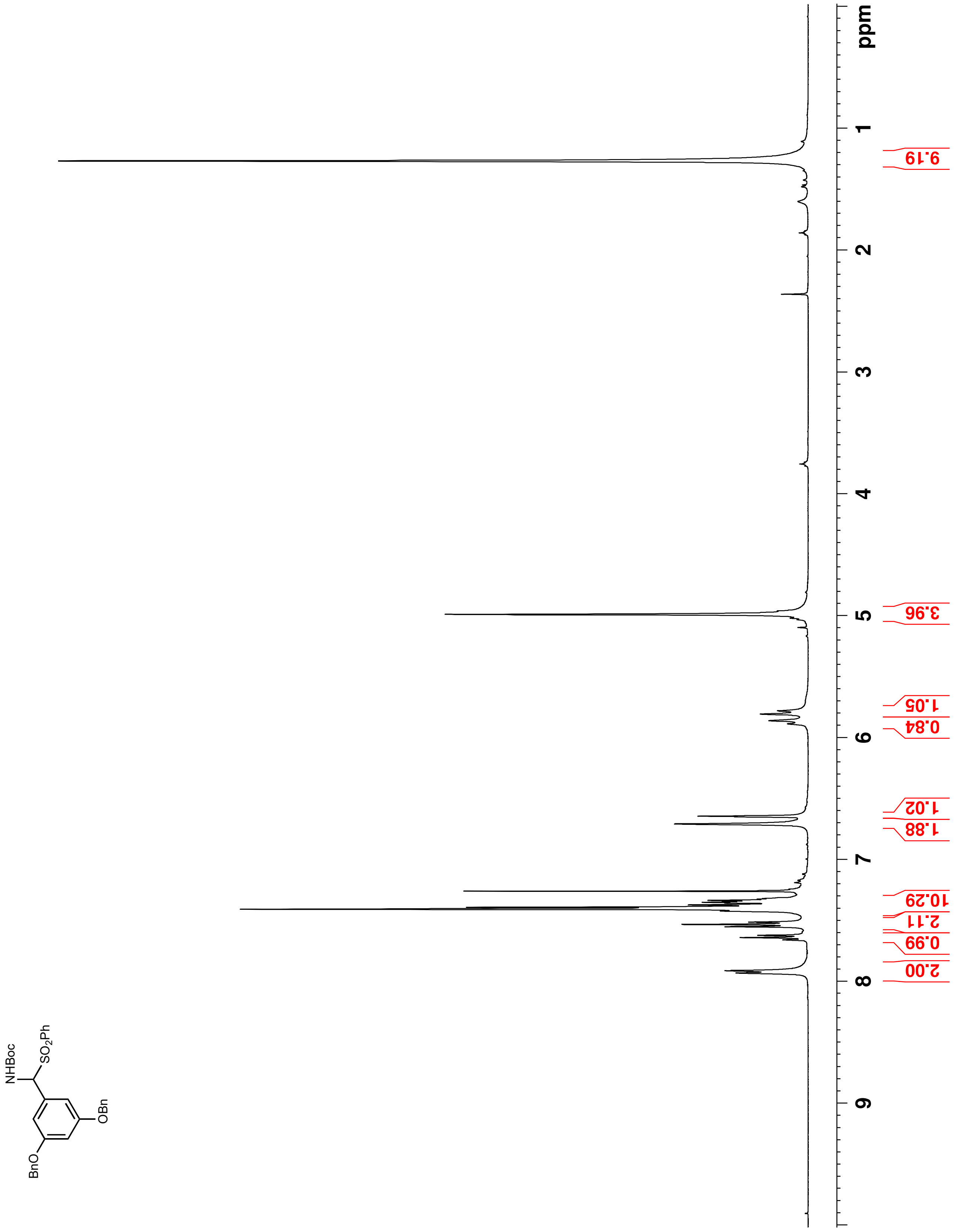

Figure S29. ^{13}C NMR (100 MHz, $CDCl_3$) of **18**

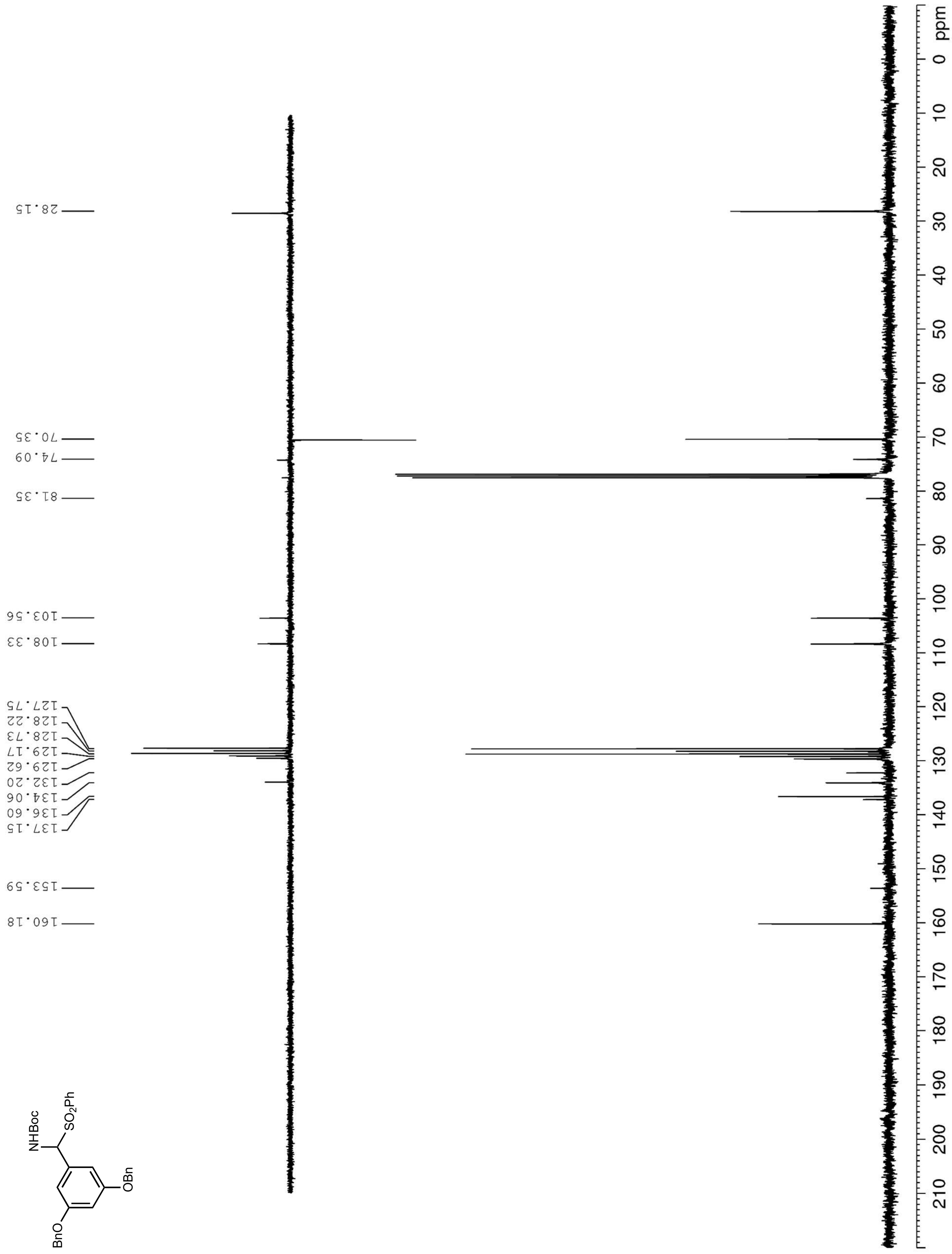

Figure S30. ^{1}H NMR (400 MHz, DMSO-d_6) of **20**

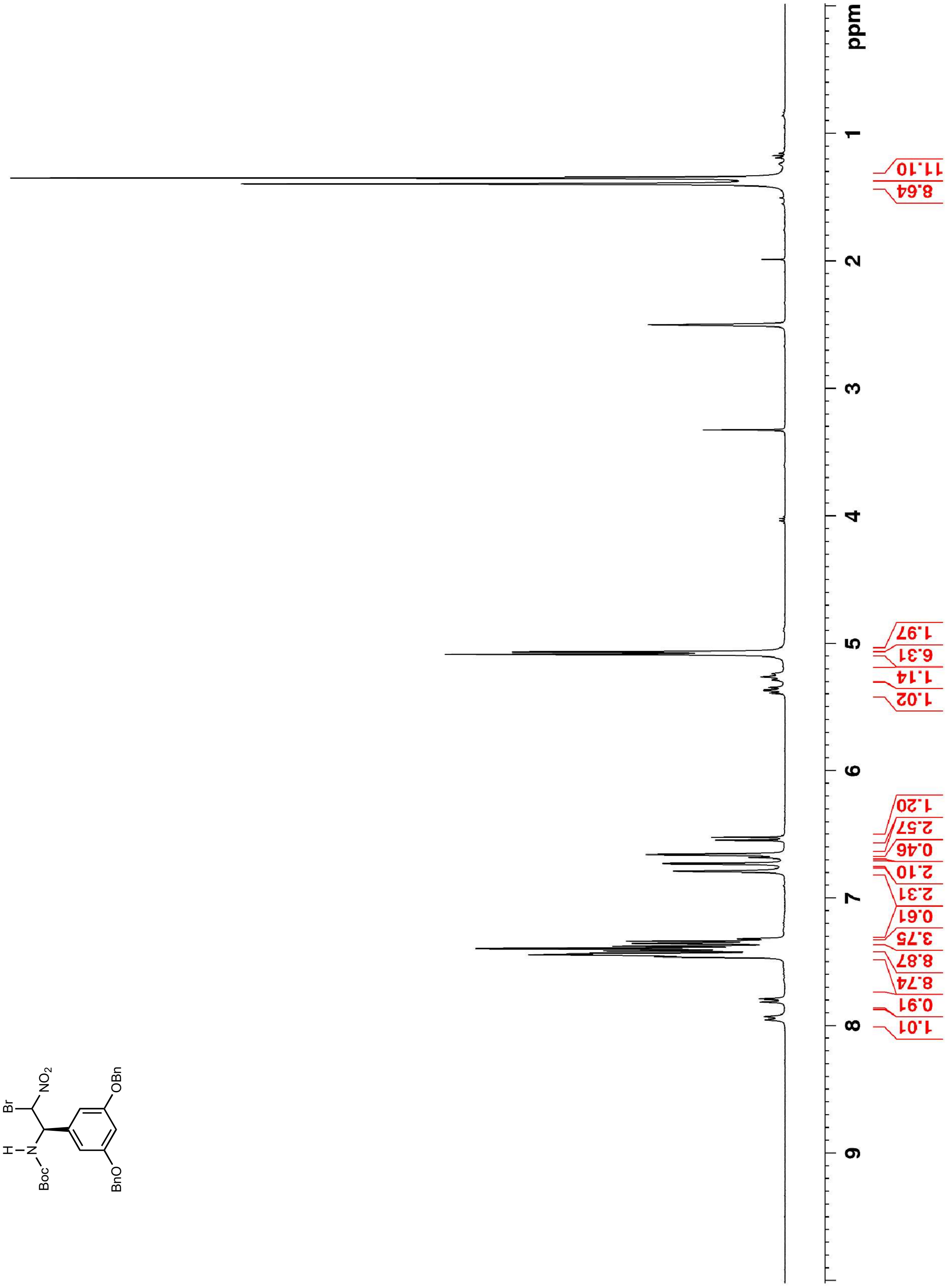

Figure S31. ^{13}C NMR (100 MHz, DMSO-d_6) of **20**

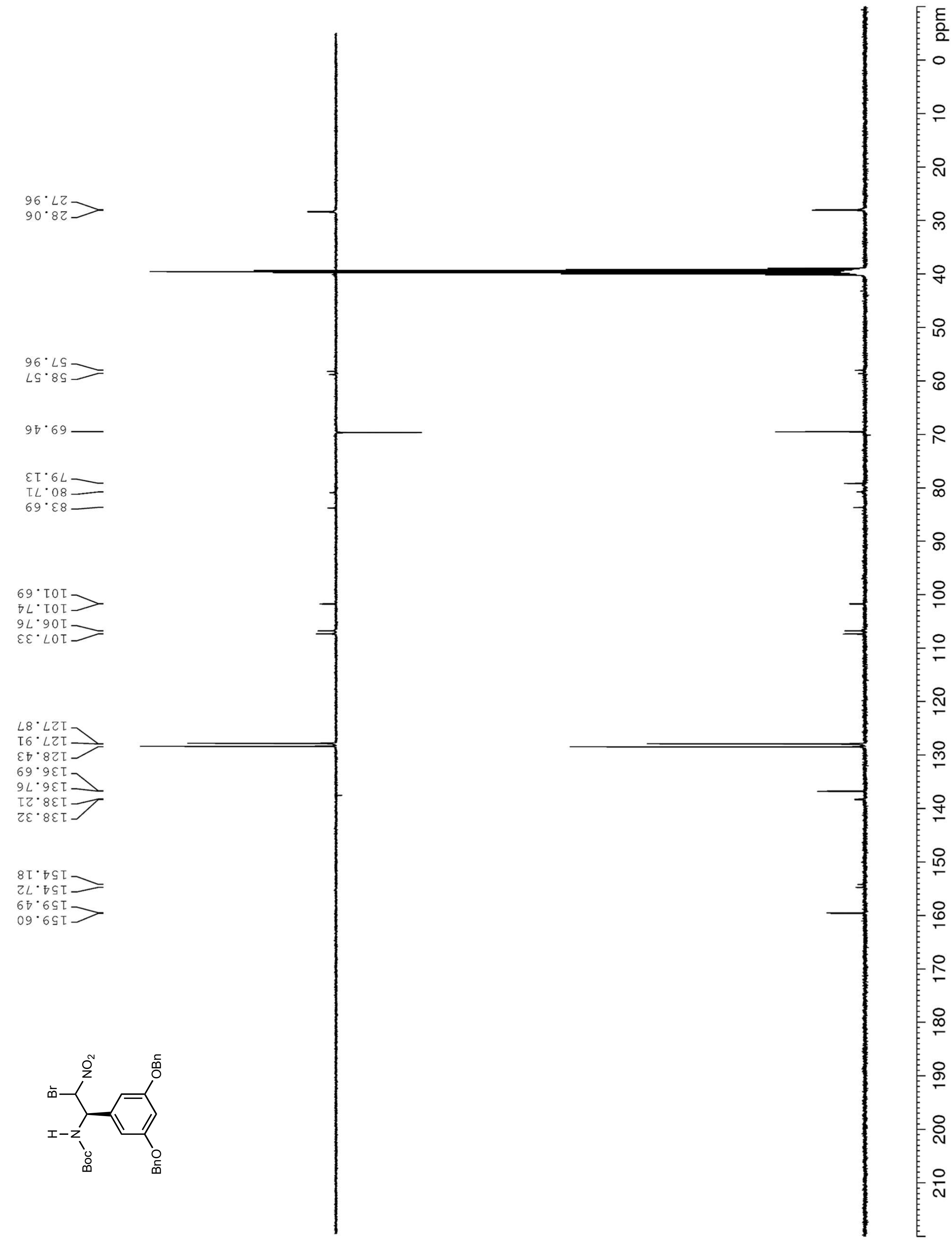

Figure S32. ^{1}H NMR (600 MHz, $CDCl_3$) of **21**

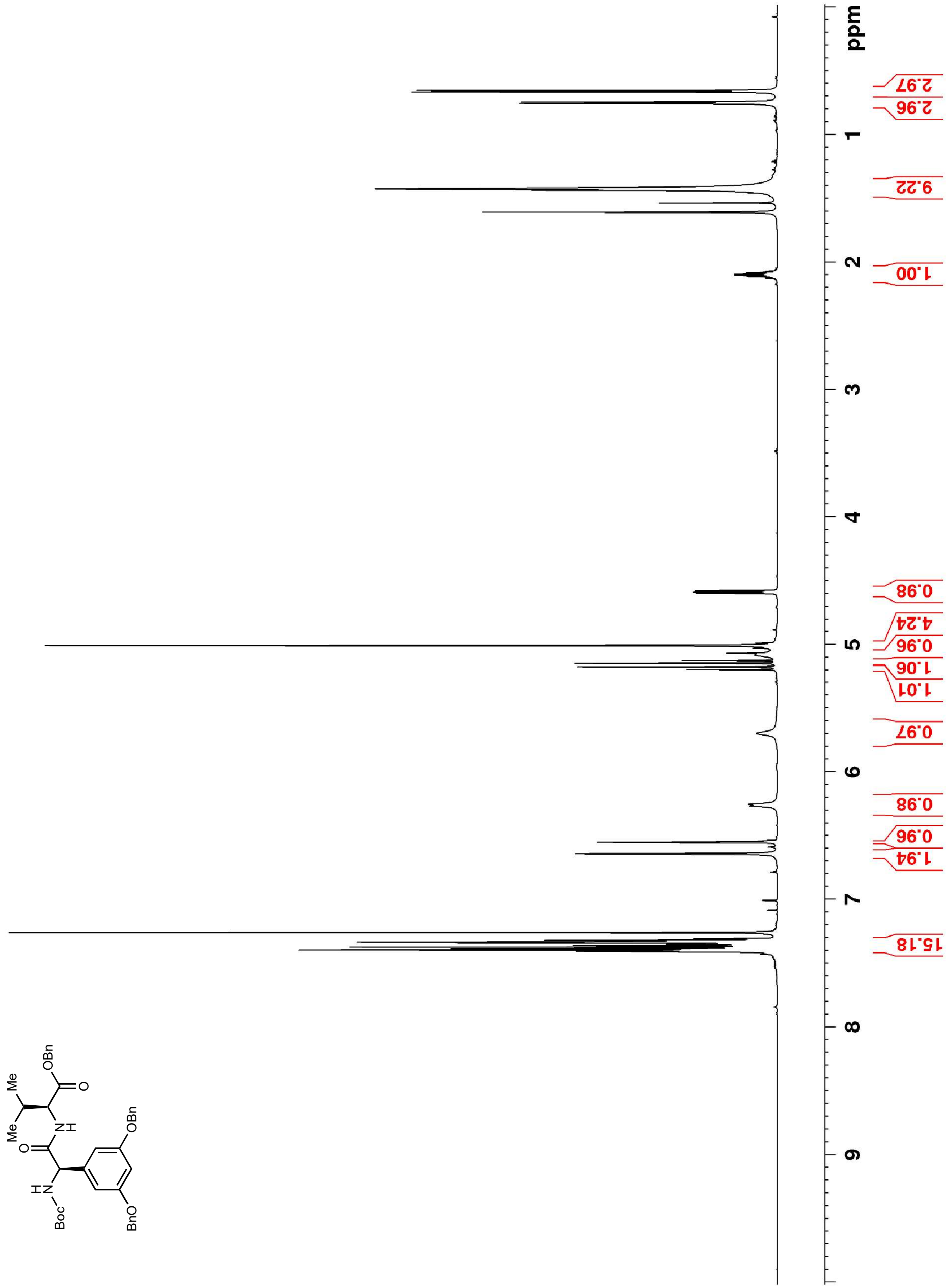

Figure S33. ^{13}C NMR (150 MHz, $CDCl_3$) of **21**

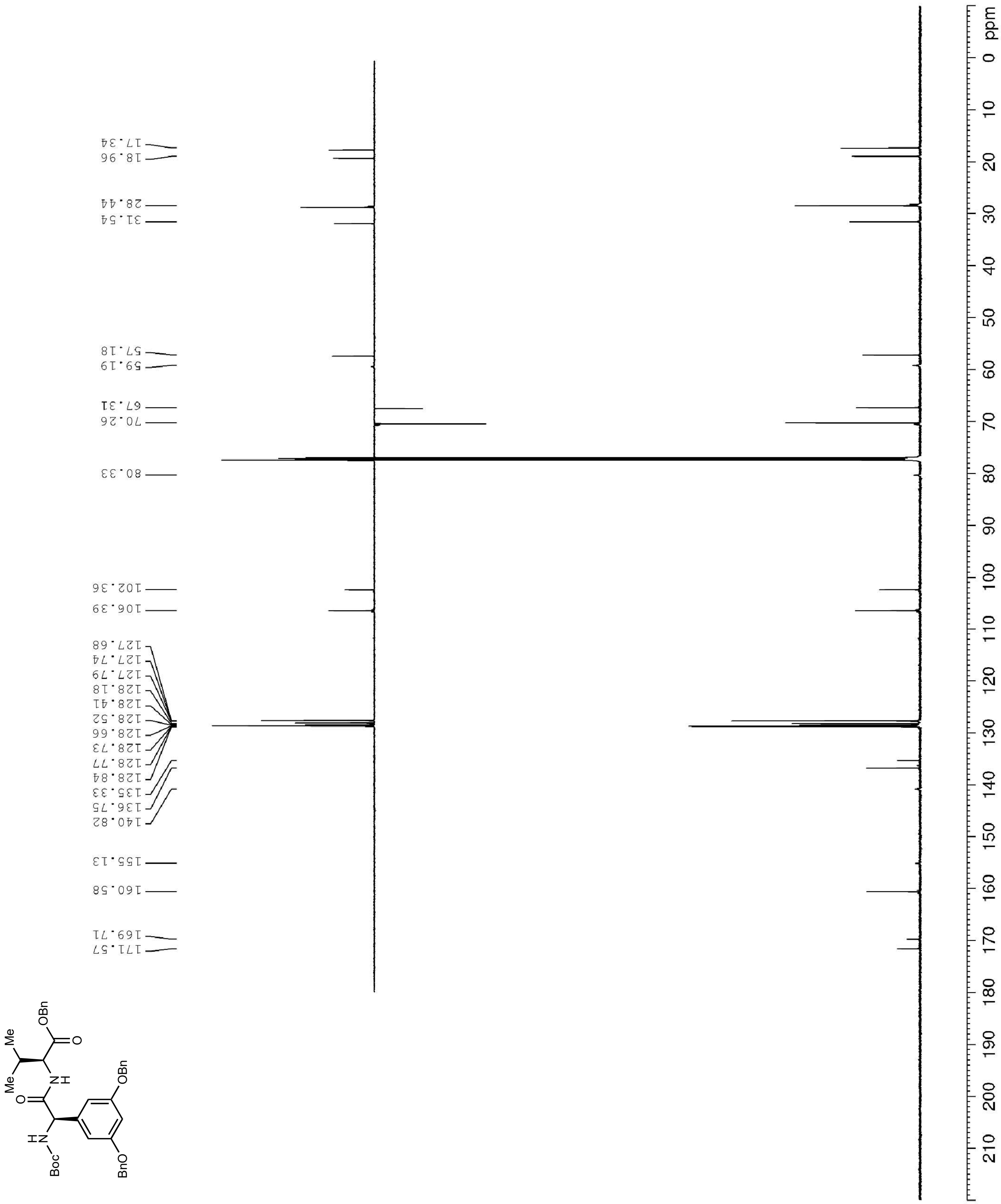

Figure S34. ^{1}H NMR (400 MHz, DMSO-d_6) of **23**

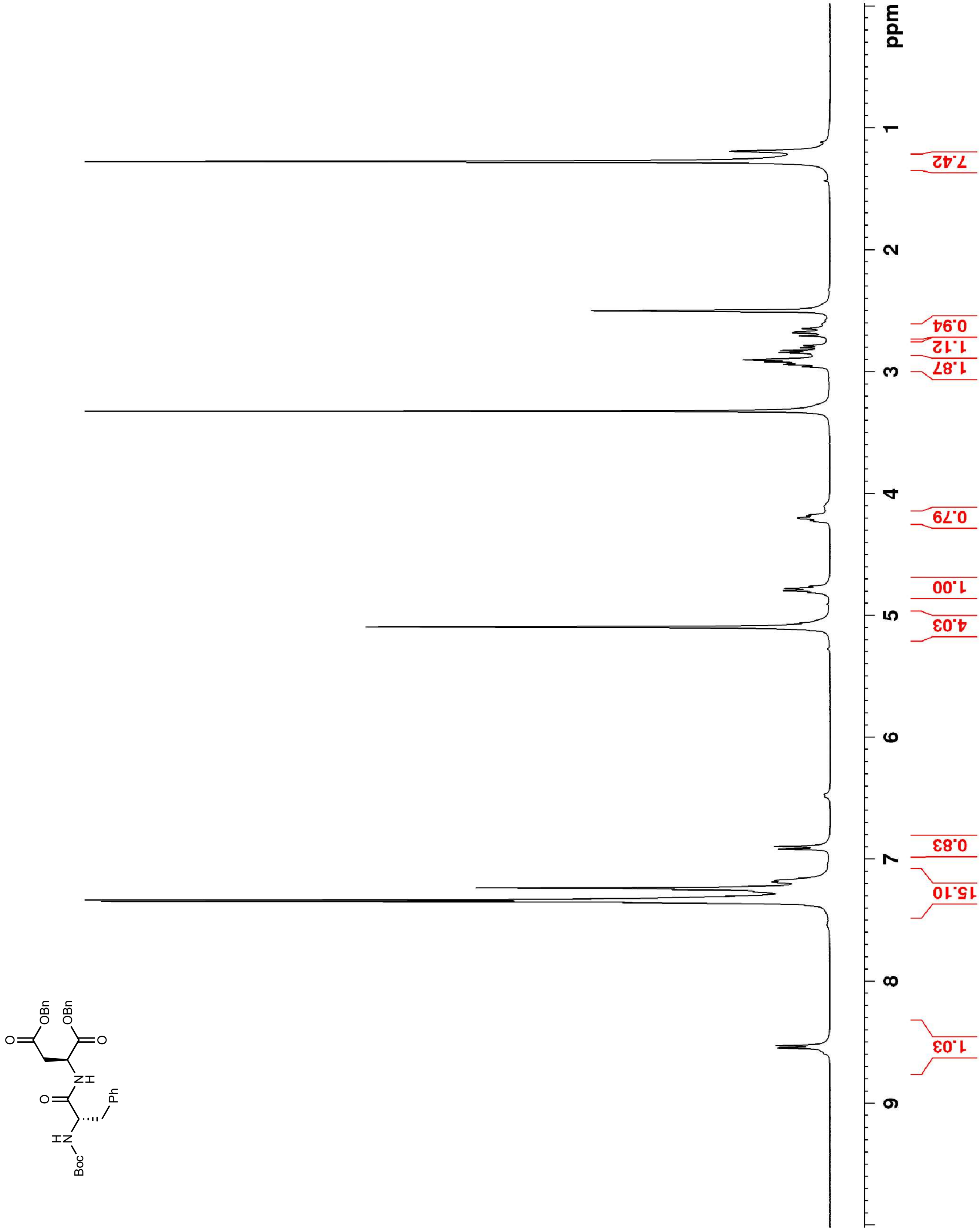

Figure S35. ^{13}C NMR (100 MHz, DMSO-d_6) of **23**

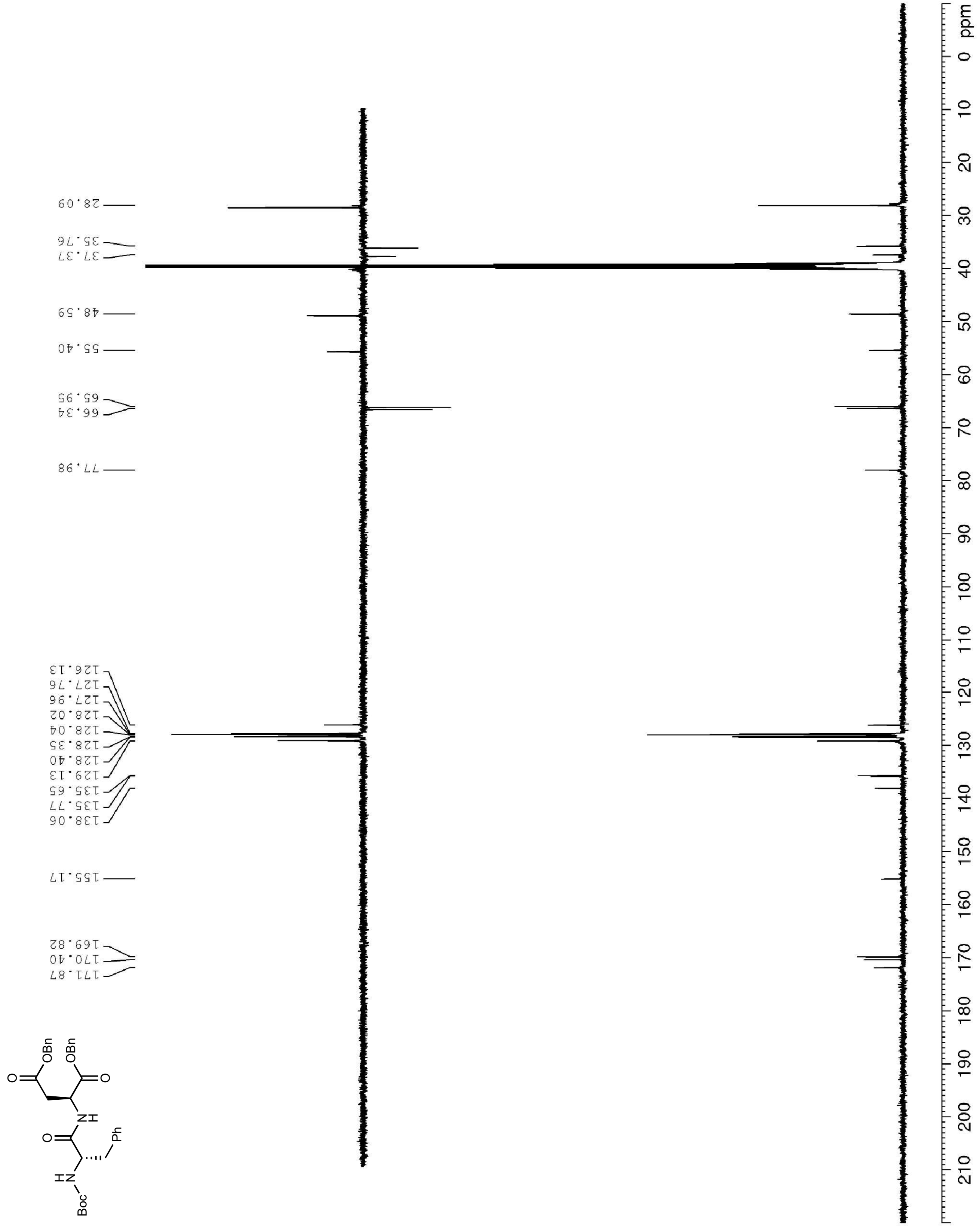

Figure S36. ^{1}H NMR (600 MHz, DMSO-d_6) of **25**

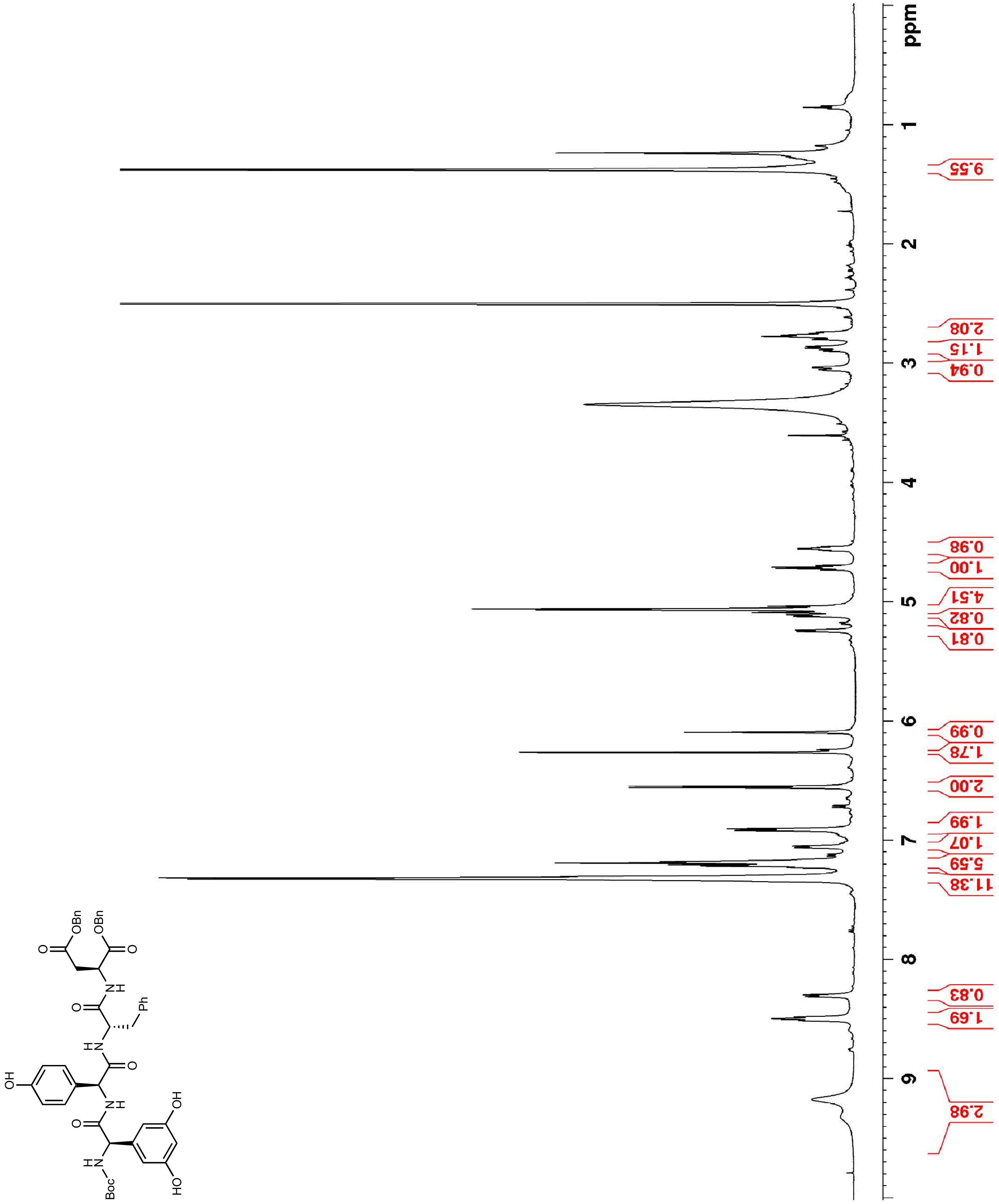

Figure S37. ^{13}C NMR (150 MHz, DMSO-d_6) of **25**

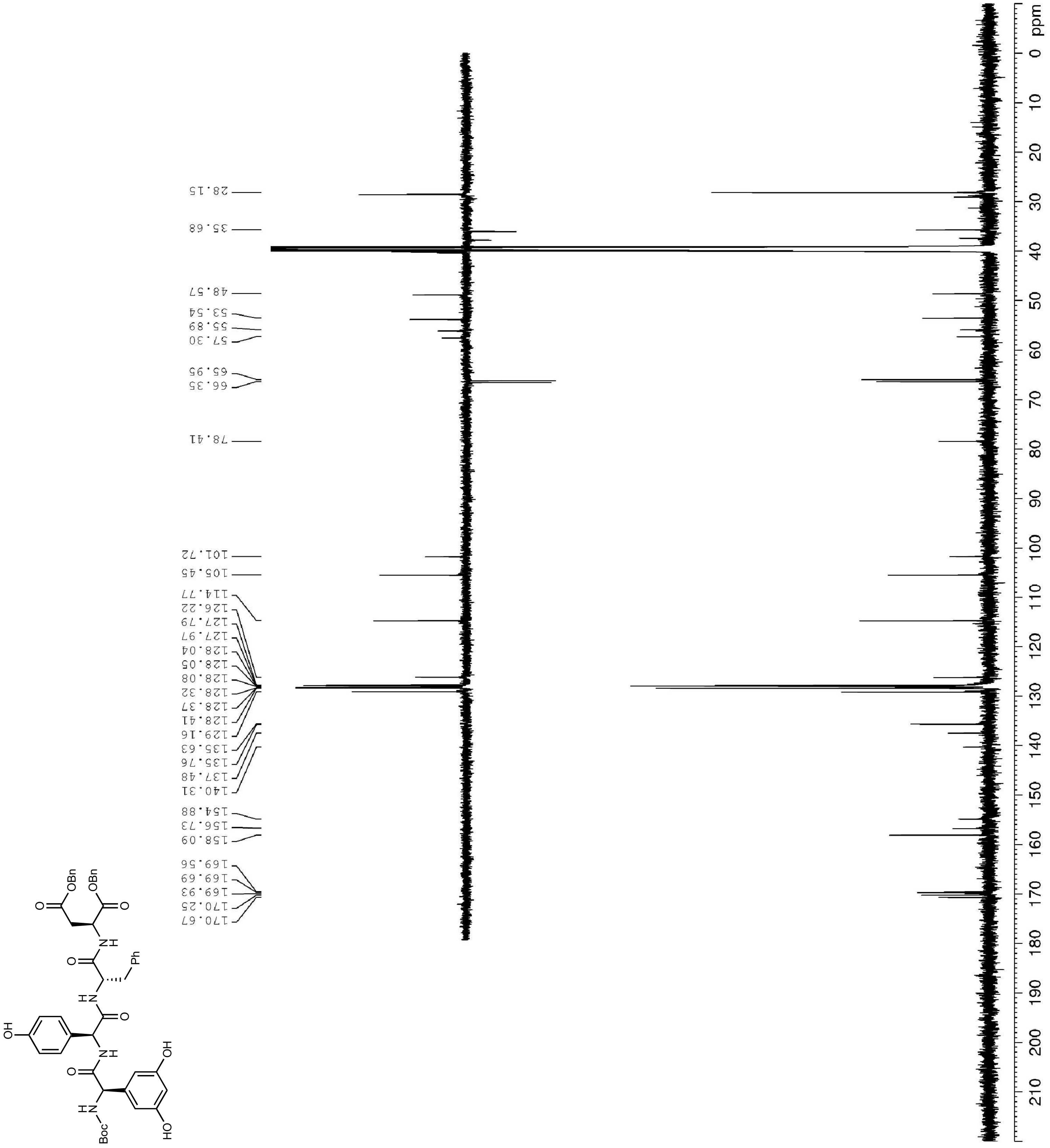

Figure S38. ^{1}H NMR (600 MHz, DMSO-d_6) of **27**

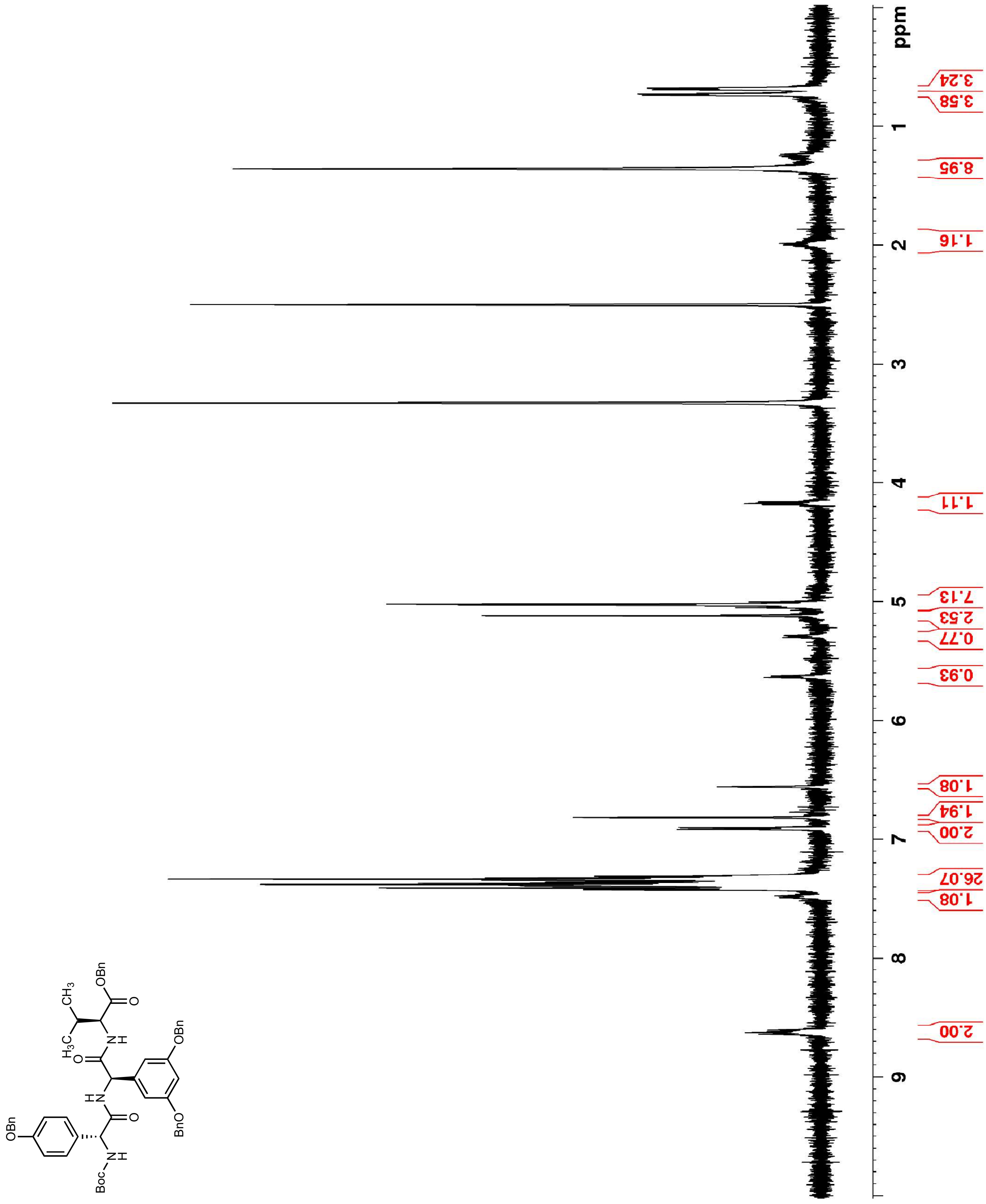

Figure S39. ^{13}C NMR (150 MHz, DMSO-d_6) of **27**

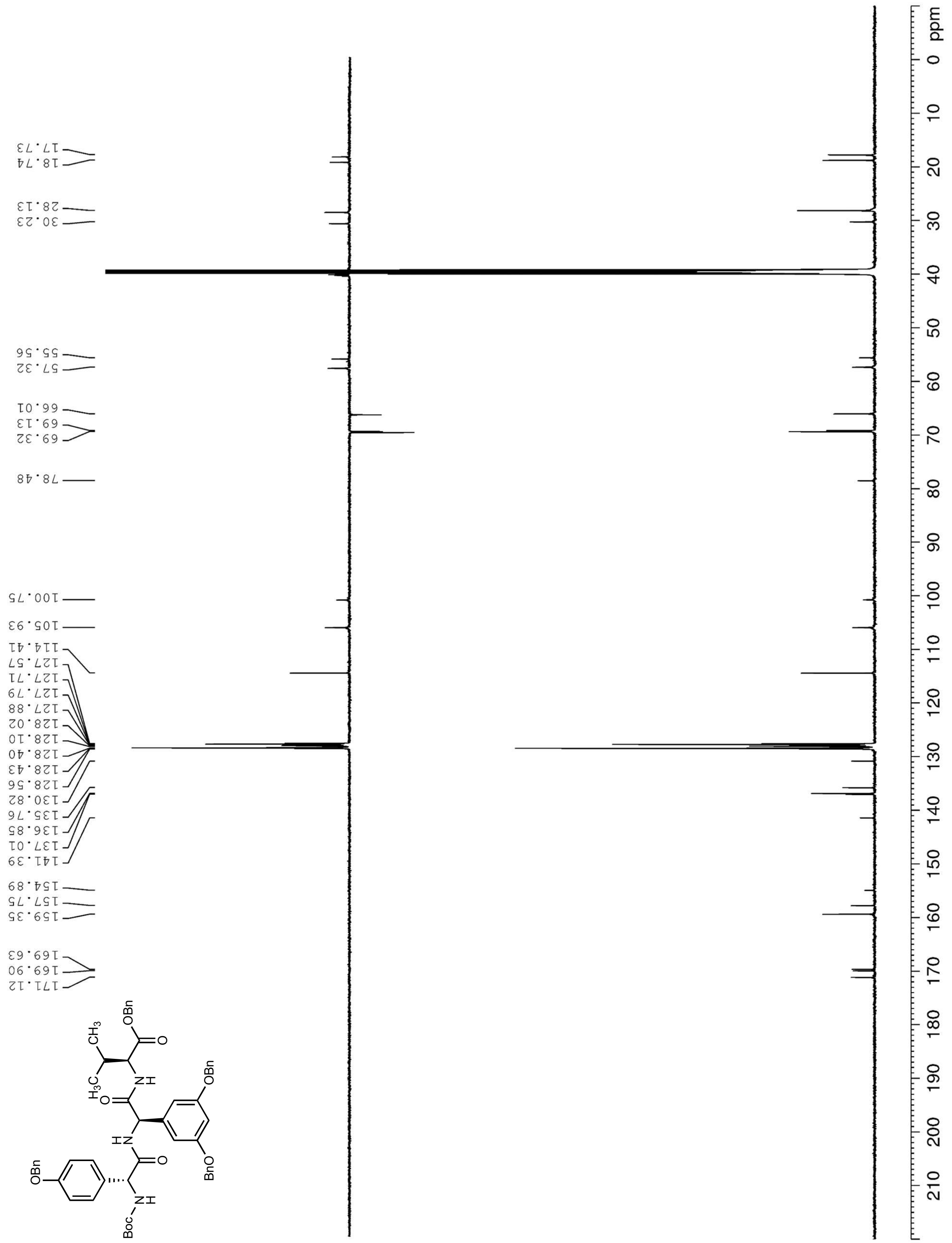

Figure S40. ^{1}H NMR (600 MHz, DMSO-d_6) of **28**

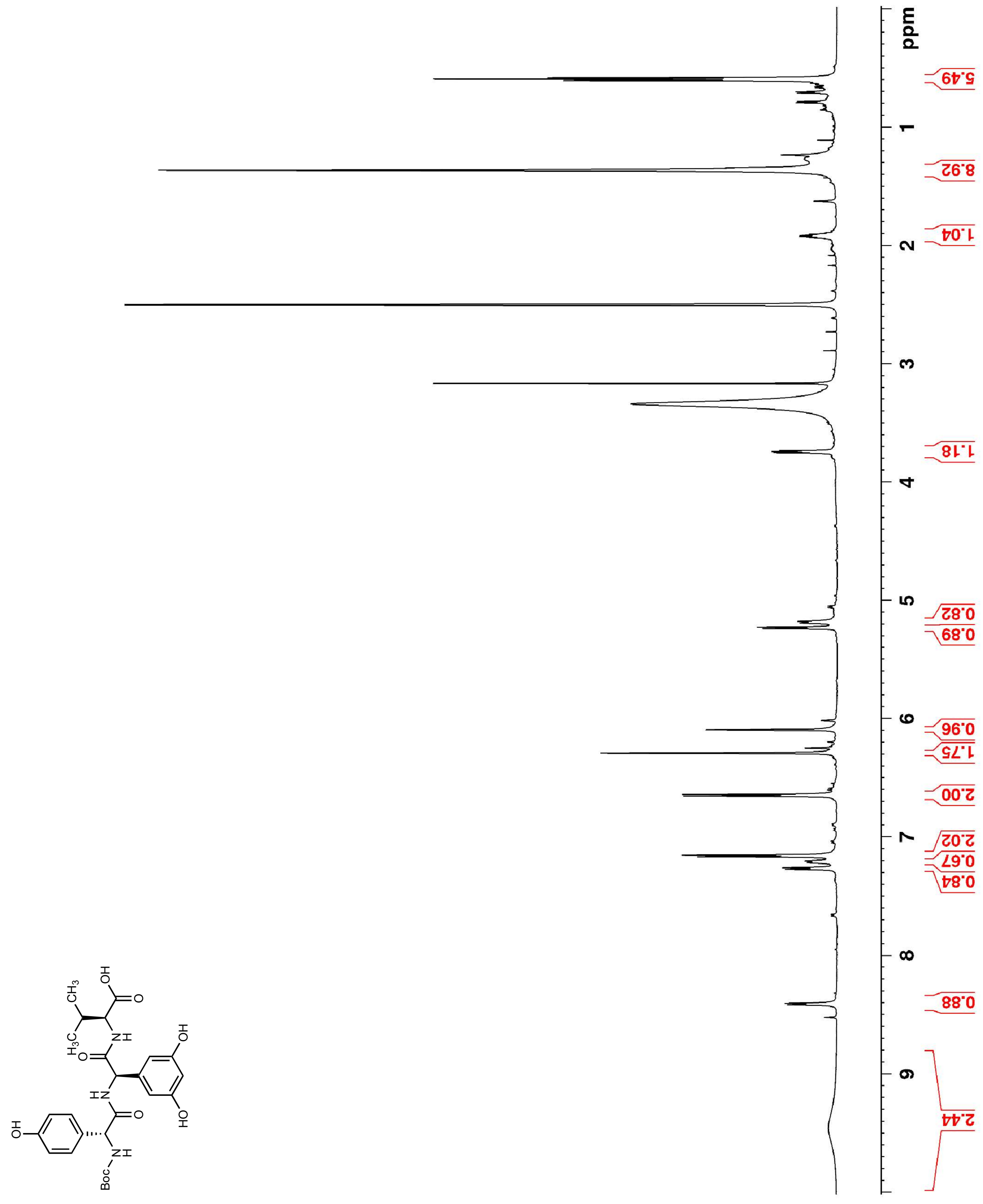

Figure S41. ^{13}C NMR (150 MHz, DMSO-d_6) of **28**

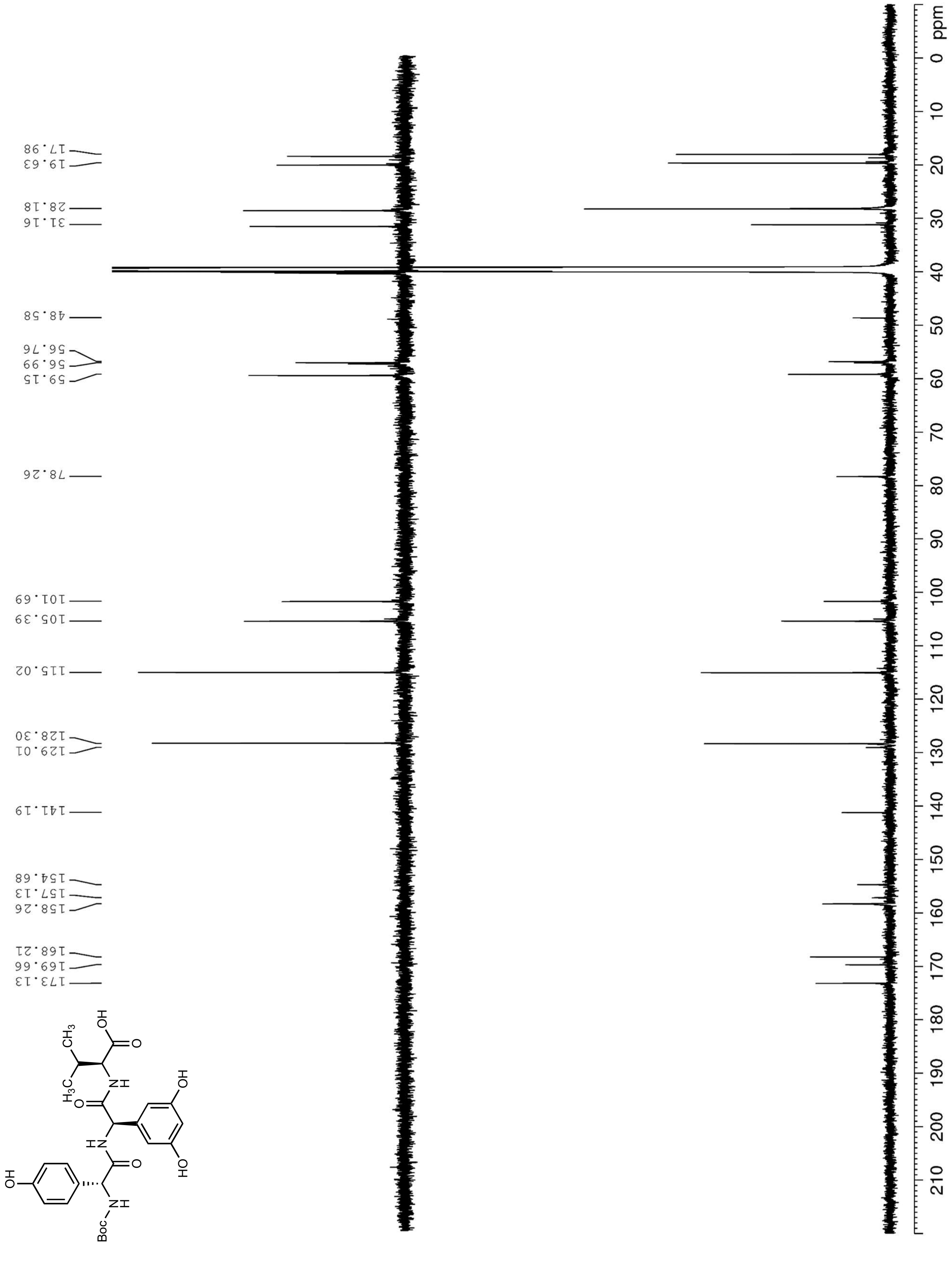

Figure S42. ^{1}H NMR (600 MHz, DMSO-d_6) of **30**

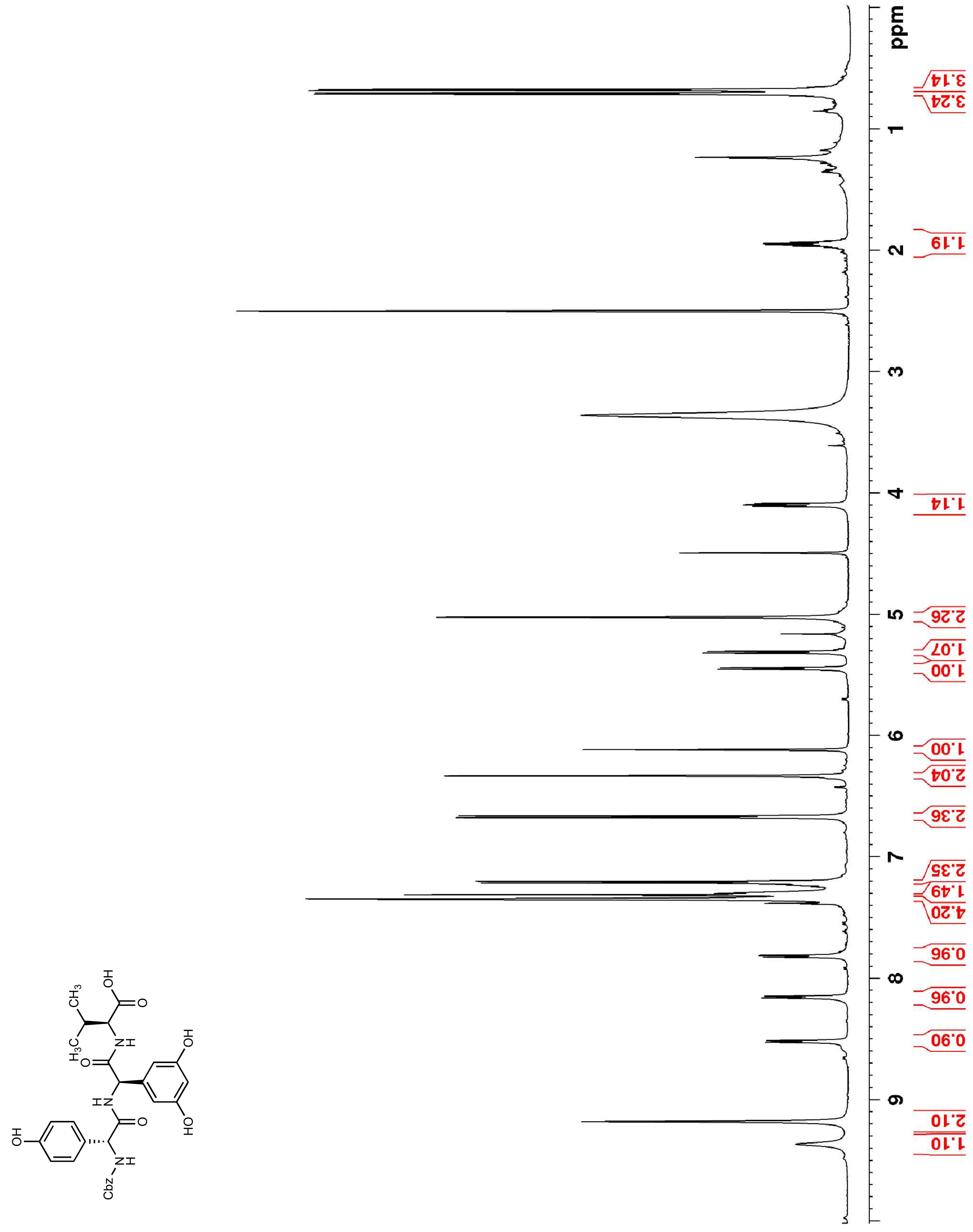

Figure S43. ^{13}C NMR (150 MHz, DMSO-d_6) of **30**

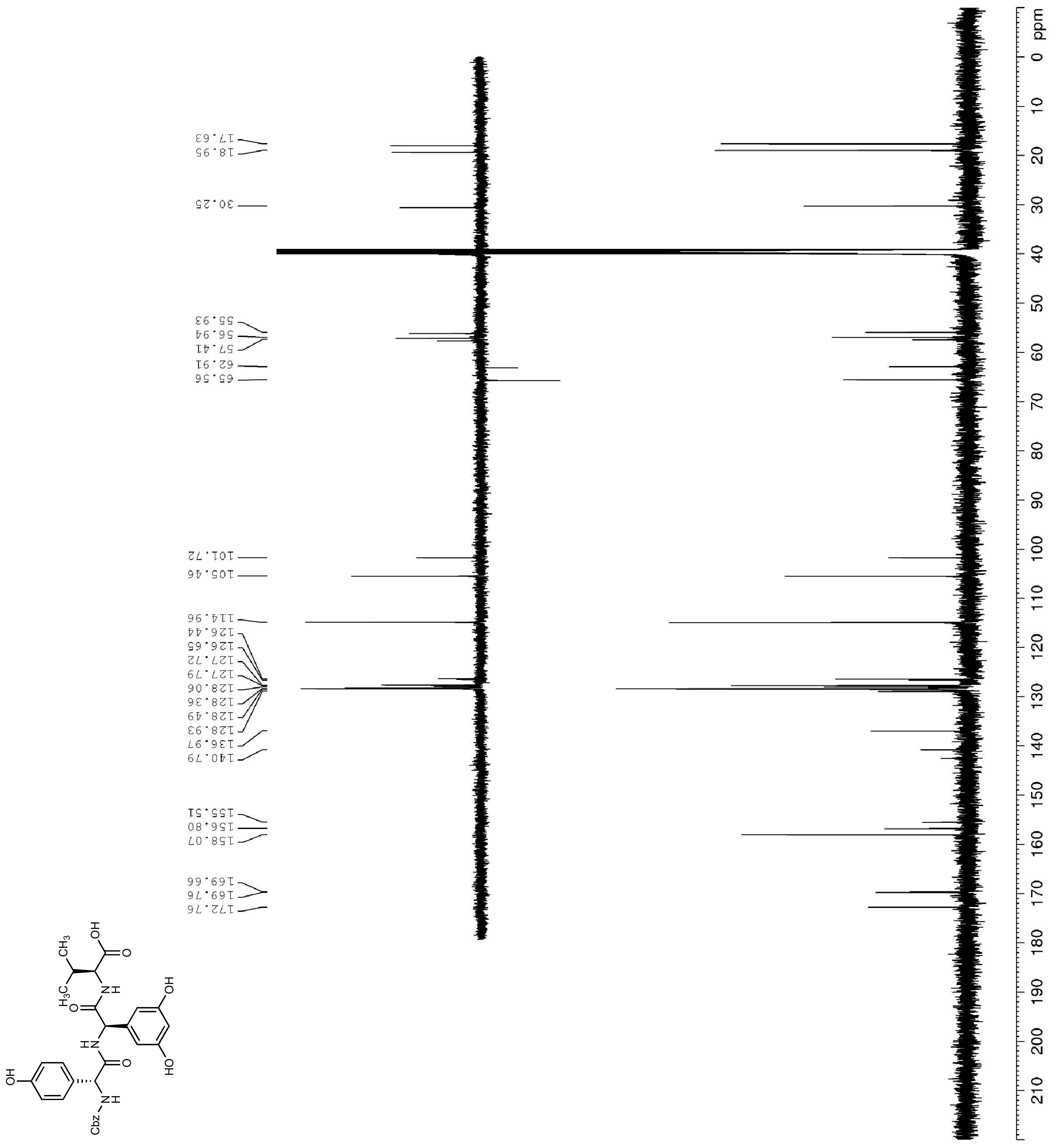

Figure S44. HPLC chromatogram of *rac*-**3**

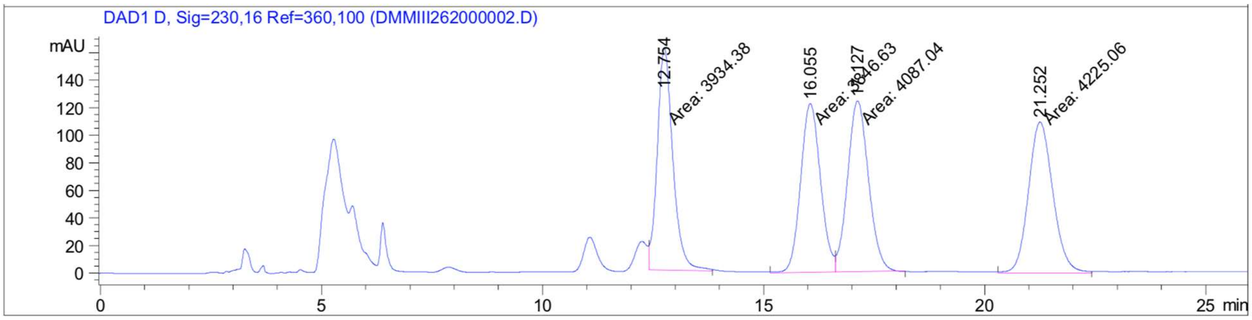

Signal 3: DAD1 D, Sig=230,16 Ref=360,100

Peak #	RetTime [min]	Type	Width [min]	Area [mAU*s]	Height [mAU]	Area %
1	12.754	MM	0.4062	3934.38135	161.44809	24.4476
2	16.055	MM	0.5225	3846.62842	122.69176	23.9023
3	17.127	MM	0.5495	4087.03735	123.95788	25.3962
4	21.252	MM	0.6414	4225.05713	109.79039	26.2538

Figure S45. HPLC *c*hromatogram of (*S*)-**3** before recrystallization

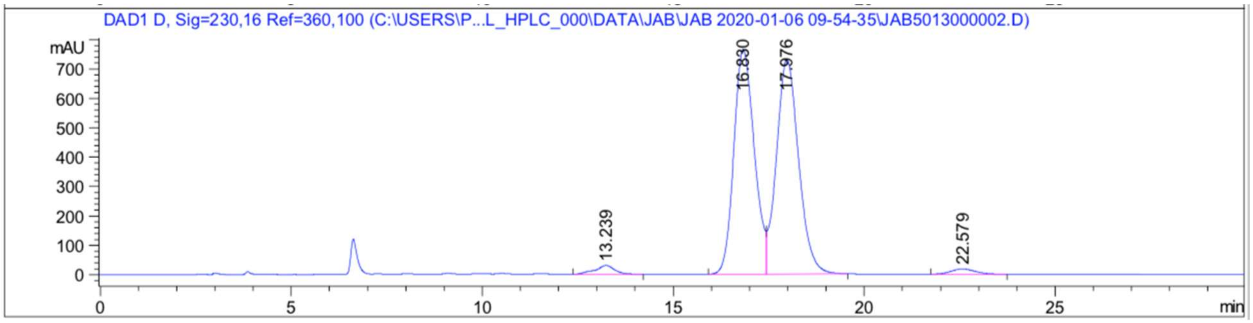

Signal 3: DAD1 D, Sig=230,16 Ref=360,100

Peak #	RetTime [min]	Type	Width [min]	Area [mAU*s]	Height [mAU]	Area %
1	13.239	BB	0.5121	1101.73474	30.16511	1.8342
2	16.830	BV	0.5790	2.85391e4	765.38269	47.5119
3	17.976	VB	0.6152	2.96228e4	730.29846	49.3161
4	22.579	BB	0.6122	803.56506	17.95339	1.3378

Figure S46. HPLC chromatogram of (*S*)-**3** after recrystallization

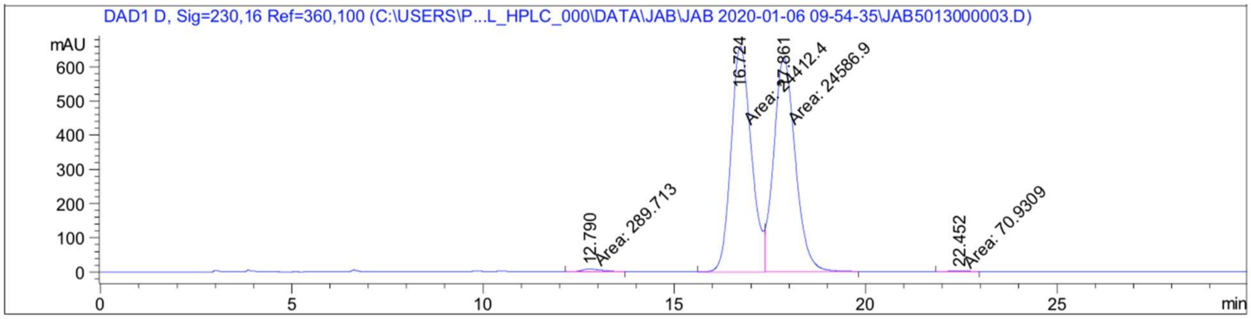

Signal 3: DAD1 D, Sig=230,16 Ref=360,100

Peak #	RetTime [min]	Type	Width [min]	Area [mAU*s]	Height [mAU]	Area %
1	12.790	MM	0.5860	289.71301	8.23931	0.5869
2	16.724	MM	0.6173	2.44124e4	659.16376	49.4580
3	17.861	MM	0.6536	2.45869e4	626.98694	49.8114
4	22.452	MM	0.6483	70.93092	1.82363	0.1437

Figure S47. HPLC chromatogram of *rac*-**20**

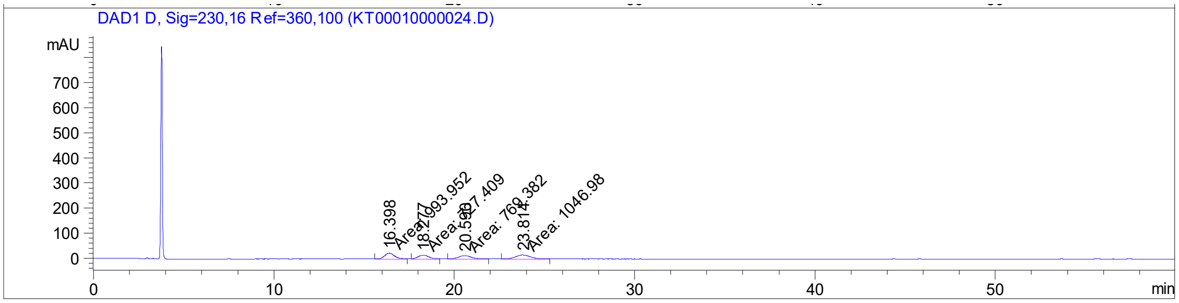

Signal 3: DAD1 D, Sig=230,16 Ref=360,100

Peak #	RetTime [min]	Type	Width [min]	Area [mAU*s]	Height [mAU]	Area %
1	16.398	MM	0.6814	993.95215	24.31329	28.0958
2	18.277	MM	0.7377	727.40894	16.43454	20.5615
3	20.590	MM	0.9052	769.38202	14.16669	21.7479
4	23.814	MM	1.0362	1046.97913	16.83998	29.5947

Figure S48. HPLC chromatogram of (*R*)-**20** before recrystallization

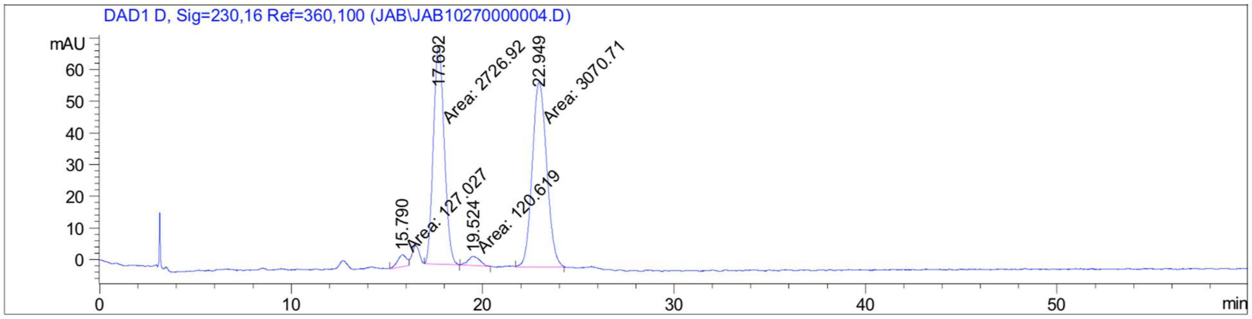

Signal 3: DAD1 D, Sig=230,16 Ref=360,100

Peak #	RetTime [min]	Type	Width [min]	Area [mAU*s]	Height [mAU]	Area %
1	15.790	MM	0.5668	127.02745	3.73526	2.1013
2	17.692	MM	0.6595	2726.91797	68.90873	45.1082
3	19.524	MM	0.6962	120.61935	2.88738	1.9953
4	22.949	MM	0.8761	3070.71484	58.41830	50.7952

Figure S49. HPLC chromatogram of (*R*)-**20** after recrystallization

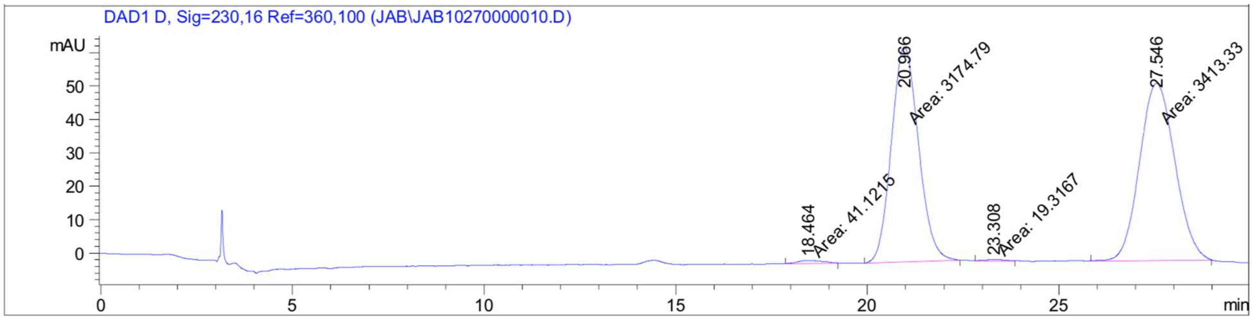

Signal 3: DAD1 D, Sig=230,16 Ref=360,100

Peak #	RetTime [min]	Type	Width [min]	Area [mAU*s]	Height [mAU]	Area %
1	18.464	MM	0.8090	41.12146	8.47149e-1	0.6185
2	20.966	MM	0.8219	3174.79297	64.38274	47.7515
3	23.308	MM	0.7233	19.31672	4.45122e-1	0.2905
4	27.546	MM	1.0716	3413.33496	53.08735	51.3394
